# Supplementary material for: ALBA proteins facilitate cytoplasmic YTHDF-mediated reading of m6A in Arabidopsis
Source: EMBO J. 2024 Nov 29;43(24):6626–55. doi: 10.1038/s44318-024-00312-0 (PMC11649824; doi:10.1038/s44318-024-00312-0)
Supplement: Supplementary file 1 — Appendix [file 44318_2024_312_MOESM1_ESM.pdf]

APPENDIX FOR

ALBA proteins facilitate cytoplasmic YTHDF-mediated reading of m^6A in *Arabidopsis*

Table of contents

Appendix Figure S1 2
Appendix Figure S2 3
Appendix Figure S3 4
Appendix Figure S4 6
Appendix Figure S5 7
Appendix Figure S6 8
Appendix Figure S7 9
Appendix Figure S8 10

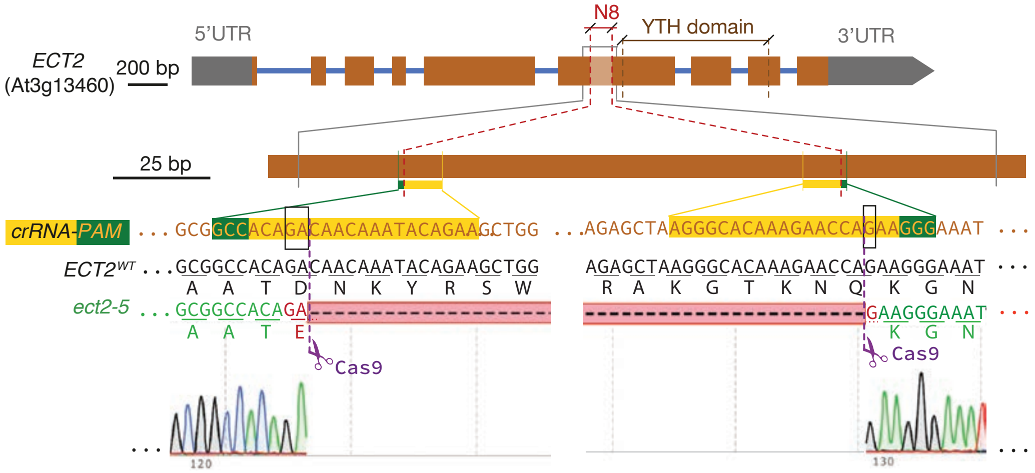

Appendix Figure S1. CRISPR-Cas9 engineering of the *ect2-5* deletion mutant.
Schematic representation of guide RNA design and the resulting in-frame chromosomal deletion matching nearly exactly the N8 element defined in transgenic deletion analysis[21]. crRNA, CRISPR-RNA, the sequence specificity components of the single guide RNAs used to induce chromosomal *ECT2* deletions; PAM, protospacer adjacent motif.

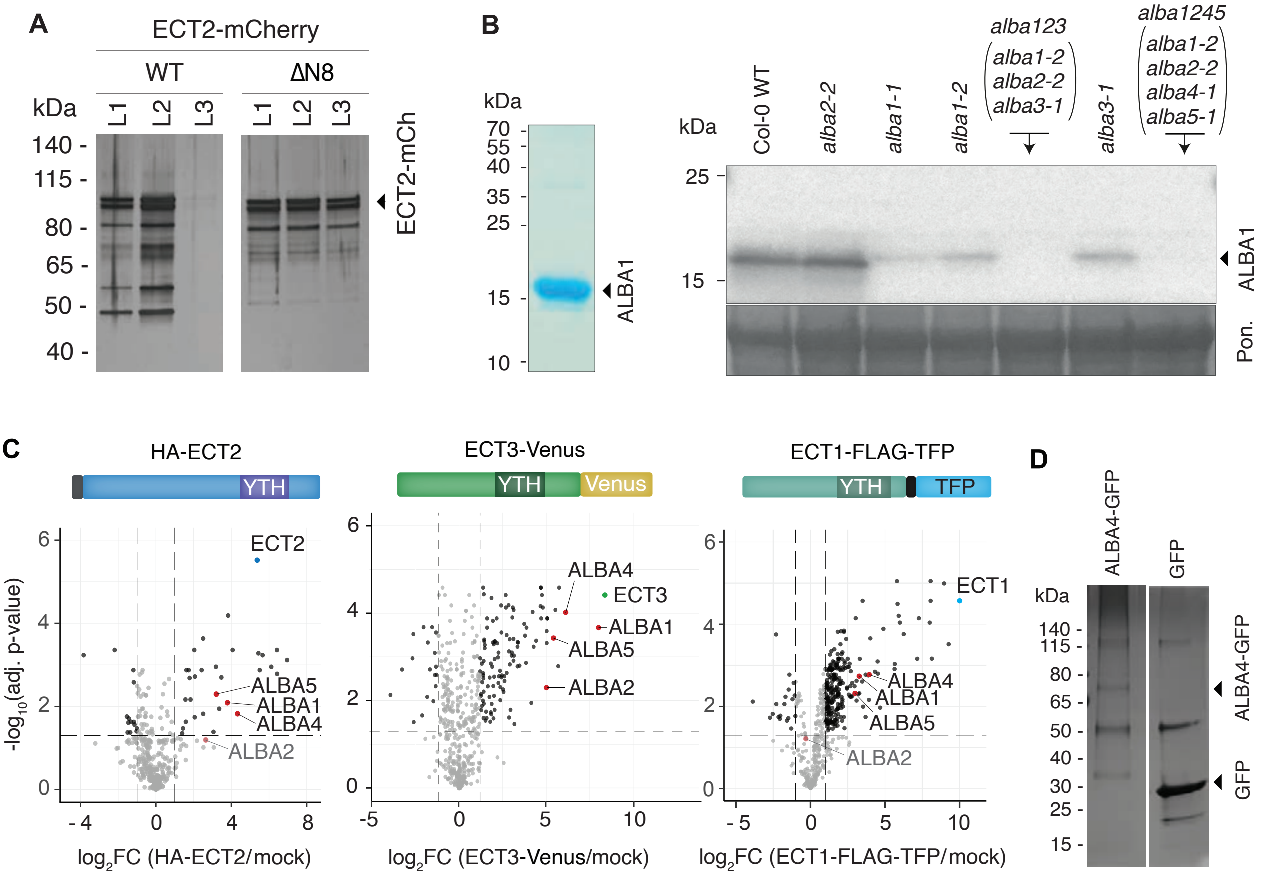

Appendix Figure S2. Characterization of ECT2-ALBA interaction (supporting data).

(A) Silver staining of aliquots of immunopurified fractions used for LS-MS/MS analysis of differential protein enrichment in ECT2-mCherry vs. ECT2$^{\Delta N8}$-mCherry purifications (supports **Figure 1G**).

(B) Left, coomassie stain of purified recombinant ALBA1 protein used for immunization of rabbits to produce antibodies. Right, western blot probed with the ALBA1 antibody. Ponceau staining is used as a loading control (supports **Figure 1H**). Note that although the *alba2-2* T-DNA allele used is not a full knockout, no ALBA2 protein is detectable in the *alba1245* mutant (see **Figure S5**).

(C) Volcano plot showing differential abundance of proteins immunopurified from total lysates of seedlings of the indicated transgenic lines compared to non-transgenic controls, highlighting the ALBA proteins identified in each case. Left, HA-ECT2[7] was purified with anti-HA beads; center, ECT3-Venus[7] was purified with GFP-trap; right, ECT1-TFP[20] was purified with GFP-trap. In all cases, proteins were identified and quantified by LC-MS/MS, and statistical significance was determined using empirical Bayes statistics with Benjamini–Hochberg adjusted p-values. These data have been published previously with no specific mention of ALBA proteins[21].

(D) Silver staining of aliquots of immunopurified fractions used for LS-MS/MS analysis of differential enrichment in ALBA4-GFP and GFP purifications (supports **Figure 1I**).

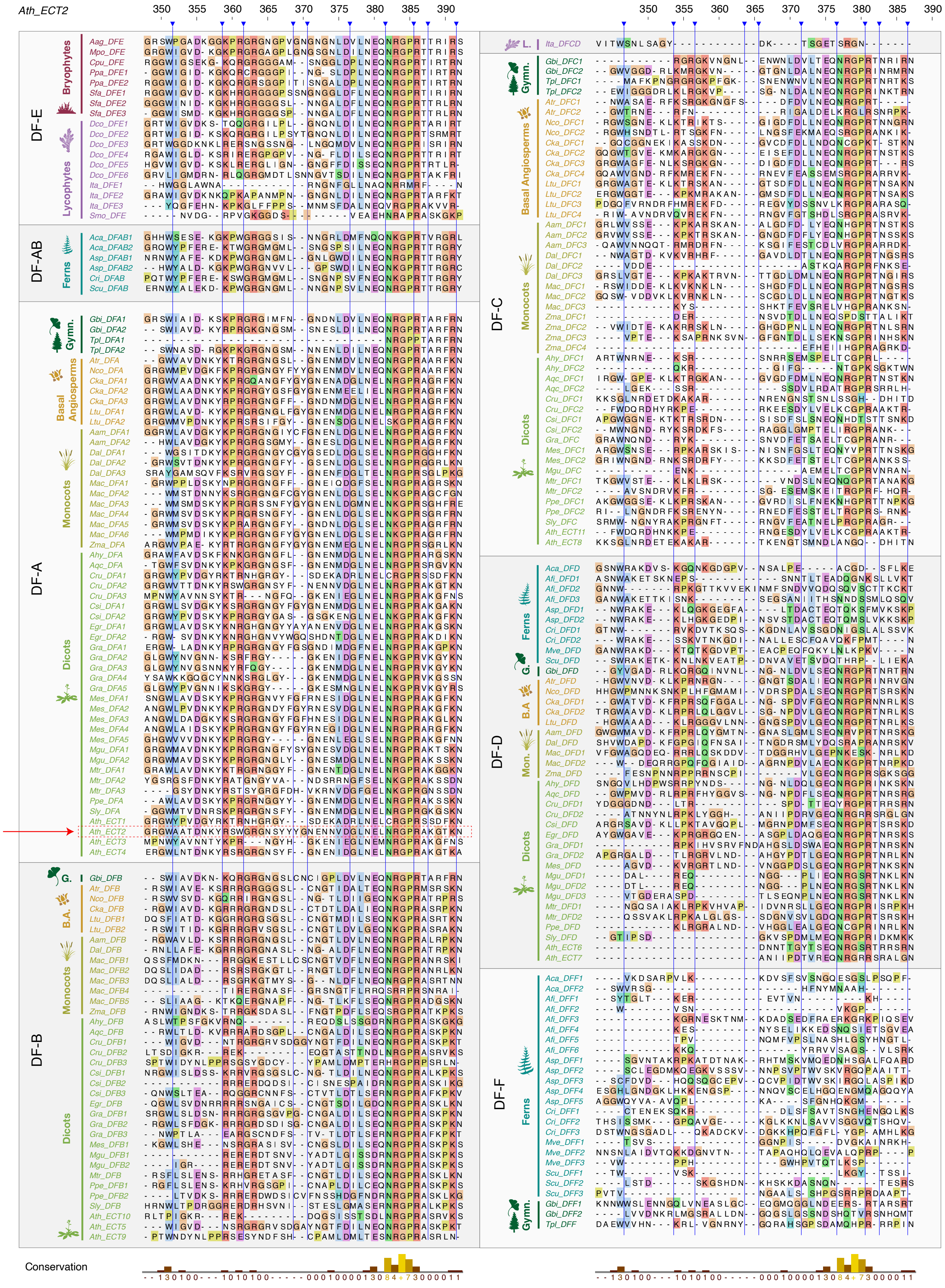

Appendix Figure S3. Amino acid sequence alignment of the N8 region of YTHDF proteins in 36 species of land plants
(continues on the next page)

(continues from the previous page, **Appendix Figure S3**)

Trimmed amino acid sequence alignment of all YTHDF proteins in the following 36 species of land plants:
Bryophytes (hornworts, liverworts and mosses): *Anthoceros agrestis (Aag), Marchantia polymorpha (Mpo), Sphagnum fallax (Sfa), Ceratodon purpureus (Cpu), Physcomitrium patens (Ppa)*
Lycophytes (fern allies): *Isoetes taiwanensis (Ita), Diphasiastrum complanatum (Dco), Selaginella moellendorfii (Smo)*
Ferns: *Alsophila spinulosa (Asp), Salvinia cucullata (Scu), Marsilea vestita (Mve), Azolla filliculoides (Afi) Adiantum capillus-veneris (Aca), Ceratopteris richardii (Cri)*
Gymnosperms: *Ginkgo bioloba (Gbi), Thuja plicata (Tpl)*
Basal Angiosperms and Magnoliids: *Amborella trichopoda (Atr), Nymphaea colorata (Nco), Cinnamomum kanehirae (Cka), Liriodendron tulipifera (Ltu)*
Monocots: *Acorus americanus (Aam), Zostera marina (Zma), Musa acuminata (Mac), Dioscorea alata (Dal)*
Dicots: *Aquilegia coerulea (Aqc), Amaranthus hypochondriacus (Ahy), Solanum lycopersicum (Sly), Mimulus guttatus (Mgu), Citrus sinensis (Csi), Gossypium raimondii (Gra), Prunus persica (Ppe), Manihot esculenta (Mes), Eucalyptus grandis (Egr), Medicago truncatula (Mtr), Capsella rubella (Cru), Arabidopsis thaliana (Ath)*

The alignment is trimmed at both ends to leave only the region corresponding N8 motif of the ECT2 IDR and upstream adjacent sequences. Blue arrowheads and vertical lines indicate points of insertions present in a few proteins and not present in ECT2, that have been removed for clarity. The *Ath* ECT2 sequence is highlighted with red dashed lines and an arrow, and the amino acid numbers are indicated on top. Conservation scores indicated below the alignment correspond to all the sequences displayed in the two columns, and are calculated by Jalview (see methods). The different taxons and protein clades are colour-coded to facilitate interpretation. Notice that the IDR SLiM 'YAIM' in N8 is extremely conserved in all land plant YTHDF proteins except for the DF-F clade (exclusive of ferns and gymnosperms), and fern DF-D proteins, but it is present in the fern DF-AB paralogues.

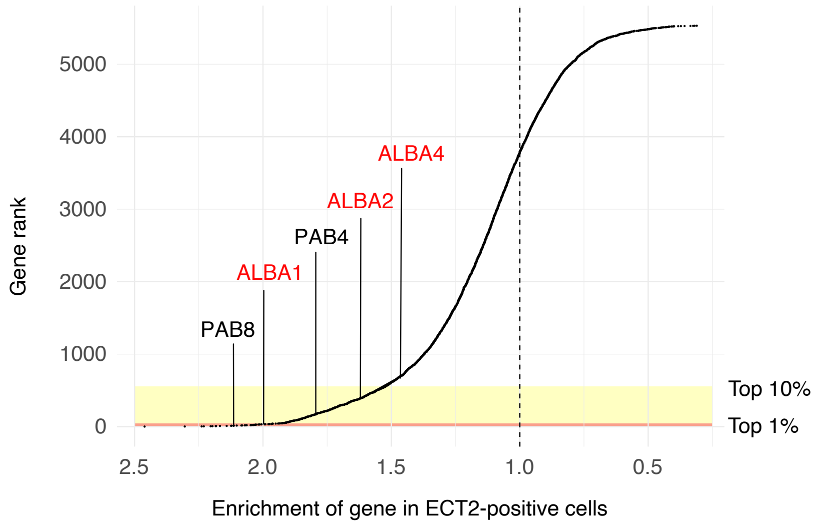

Appendix Figure S4. Co-expression analysis of ECT2 and ALBA proteins.
Odds ratio represents correspondence between ECT2-positive cells and cells expressing a given gene. Analysis controls for differences in UMI counts between ECT2-positive and ECT2-negative cells, and considers only genes expressed in between 20 and 80% of cells. Top 1% and 10% represents top genes out of those tested.

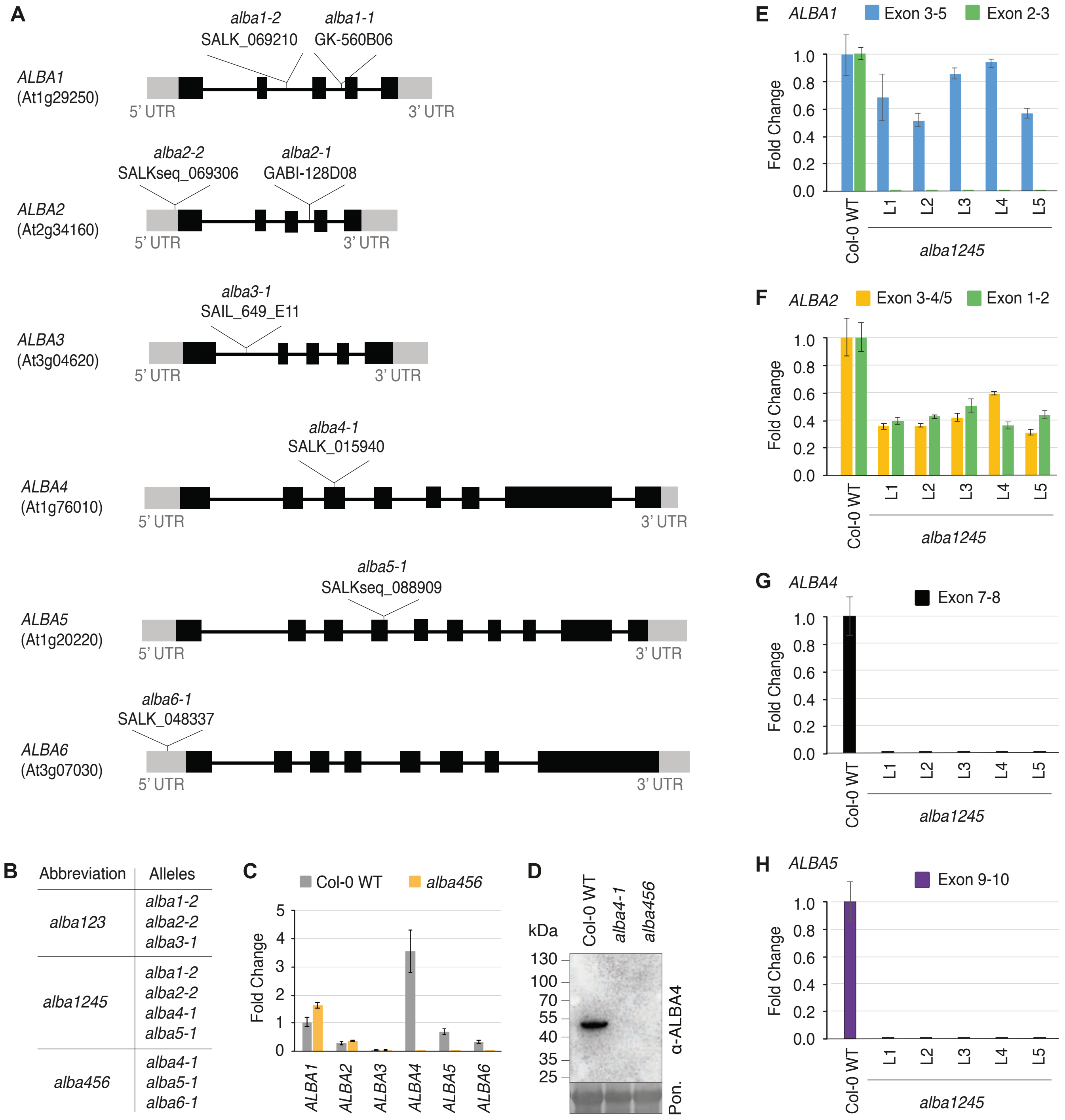

Appendix Figure S5. Characterization of *ALBA* mutants (supporting data).
(A) Schematic representation of *ALBA1-ALBA6* loci with the sites of T-DNA insertions indicated.
(B) Abbreviations of higher order *alba* mutants.
(C) *ALBA* mRNA levels measured by qPCR in Col-0 WT and *alba456*. Each measurement represents three biological replicates, with each replicate being composed of three individual plants. RNA levels were normalized to *CYCLOPHILIN* (At2g29960). Error bars represent the standard deviation of the means.
(D) Western blot probed with an ALBA4 antibody raised against synthetic ALBA4 peptides (see Methods). Ponceau (Pon.) staining is used as a loading control.
(E-H) *ALBA1, ALBA2, ALBA4* and *ALBA5* mRNA levels measured by qPCR in Col-0 WT and five individual *alba1245* plants, using primers spanning the indicated exons. RNA levels were normalized to *ACTIN2* (At3g18780). Error bars represent the standard deviation of the means.

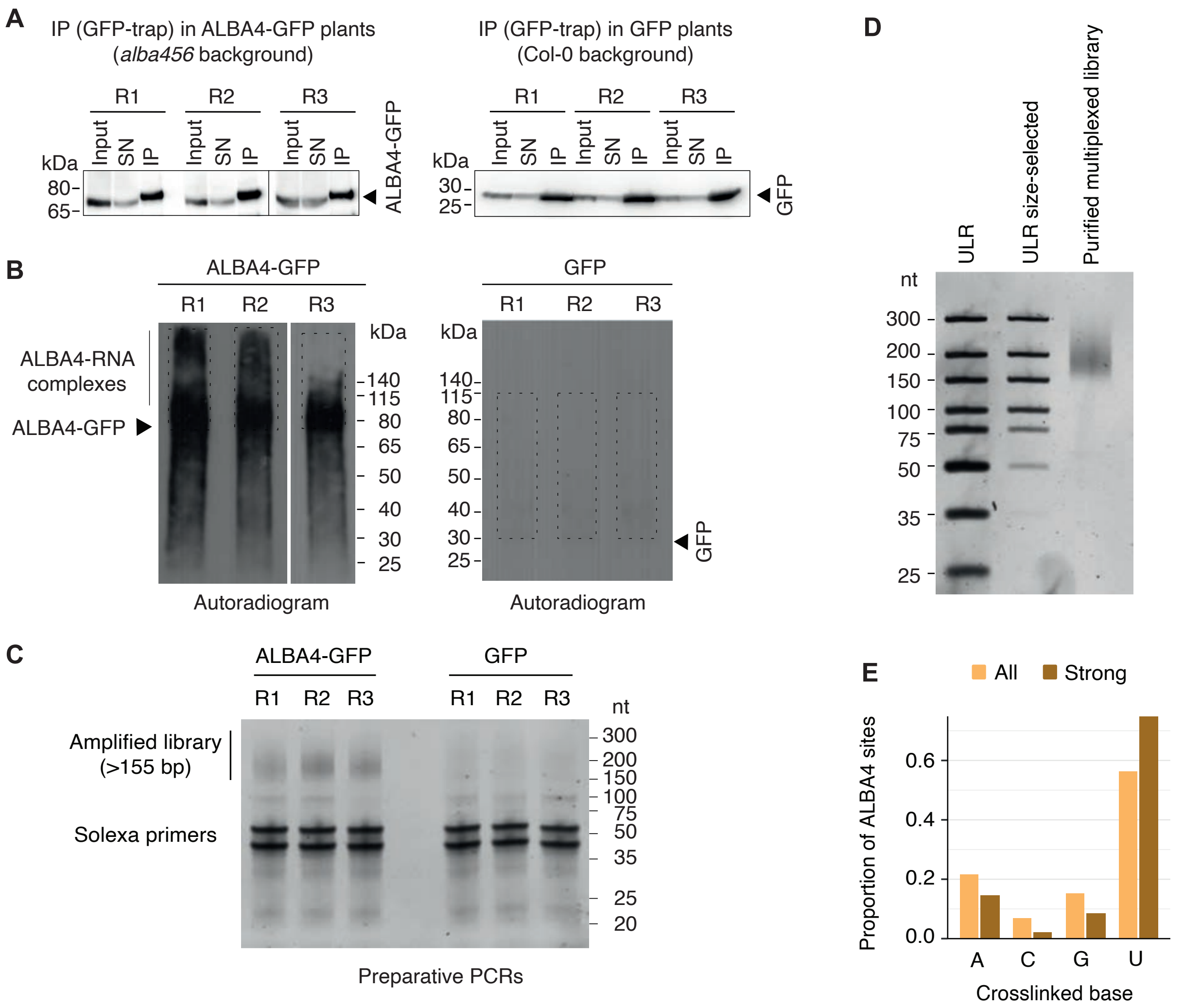

Appendix Figure S6. ALBA4 target identification using iCLIP2 (supporting data).

(A) Western blots against GFP after UV crosslinking and immunoprecipitation of RNA-protein complexes with GFP-trap beads in ALBA4-GFP plants (in *alba456* background) and GFP plants (in Col-0 background) (three replicates each). Presence of the respective protein is shown in the input (IN), supernatant (SN) after precipitation, and IP fraction.

(B) Autoradiogram of RNA-protein complexes from ALBA4-GFP (left) and GFP plants (right) after UV crosslinking and immunoprecipitation with GFP-Trap beads (three replicates each). Marker positions and the location of the ALBA4-GFP-RNA adducts are indicated. Dashed rectangles indicate the regions that were excised from the membrane. Autoradiograms of ALBA4-GFP were developed after 4 h exposure, while those of GFP were developed after overnight exposure.

(C) Gel electrophoresis of PCR-amplified iCLIP2 cDNA libraries (3 replicates each) visualized on a 12% polyacrylamide gel. A size standard indicates the fragment sizes. Libraries are visible as a smear at 155 nt and above.

(D) Gel electrophoresis of purified iCLIP2 cDNA library after multiplexing, visualized on a 12% polyacrylamide gel. The cDNA library, alongside with the ultra-low range (ULR) molecular weight ladder, is subjected to size selection with ProNex beads to remove solexa primers. A ratio of ~15:1 between the 150-nt band and the 75-nt band of the URL ladder indicates efficient purification.

(E) Proportion of ALBA4 iCLIP2 crosslink sites according to the reference nucleotide, for both full and strong sets.

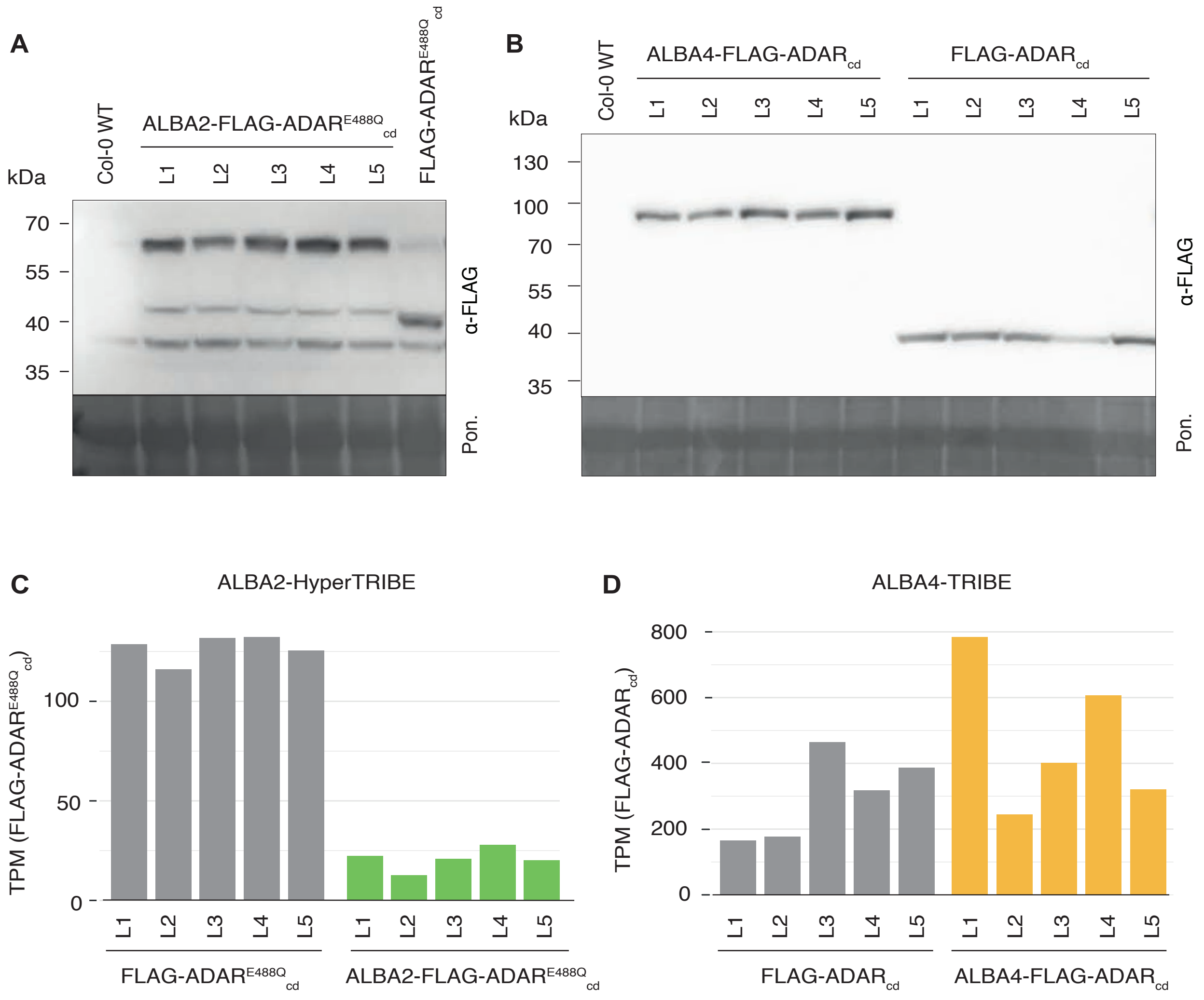

Appendix Figure S7. ALBA2 and ALBA4 target identification by TRIBE and HyperTRIBE (supporting data).

(A) Western blot of the independent lines selected for HyperTRIBE analysis of ALBA2. Ponceau (Pon.) staining is used as a loading control. The same blot (samples Col-0 WT and ALBA2-FLAG-ADAR^{E488Q} L1-L5) is also shown in Fig EV4D, because the same ALBA2-FLAG-ADAR^{E488Q} -expressing lines were used for definition of ALBA2 HyperTRIBE targets (comparison to the line expressing free FLAG-ADAR^{E488Q}) and analysis of differential editing dependent on presence of ECT2, ECT3 and ECT4 (comparison to ALBA2-FLAG-ADAR^{E488Q}-expressing lines in the *Gte234* mutant background).

(B) Western blot analysis of the independent lines selected for TRIBE analysis of ALBA4. Ponceau (Pon.) staining is used as a loading control.

(C) Transcripts per million of FLAG-ADAR$^{E488Q}_{cd}$ detected in lines used for HyperTRIBE (ALBA2-HT).

(D) Transcripts per million of FLAG-ADAR$_{cd}$ detected in lines used for TRIBE (ALBA4-TRIBE).

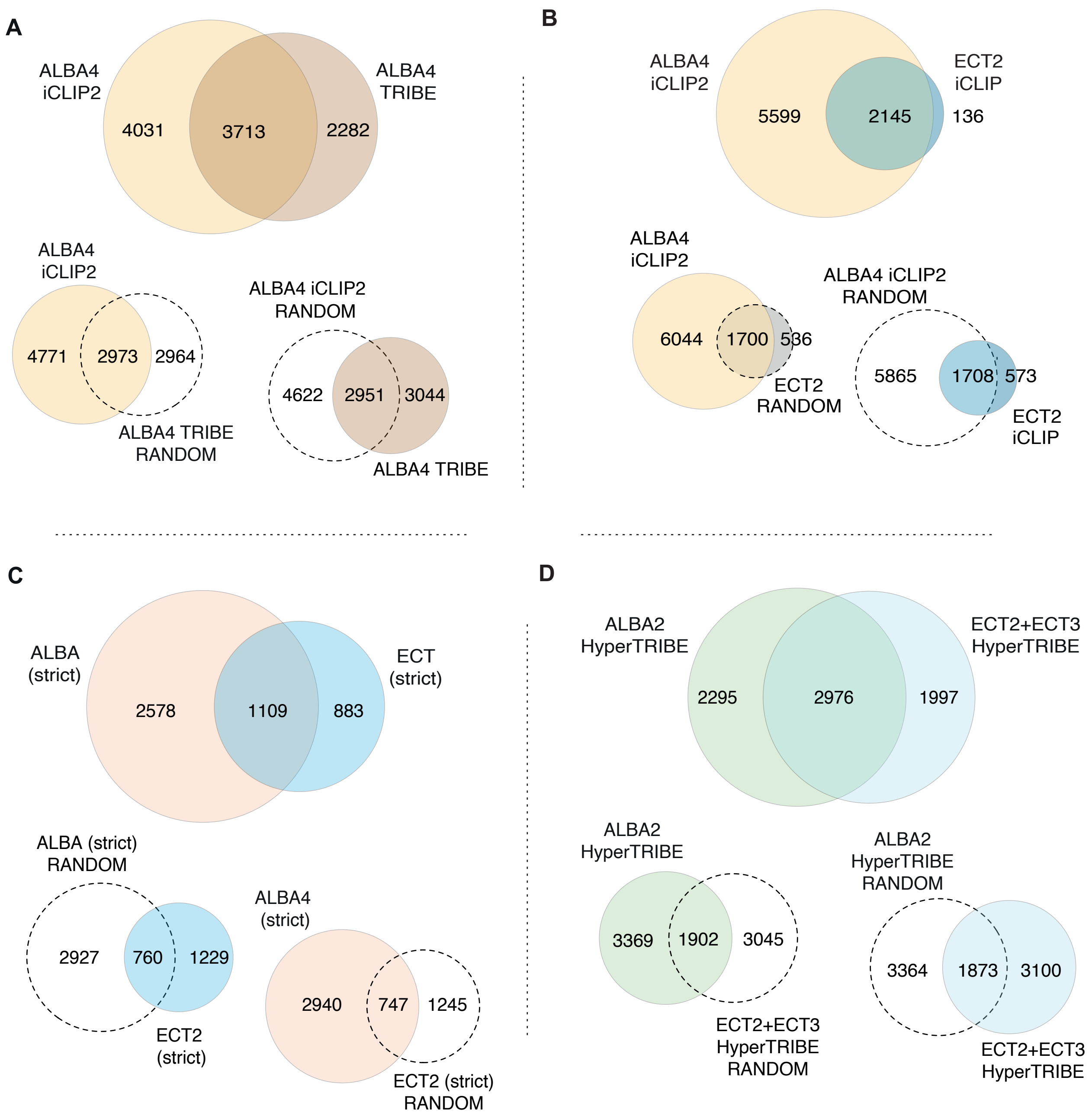

Appendix Figure S8. Analyses of the overlap between ALBA2, ALBA4, ECT2 and ECT3 target sets determined by (Hyper)TRIBE and/or iCLIP.
Venn diagrams showing the overlap between the indicated target sets. In all cases, smaller Venn diagrams with one dashed circle indicate the overlap obtained if one of the sets used in the comparison were a randomly selected group of genes with a similar expression distribution to the true target set.
(A) Overlap between iCLIP2-defined and TRIBE-defined ALBA4 target sets.
(B) Overlap between iCLIP2-defined ALBA4 and iCLIP-defined ECT2 target sets.
(C) Overlap between strict ALBA4 (iCLIP2 + TRIBE support) and strict ECT2 (iCLIP + HyperTRIBE support) target sets.
(D) Overlap between ALBA2 and ECT2/3 HyperTRIBE target sets (only aerial tissues).
