# Supplementary material for: ALBA proteins facilitate cytoplasmic YTHDF-mediated reading of m6A in Arabidopsis
Source: EMBO J. 2024 Nov 29;43(24):6626–55. doi: 10.1038/s44318-024-00312-0 (PMC11649824; doi:10.1038/s44318-024-00312-0)
Supplement: Supplementary file 9 — Dataset EV7 [file 44318_2024_312_MOESM9_ESM.docx]

**Dataset EV7. Protein sequence alignment**

Amino acid sequence alignment of YTHDF proteins of 34 species of land plants used to generate the N8 Logo (YAIM motif) in Figure 2A

The species used and their abbreviations are the following:

**0. Bryophytes (hornworts and liverworts)**: Anthoceros agrestis (Aag), Marchantia polymorpha (Mpo)

**1. Bryophytes (mosses)**: Sphagnum fallax (Sfa), Ceratodon purpureus (Cpu), Physcomitrium patens (Ppa),

**2. Lycophytes**: Isoetes taiwanensis (Ita), Diphasiastrum complanatum (Dco), Selaginella moellendorfii (Smo)

**3. Ferns**: Alsophila spinulosa (Asp), Salvinia cucullata (Scu), Adiantum capillus-veneris (Aca), Ceratopteris richardii (Cri)

**4. Gymnosperms**: Ginkgo bioloba (Gbi), Thuja plicata (Tpl)

**5. Basal Angiosperms**: Amborella trichopoda (Atr), Nymphaea colorata (Nco)

**6. Magnoliids**: Cinnamomum kanehirae (Cka), Liriodendron tulipifera (Ltu)

**7. Monocots**: Acorus americanus (Aam), Zostera marina (Zma), Musa acuminata (Mac), Dioscorea alata (Dal)

**8. Dicots**: Aquilegia coerulea (Aqc), Amaranthus hypochondriacus (Ahy), Solanum lycopersicum (Sly), Mimulus guttatus (Mgu),

Citrus sinensis (Csi), Gossypium raimondii (Gra), Prunus persica (Ppe), Manihot esculenta (Mes), Eucalyptus grandis (Egr),

Medicago truncatula (Mtr), Capsella rubella (Cru)

**9. Dicots**: Arabidopsis thaliana (Ath)

Note: Additionally, for the alignment in Appendix Figure S3 that includes the DF-F clade and fern DF-Ds, the additional species of ferns were included:

Marsilea vestita (Mve), Azolla filliculoides (Afi)

**ALIGNMENT**

DFE_0_Aag_DFE G-NLDVLNEQNRGPRTTRIRSLR

DFE_0_Mpo_DFE G-NLDILNEQNRGPRTTRIRSQR

DFE_1_Cpu_DFE G-GLDPLNEQNRGPRTIRTRNQR

DFE_1_Ppa_DFE1 G-SLDPLNEQNRGPRTIRTRNQR

DFE_1_Ppa_DFE2 G-ALDPLNEQNRGPRTIRTRNQR

DFE_1_Sfa_DFE1 G-GLDFLNEQNRGPRTIRIRNQL

DFE_1_Sfa_DFE2 G-ALDFLNEQNRGPRTTRLRNQR

DFE_1_Sfa_DFE3 G-ALDFLNEQNRGPRTTRLRNQR

DFE_2_Dco_DFE1 Q-NLDILNEQNRGPRTARIRTQR

DFE_2_Dco_DFE2 Q-NLDILNEQNRGPRTSRMRTQR

DFE_2_Dco_DFE3 G-QMDVLNEQNRGPRISRIRNQR

DFE_2_Dco_DFE4 G-FLDILSEQNRGPRTTRIRTVR

DFE_2_Dco_DFE5 G-FFDISSEQNRGPRTRTLR---

DFE_2_Dco_DFE6 GVTSDILNEQNRGPRTAKFRIQG

DFE_2_Ita_DFE1 G-NFGLLNAQNRRMRFNESTLRM

DFE_2_Ita_DFE2 G-NLDILNEQNRGPRTARFKTQR

DFE_2_Ita_DFE3 M-SFDALNEQVRGPRAKVVR---

DFE_2_Smo_DFE S----VEAEHNRAPRASKGKPQR

DFAB_3_Aca_DFAB1 G-RLDMFNQQNKGPRTVRGRLHR

DFAB_3_Aca_DFAB2 P-SWDILNEQNRGPRTTRGRYQR

DFAB_3_Asp_DFAB1 G-NLGILNEQNRGPRTTRGRYQR

DFAB_3_Asp_DFAB2 GPSWDILNEQNKGPRTTRGRCQR

DFAB_3_Cri_DFAB P-SWDVFNEQNKGPRTTRGRYQR

DFAB_3_Scu_DFAB P-SWDVLNEQNRGPRTTRGRYQR

DFA_4_Gbi_DFA1 D-NLDVLNEQNRGPRTARFRNQQ

DFA_4_Gbi_DFA2 E-SLDILNEQNRGPRTARFRNQR

DFA_4_Tpl_DFA1 --------MENRGPPTARFRNQR

DFA_4_Tpl_DFA2 NENLDILNEQNRGPRTARFRNQR

DFA_5_Atr_DFA NENMDVLNEQNRGPRAARFKNQR

DFA_5_Nco_DFA E-NMDVLNEQNKGPRAARFKNQR

DFA_6_Cka_DFA1 E-NADGLNELNRGPRAGRFKNQR

DFA_6_Cka_DFA2 E-NMEELIELNRGPRAGRFKNQK

DFA_6_Cka_DFA3 D-NMDGLNELNRGPRAGRFKNQK

DFA_6_Ltu_DFA1 E-NMDGLNELNRGPRAGRFKNQK

DFA_6_Ltu_DFA2 E-NSDGLNELSKGPRAGRFKNQK

DFA_7_Aam_DFA1 E-NLDGLNELNRGPRAGRFKNQK

DFA_7_Aam_DFA2 E-SLDGLNELNRGPRAGRFKNPK

DFA_7_Dal_DFA1 E-DLDGLSELNRGPRGGHFKNTK

DFA_7_Dal_DFA2 E-NLDGLSELNRGPRGGRLKNTK

DFA_7_Dal_DFA3 E-NFDGLTELNRGPRSGLPKGIK

DFA_7_Mac_DFA1 E-IQDGFSELNRGPRAGRSKNQK

DFA_7_Mac_DFA2 E-NLDGLSELNKGPRAGCFGNQK

DFA_7_Mac_DFA3 E-NLDGMNELNKGPRSGHFREQK

DFA_7_Mac_DFA4 E-NLDGLSELNKGPRSGRFRNQK

DFA_7_Mac_DFA5 D-NLDGLSELNKGPRAGRFRNQR

DFA_7_Mac_DFA6 E-NLDGLSELNKGPRAGRFRNQV

DFA_7_Zma_DFA E-NMEGLNELNRGPRSGRLKNQK

DFA_8_Ahy_DFA E-NMDGLNELNRGPRARGSKNTR

DFA_8_Aqc_DFA E-NMDGLNELNRGPRSRGVKNQK

DFA_8_Cru_DFA1 DEKADRLNELCRGPRSSDFKNPQ

DFA_8_Cru_DFA2 ENNVDGLNELNRGPRAKGTKNQK

DFA_8_Cru_DFA3 E-NIEGLNELNRGPRAKGFNSQE

DFA_8_Csi_DFA1 E-NMDGLNELNRGPRAKGAKNQK

DFA_8_Csi_DFA2 E-NVDGLNELNKGPRAKGFKNQE

DFA_8_Egr_DFA1 E-NVDGLNELNRGPRAKGSKNQK

DFA_8_Egr_DFA2 D-NTDGLNELNRGPRAKDIKNQK

DFA_8_Gra_DFA1 D-IMDGFNELNRGPRAKGPKNQK

DFA_8_Gra_DFA2 E-NIDGFNELNKGPRVKGYKNKD

DFA_8_Gra_DFA3 E-NMDGLNELNRGPRVKGYKNKD

DFA_8_Gra_DFA4 E-NMDGLNELNKGPRVKGSSNKD

DFA_8_Gra_DFA5 KESMNGLNELNKGPRVKGYRSQD

DFA_8_Mes_DFA1 E-SIDGLNELNRGPRAKGFKNPK

DFA_8_Mes_DFA2 E-SVDGLNELNRGPRAKGFKNQK

DFA_8_Mes_DFA3 E-SIDGLNELNRGPRAKGLKNQK

DFA_8_Mes_DFA4 E-GIDGLNELNRGPRARGFKNQK

DFA_8_Mes_DFA5 E-NLEGLSELNRGPRAKGFNNQT

DFA_8_Mgu_DFA1 E-SVDGLNELNRGPRAKTSKNQK

DFA_8_Mgu_DFA2 E-SMDGLNELNRGPRAKSSKNTK

DFA_8_Mtr_DFA1 E-NTDGLNELNRGPRAKGGKNQK

DFA_8_Mtr_DFA2 G---DGFSELNKGPRAAKSSDNK

DFA_8_Mtr_DFA3 --NVDGFGELNKGPRGNSSDDKN

DFA_8_Ppe_DFA E-NMDGLNELNRGPRAKSSKNQK

DFA_8_Sly_DFA E-NMDGLNELNRGPRGKGSKNQK

DFA_9_Ath_ECT1 E-KADRLNELCRGPRSSDFKNPQ

**DFA_9_Ath_ECT2 ENNVDGLNELNRGPRAKGTKNQK**

DFA_9_Ath_ECT3 E-NIEGLNEMNRGPRAKGFNSQD

DFA_9_Ath_ECT4 E-NIDGLNELNRGPRAKGTKATE

DFB_4_Gbi_DFB G-PLDVLNEQNRGPRTARFRNQQ

DFB_5_Atr_DFB G-TIDALTEQNRGPRMSRSKNPQ

DFB_5_Nco_DFB G-TLDIIGEQNKGPRATRPRDQE

DFB_6_Cka_DFB D-TLDALIEQNRGPRASRPKNPS

DFB_6_Ltu_DFB1 G-TMDILSEQNKGPRASRTKNST

DFB_6_Ltu_DFB2 G-TLDVLGEQNKGPRASRAKNQT

DFB_7_Aam_DFB G-TLDVLNEQNRGPRALRPKNPS

DFB_7_Dal_DFB G-ALDFLNEQNRGPRATRPKNPI

DFB_7_Mac_DFB1 G-TVDFLNEQNRGPRANRSKIQM

DFB_7_Mac_DFB2 G-TLDFLNEQNRGPRANRLKNQM

DFB_7_Mac_DFB3 G-NLDFLNEQNRGPRASRTNNQM

DFB_7_Mac_DFB4 G-THGFLRRQSRRPWTNRSNNRA

DFB_7_Mac_DFB5 G-TLKFLSEQNRGPRADGSKNRA

DFB_7_Zma_DFB G-TPDFLSEQSRGPRATKPKSSE

DFB_8_Ahy_DFB DSLFASSGDRNRGPRASKGKGKG

DFB_8_Aqc_DFB G-ALDILSEQNRGPRASKPKNQG

DFB_8_Cru_DFB1 G-TFDILNEQNRGPRASKPKTQV

DFB_8_Cru_DFB2 STTNDLYG--NRGPRASRVKSKN

DFB_8_Cru_DFB3 PAMLDMPTERHRGPRPSRLN---

DFB_8_Csi_DFB1 G-TLDILNEQNRGPRALKPKSQM

DFB_8_Csi_DFB2 E-SPA--IDRNRGPRASKIKGKS

DFB_8_Csi_DFB3 V-TLDTLSERNRGPRAFKPK-VR

DFB_8_Egr_DFB G-TSDILGDQNRGPRASKLKNHA

DFB_8_Gra_DFB1 G-ALDILSEQNRGPRASKPKNQI

DFB_8_Gra_DFB2 G-ALDILSEQNRGPRASKPKNQI

DFB_8_Gra_DFB3 V-TLDTLSERNRGPRAFKPKTQI

DFB_8_Mes_DFB1 G-SLDILSEQNRGPRASKPKSQN

DFB_8_Mes_DFB2 V-ALDTLGERNRGPRAFKPRSKT

DFB_8_Mgu_DFB1 D-TLGISSDRNRGPRASKPKSKS

DFB_8_Mgu_DFB2 D-TLGISSDRNRGPRASKPKSKS

DFB_8_Mtr_DFB G-TLDILSEQNRGPRASKLKNHI

DFB_8_Ppe_DFB1 A-PLDILCEQNRGPRASKPKSQI

DFB_8_Ppe_DFB2 S-SHDGFNDRNRGPRASKLKGKN

DFB_8_Sly_DFB E-SLGMASERNRGPRALKPKSKA

DFB_9_Ath_ECT10 SSTSDLYG--NRGPRASRVKSKN

DFB_9_Ath_ECT5 G-TFDILNEQNRGPRASKPKTQV

DFB_9_Ath_ECT9 PAMLDMLTESNRGPRASRLN---

DFCD_2_Ita_DFCD -----TSGETSRGN---------

DFC_4_Gbi_DFC1 --NLDVLTEQNRGPRTNRIRNHP

DFC_4_Gbi_DFC2 G-NLDALNEQNRGPRINRMRNPW

DFC_4_Tpl_DFC1 NWNFDVLNEQNRGPRTNRTKSPQ

DFC_4_Tpl_DFC2 G-NLDALNEQNRGPRINKTRNPW

DFC_5_Atr_DFC1 S-DFDVLNEQNRGPRTKGIS---

DFC_5_Atr_DFC2 --RIGALDELKRGLRSNRPK---

DFC_5_Nco_DFC1 G-DFDLLNEQNRGPRTNNGRNSW

DFC_5_Nco_DFC2 G-SFEKMAEQSRGPRANKIK---

DFC_6_Cka_DFC1 VCDFDLLNDQNCGPKT-STKNAS

DFC_6_Cka_DFC2 ISEFDLLNEQNRGPRTNSTKSTW

DFC_6_Cka_DFC3 IGDFDLLNEQNRGPRT---RSTW

DFC_6_Cka_DFC4 NEVFEASSEMSRGPRARRVKDPL

DFC_6_Ltu_DFC1 S-DFDLLNEQNRGPRTNSAKNTW

DFC_6_Ltu_DFC2 S-DFDLLNEQNRGPRTNSAKSSW

DFC_6_Ltu_DFC3 EGVYDSSNVLKRGPRALRASNTS

DFC_6_Ltu_DFC4 G-VFGTSHDLSRGPRASRVKDPL

DFC_7_Aam_DFC1 G-DFD-LYEQNHGPRTNSTKSSC

DFC_7_Aam_DFC2 G-DFDFLYEQNHGPRTNSSKSSC

DFC_7_Aam_DFC3 G-IFESTCDLVRGPRARRDK---

DFC_7_Dal_DFC1 V-DLDTLNEQNRGPRTNGSRSSL

DFC_7_Dal_DFC2 --DDEASTKQARGPRFNKSESYV

DFC_7_Dal_DFC3 TGDLDMLNEQNRGPRTNGIRSIL

DFC_7_Mac_DFC1 G-DFDLLNEQNRGPRTNGSRSSS

DFC_7_Mac_DFC2 G-DLDLLNEQNRGPRTNGTKSAS

DFC_7_Mac_DFC3 --TFEVSRELVHGPRANKSNSCL

DFC_7_Zma_DFC1 SVDTDLLNEQSPDSTTDALI--K

DFC_7_Zma_DFC2 G-DPNLLNEQNRGPRTNLSRNAL

DFC_7_Zma_DFC3 --NTDLLSEKNSGPRINHSKSTS

DFC_7_Zma_DFC4 --HPGEFHEIIHGPRADGRKDSS

DFC_8_Ahy_DFC1 --EFEMSPELTCGPRLKTQS---

DFC_8_Ahy_DFC2 S-NLD-SSEKERGS---------

DFC_8_Aqc_DFC1 G-DFDMLNEQNRGPRTNSTKNNV

DFC_8_Aqc_DFC2 SRSSDVLRDATRGPRSRRLHTLF

DFC_8_Cru_DFC1 S-GHDHITDGECESCSLDAQ---

DFC_8_Cru_DFC2 --ESDYLVELKCGPRA----NAK

DFC_8_Csi_DFC1 ISDFSLSNEQNHDTSTTSNKDAL

DFC_8_Csi_DFC2 AGGLGMPTELIRGPRA----ENK

DFC_8_Gra_DFC V-DFETSAELTCGPRA----LNR

DFC_8_Mes_DFC1 ISNFGSLTEQNYVPRTTNSKGSF

DFC_8_Mes_DFC2 KSDFETSTELTCGPRA----SNK

DFC_8_Mgu_DFC G-ENKAEMELTCGPRVNRANNSS

DFC_8_Mtr_DFC1 ----DSLNEQNQGPRTANAKGTL

DFC_8_Mtr_DFC2 G-ESEMSKEITRGPRF----HQR

DFC_8_Ppe_DFC1 R-DISLFNEKNHGPRTTNPKGAL

DFC_8_Ppe_DFC2 NEDFESSTELTRGPRS----RNK

DFC_8_Sly_DFC G-VFEATNELPRGPRA----NGR

DFC_9_Ath_ECT11 G-ESDYLVELKCGPRA----NAK

DFC_9_Ath_ECT8 DLANGHITNGECESCSLDAE---

DFD_4_Gbi_DFD G-NLDVLSEQNRGPRTNRTRNQW

DFD_5_Atr_DFD G-TSDALIEQNRGPRINRVGNQV

DFD_5_Nco_DFD R-SPDALSEQNRGPRTSRSKNQW

DFD_6_Cka_DFD1 G-SPDVFGEQNRGPRNNRLKSSW

DFD_6_Cka_DFD2 G-NPDVLGEQNRGPRTNRLKSPL

DFD_6_Ltu_DFD G-SPDVLGEQNRGPRTNRLKSPW

DFD_7_Aam_DFD A-SLGMLSEQNRVPRTNRSKGQW

DFD_7_Dal_DFD R-SSRMLYDQSRAPRANRSKVQQ

DFD_7_Mac_DFD1 GRHPYVLGEPNKESK-NRLKDQS

DFD_7_Mac_DFD2 GRNPDVLAEQNKGPRTNRPKDQF

DFD_7_Zma_DFD S-CPIVLGEQNRGPRSGKSGGKL

DFD_8_Ahy_DFD G-NLDQLGEQNRGPRINKSKNQF

DFD_8_Aqc_DFD G-NPDFLSEQNRGPRTNRSRGQW

DFD_8_Cru_DFD1 D-LTRDTTEQYRGPRTRRSRNQL

DFD_8_Cru_DFD2 NIIPDRVGEQNRGRRSRGLGNQL

DFD_8_Csi_DFD GRNPDMFSEQNRGPRTSRSKDQL

DFD_8_Egr_DFD SGPLDAQGEQNRGPRTNRSKSQL

DFD_8_Gra_DFD1 G-SLDSWAEQNRGPRTNRSKNQL

DFD_8_Gra_DFD2 G-YPDTLGEQNRGPRTNRLKNQF

DFD_8_Mes_DFD G-GPDSLGEQNRGPRTNKSKNQL

DFD_8_Mgu_DFD1 RASPDALREQNGGPRTNKLKNEL

DFD_8_Mgu_DFD2 RASPDTLREQNGGPRTNKLKNEL

DFD_8_Mgu_DFD3 RASPDTLSEQNGGPRTNKLKNEL

DFD_8_Mtr_DFD1 DVNRSLLGEQNRGPRISRPKHHL

DFD_8_Mtr_DFD2 G-NVSVLGDQNRGPRTSRSKHQL

DFD_8_Ppe_DFD G-GLEALGEQNCGPRINRLKNQI

DFD_8_Sly_DFD V-SPDMLMEQNRGPRIDKMKKQL

DFD_9_Ath_ECT6 N-TTGYTSEQNRGSRTRRSRNQL

DFD_9_Ath_ECT7 NIIPDTVREQNRGRRSRALGNQL
