# Supplementary figures and images for: ALBA proteins facilitate cytoplasmic YTHDF-mediated reading of m6A in Arabidopsis
Source: EMBO J. 2024 Nov 29;43(24):6626–55. doi: 10.1038/s44318-024-00312-0 (PMC11649824; doi:10.1038/s44318-024-00312-0)

Source data for Figure 1H

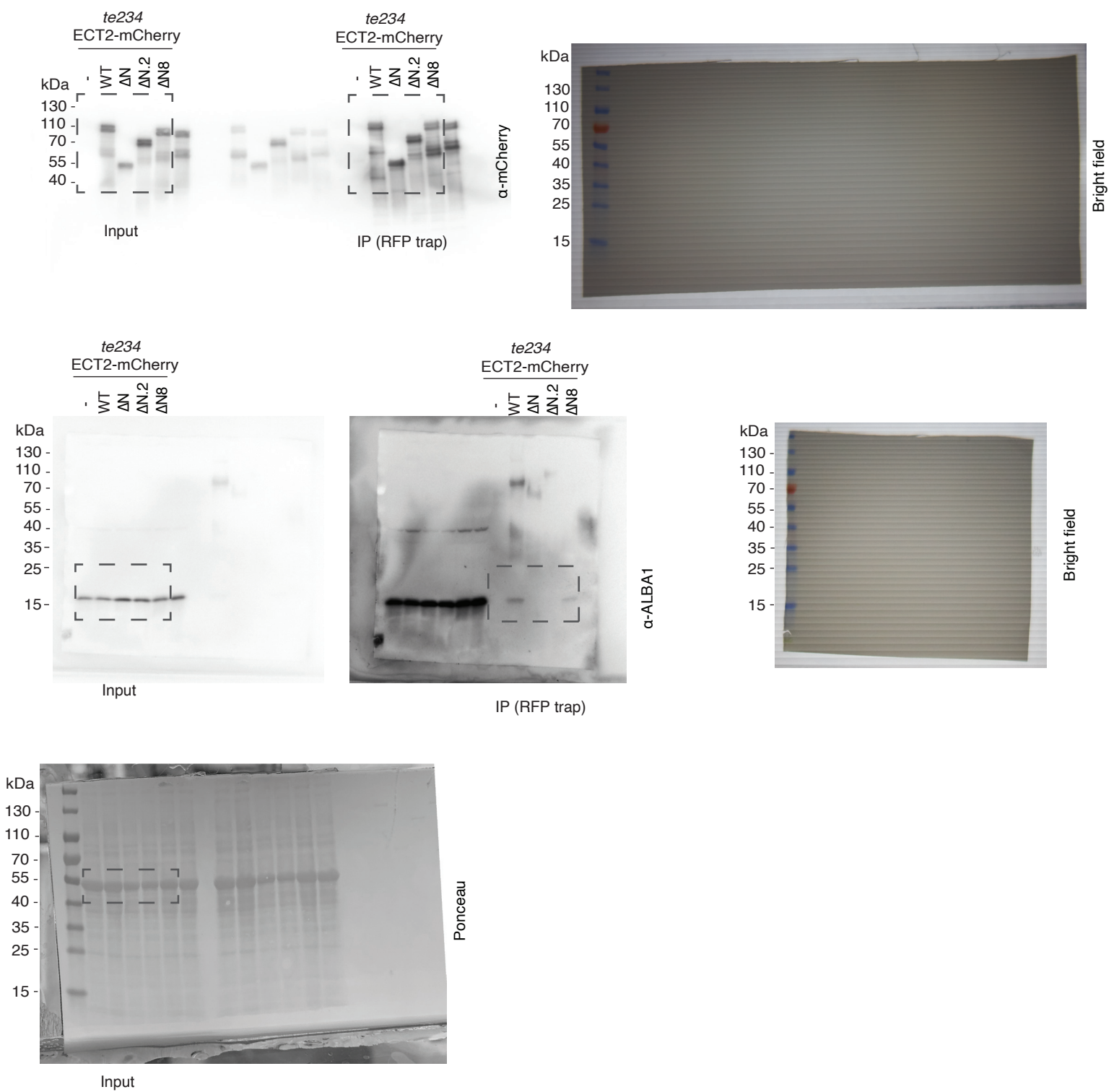

Supplement: Supplementary file 10 — Source data Fig. 1 [file 44318_2024_312_MOESM10_ESM.zip › Source data for Fig 1/1H/mCherry CoIP-WB.pdf]

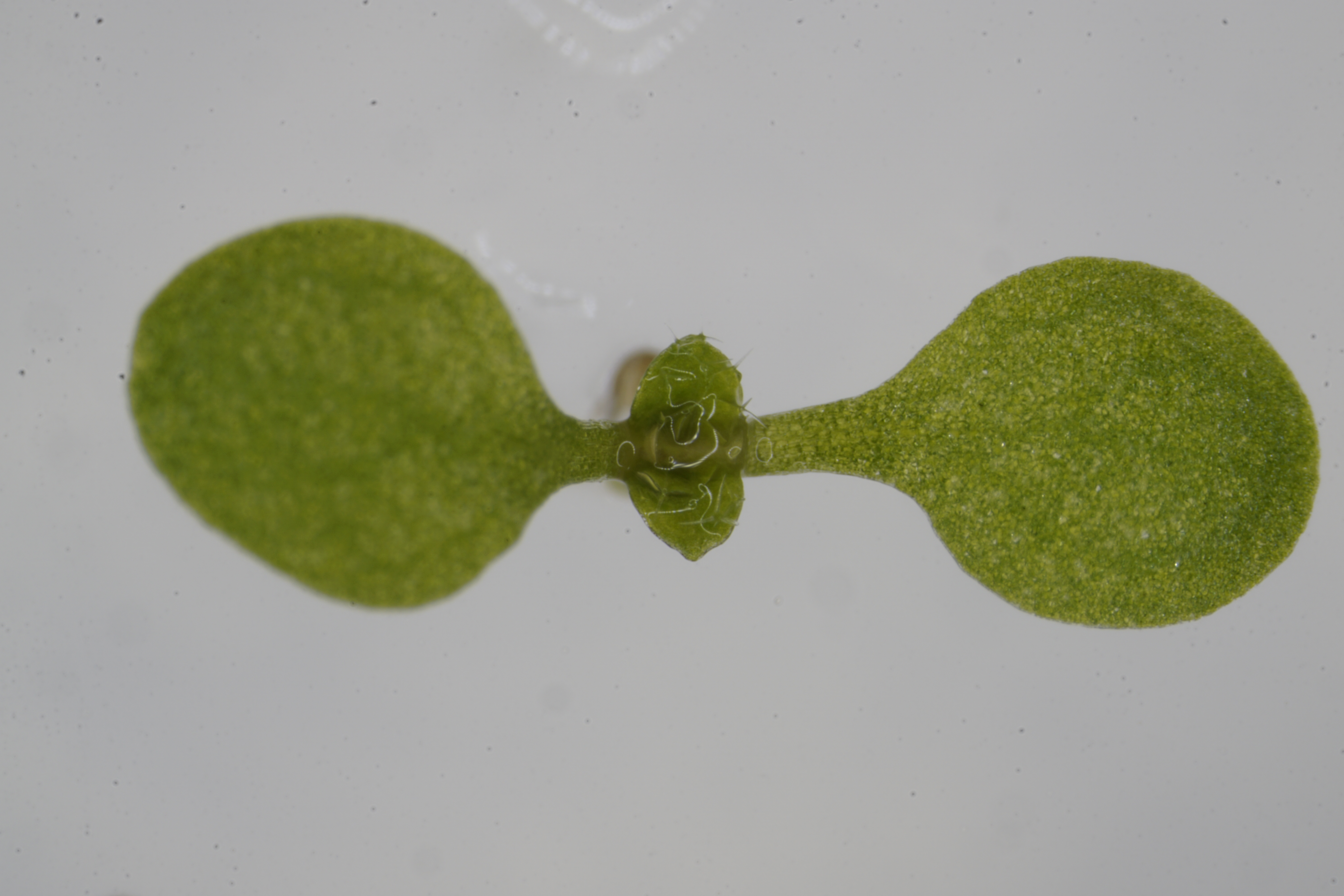

Supplement: Supplementary file 10 — Source data Fig. 1 [file 44318_2024_312_MOESM10_ESM.zip › Source data for Fig 1/1B/de34 ect2-5.JPG]

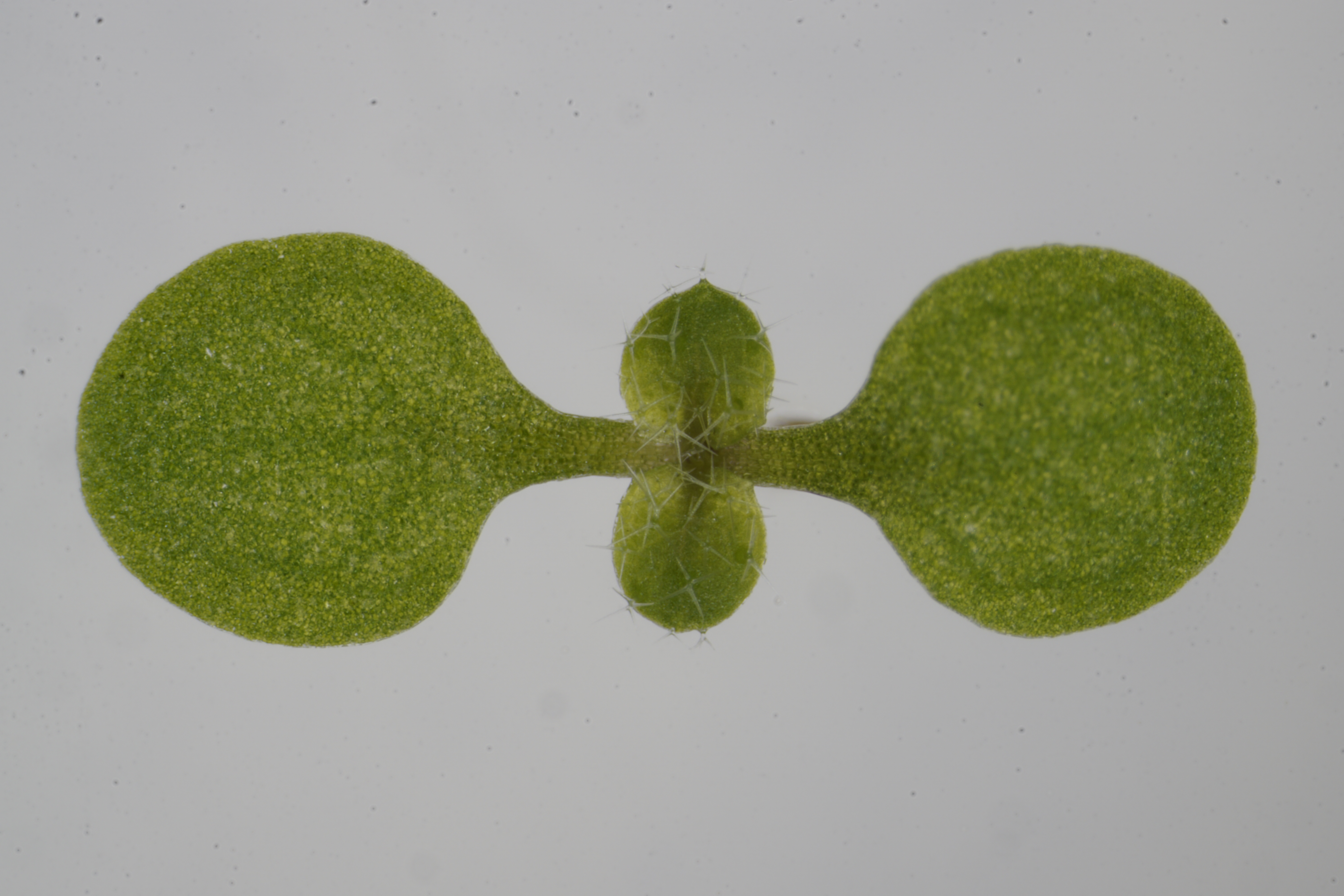

Supplement: Supplementary file 10 — Source data Fig. 1 [file 44318_2024_312_MOESM10_ESM.zip › Source data for Fig 1/1B/de34.JPG]

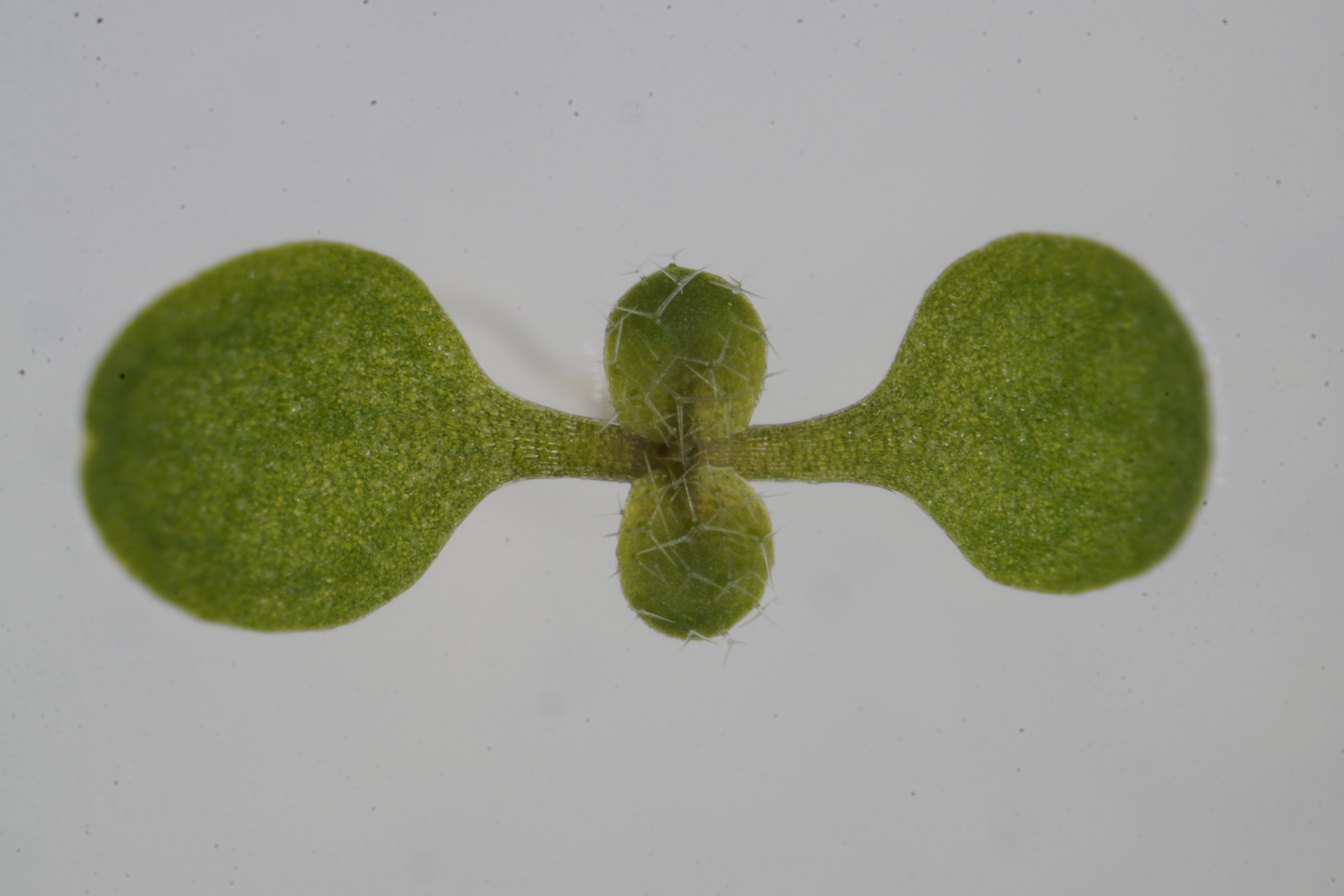

Supplement: Supplementary file 10 — Source data Fig. 1 [file 44318_2024_312_MOESM10_ESM.zip › Source data for Fig 1/1B/Col-0 WT.JPG]

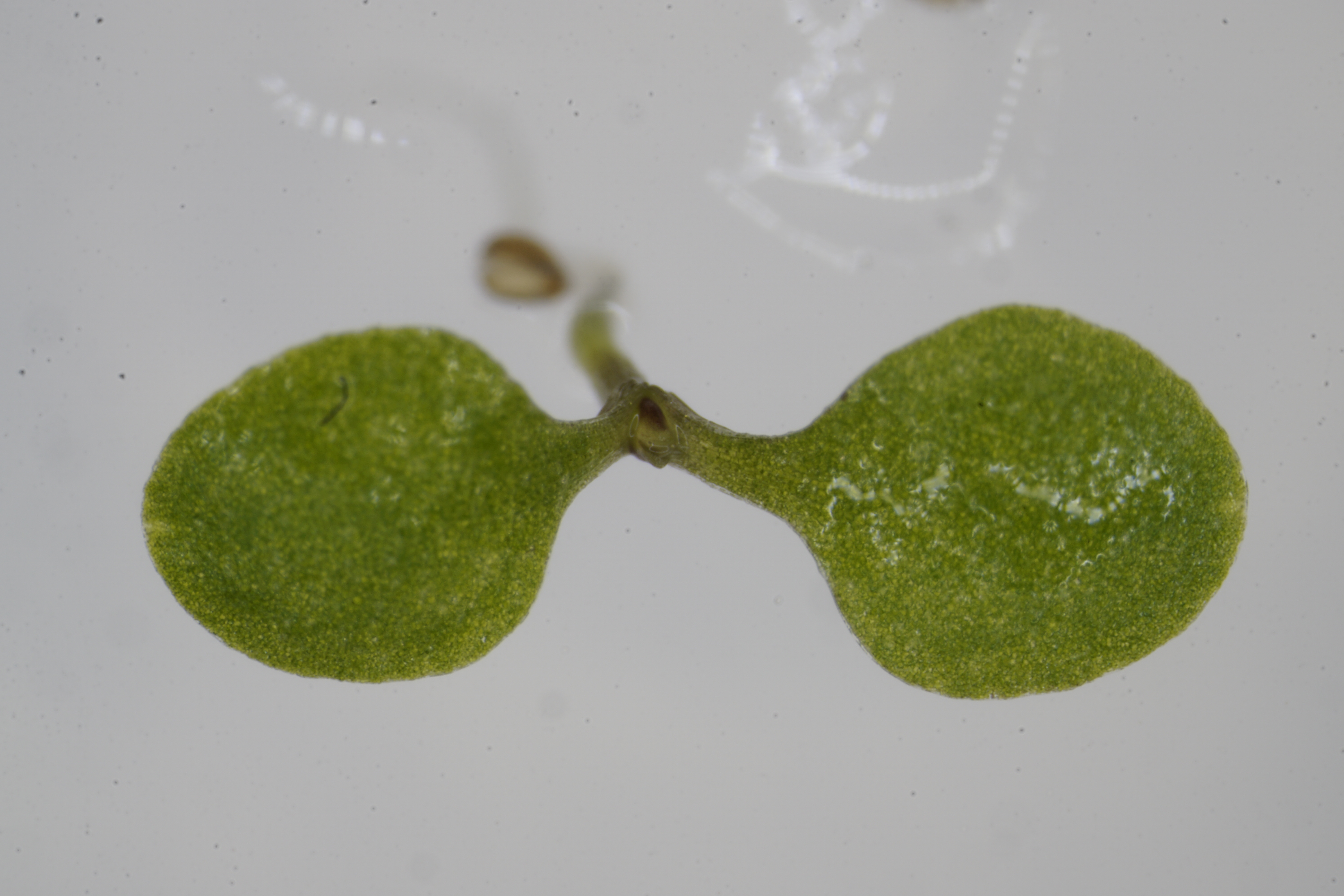

Supplement: Supplementary file 10 — Source data Fig. 1 [file 44318_2024_312_MOESM10_ESM.zip › Source data for Fig 1/1B/te234.JPG]

Source data for Figure 1E

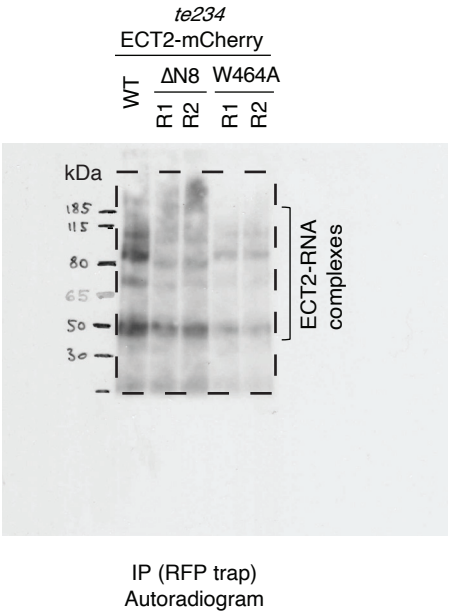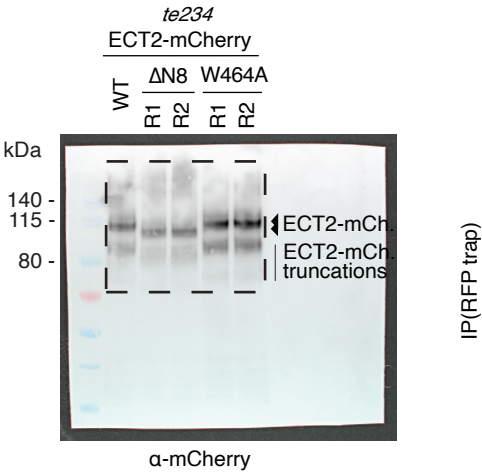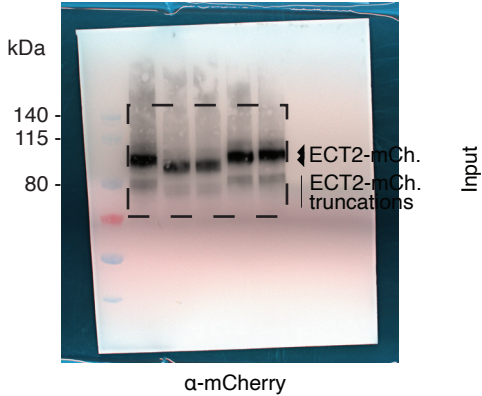

Supplement: Supplementary file 10 — Source data Fig. 1 [file 44318_2024_312_MOESM10_ESM.zip › Source data for Fig 1/1E/Autoradiogram and western from UV-Xlink and Co-IP.pdf]

### Source data for Figure 1D

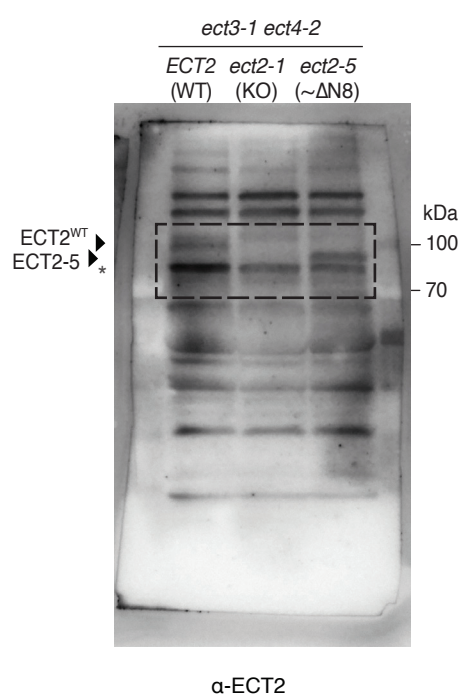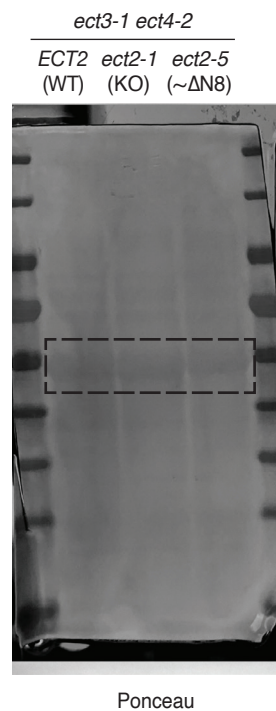

Supplement: Supplementary file 10 — Source data Fig. 1 [file 44318_2024_312_MOESM10_ESM.zip › Source data for Fig 1/1D/ECT2 western and ponceau.pdf]

Source data for Figure 2G

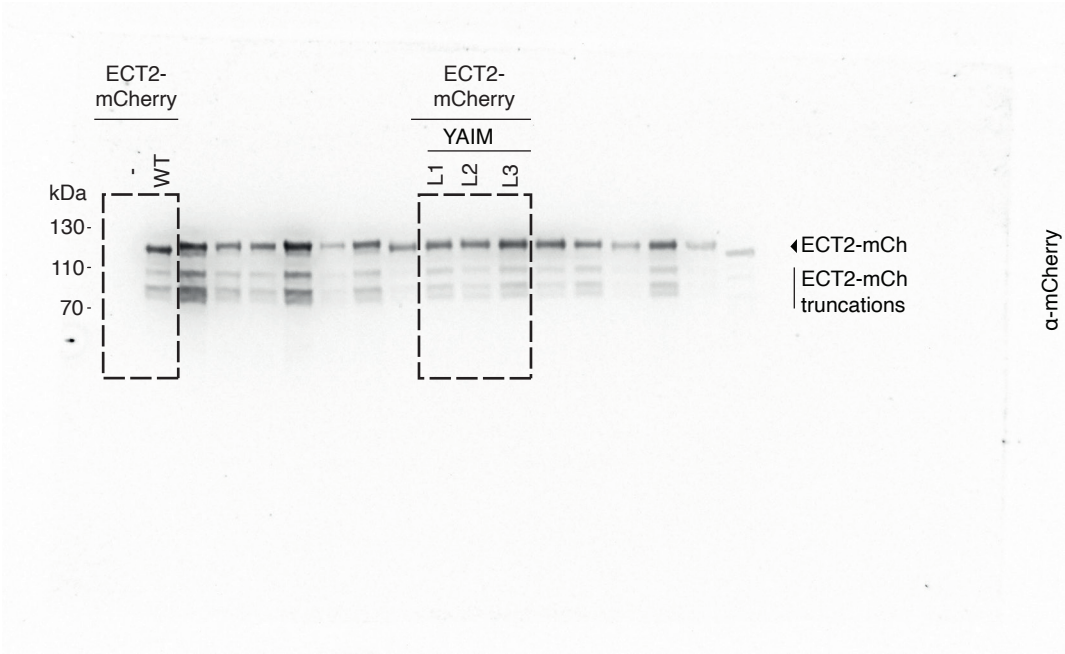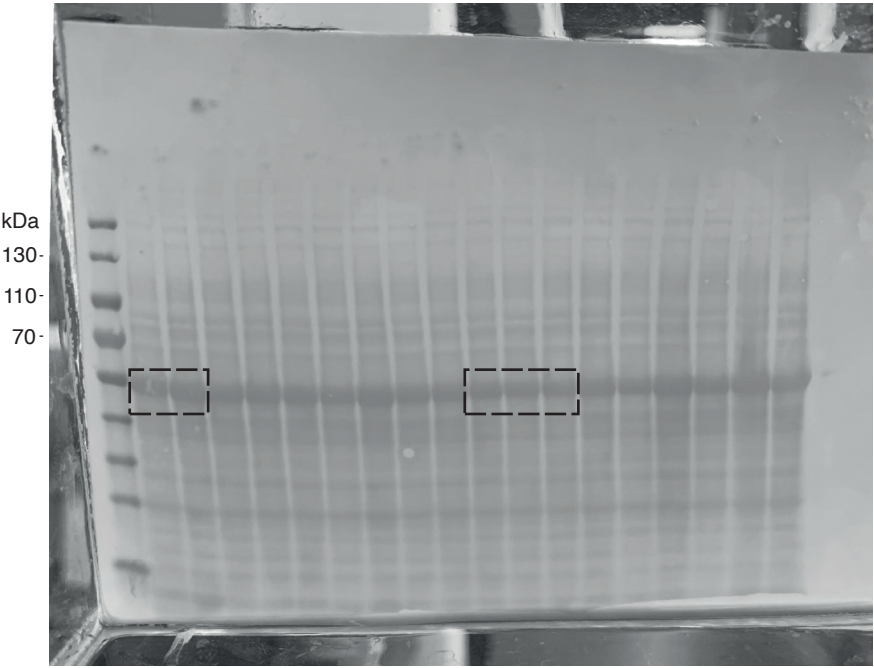

Supplement: Supplementary file 11 — Source data Fig. 2 [file 44318_2024_312_MOESM11_ESM.zip › Source data for Fig 2/2G/anti-mCherry western.pdf]

Source data for Figure 2H

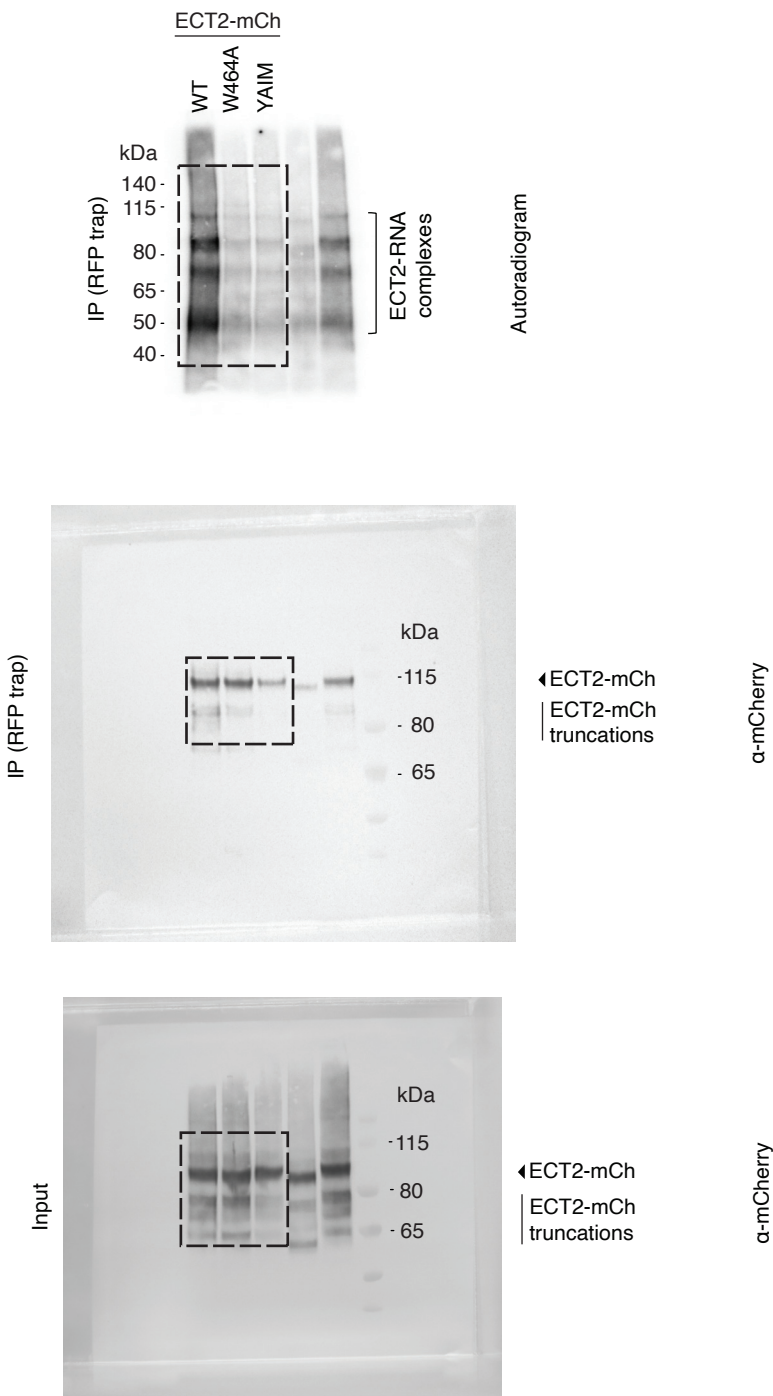

Supplement: Supplementary file 11 — Source data Fig. 2 [file 44318_2024_312_MOESM11_ESM.zip › Source data for Fig 2/2H/Autoradiogram and western after UV-Xlink and CoIP.pdf]

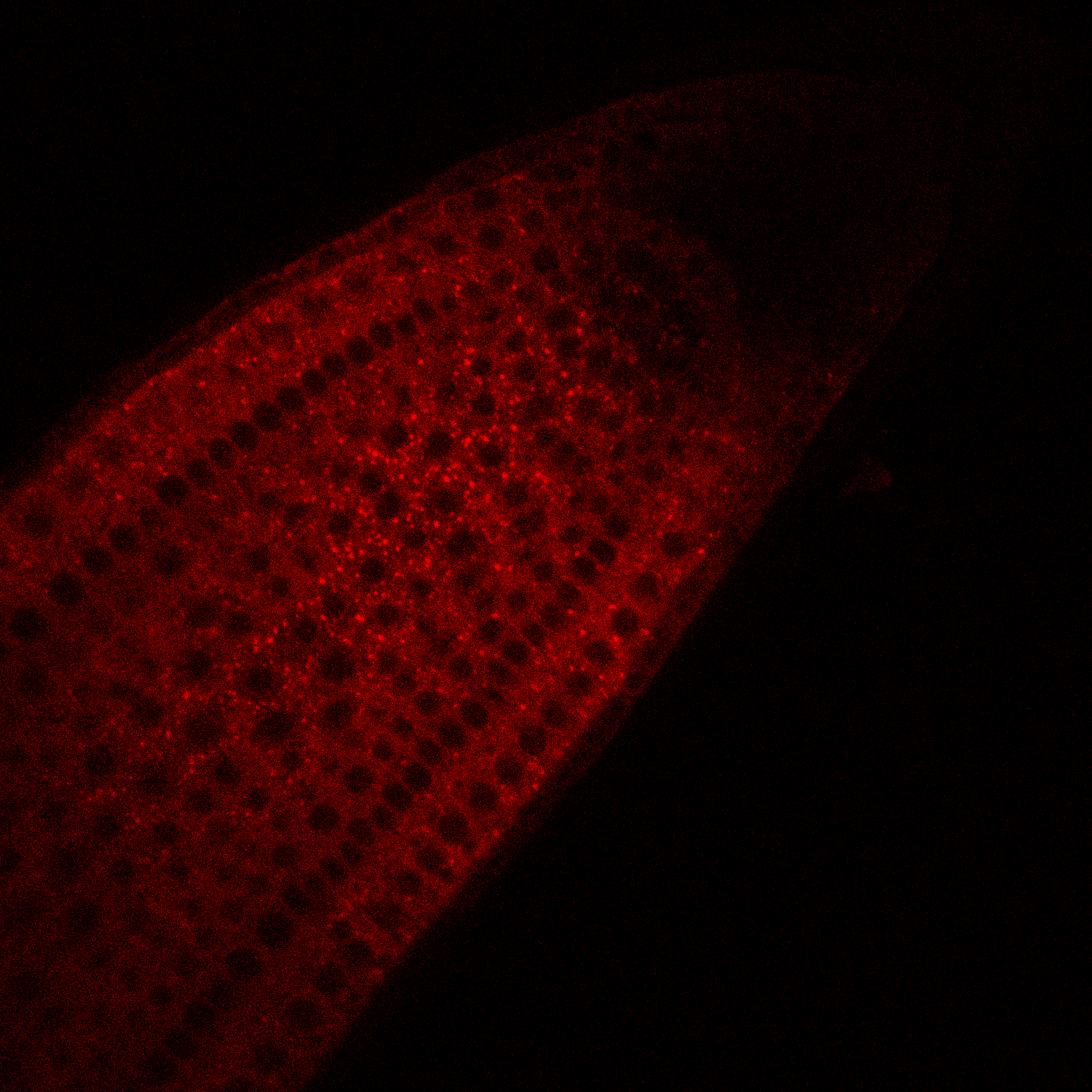

Supplement: Supplementary file 12 — Source data Fig. 3 [file 44318_2024_312_MOESM12_ESM.zip › Source data for Fig 3/3B/ECT2-mCh+ALBA1-TFP - mCherry.tif]

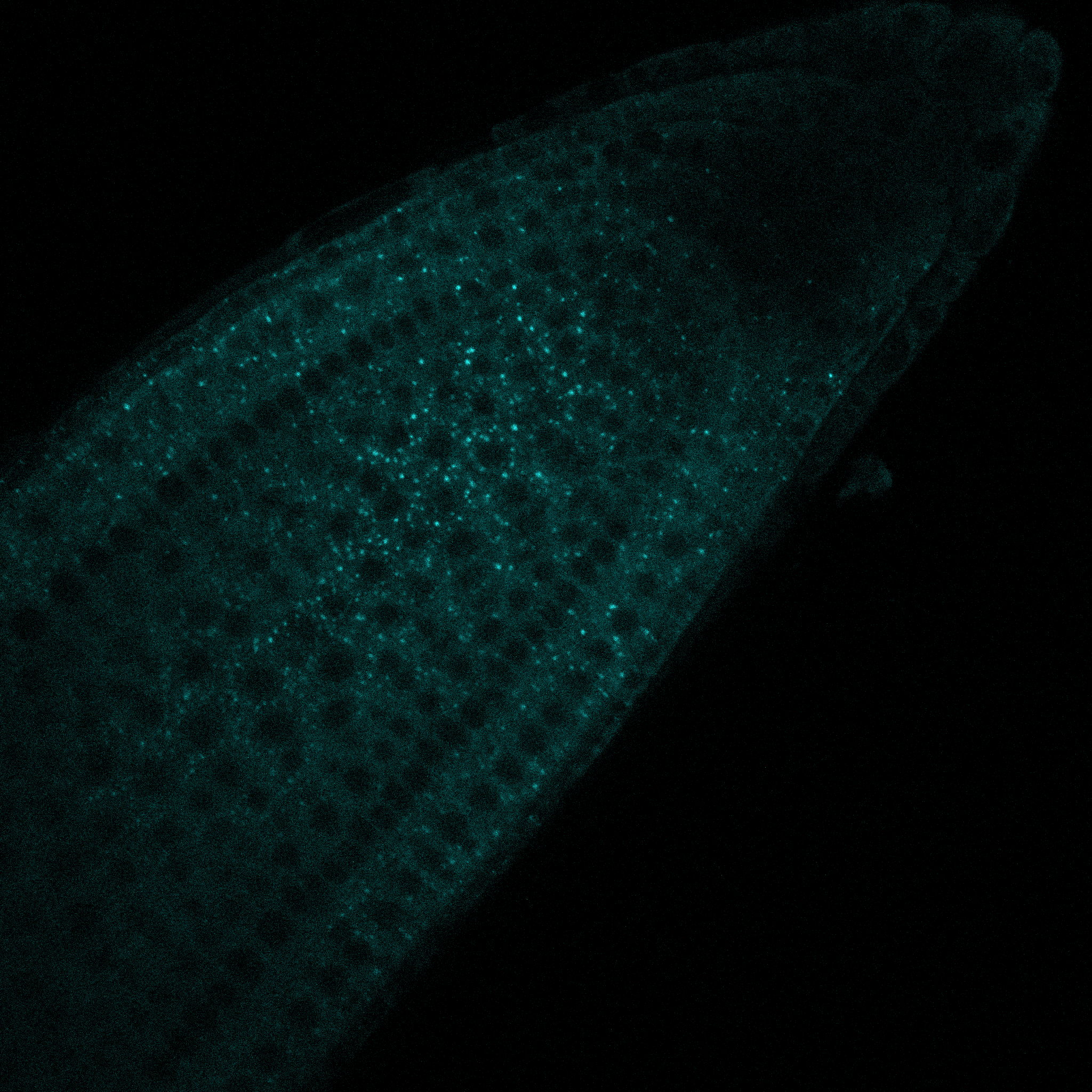

Supplement: Supplementary file 12 — Source data Fig. 3 [file 44318_2024_312_MOESM12_ESM.zip › Source data for Fig 3/3B/ECT2-mCh+ALBA1-TFP - TFP.tif]

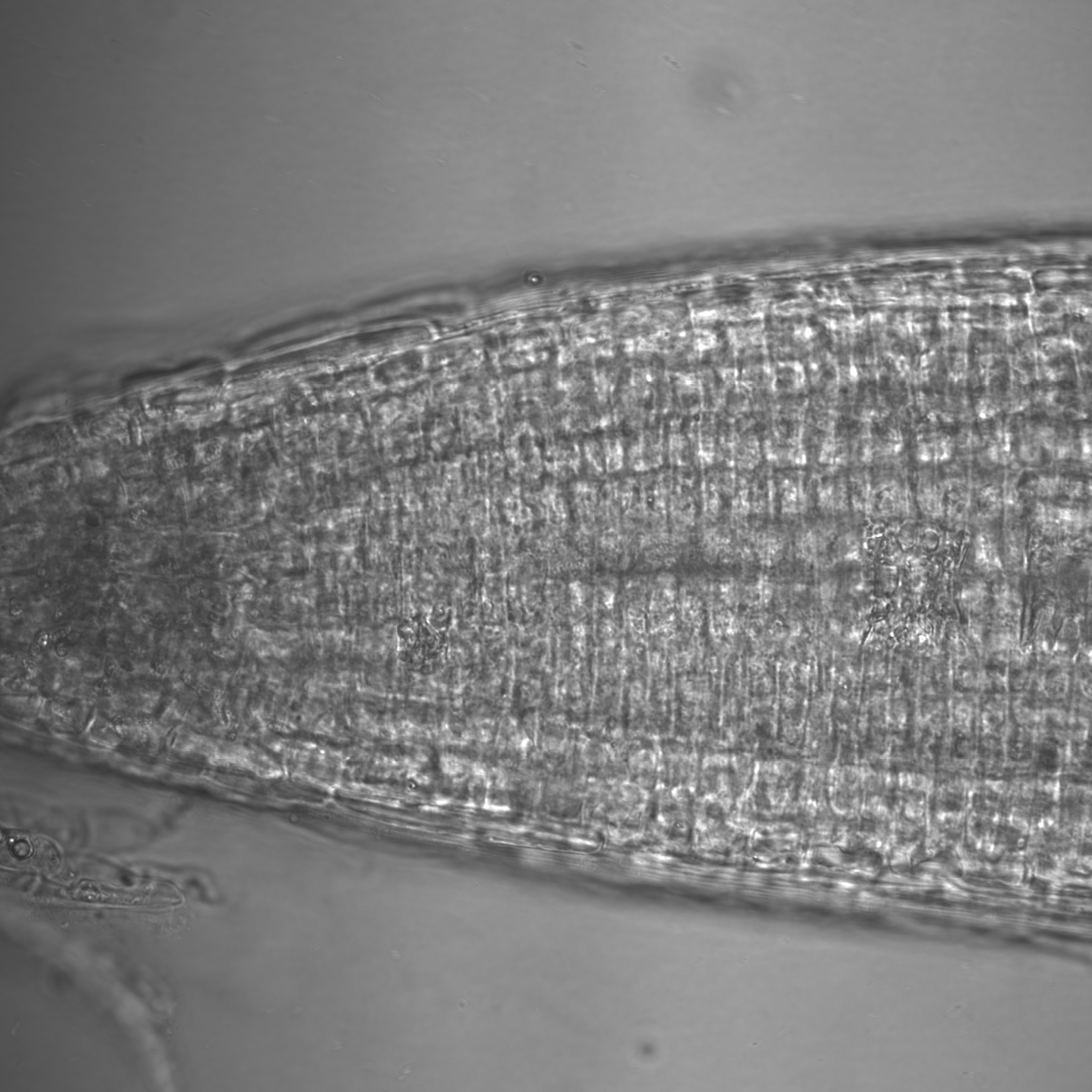

Supplement: Supplementary file 12 — Source data Fig. 3 [file 44318_2024_312_MOESM12_ESM.zip › Source data for Fig 3/3B/ECT2-mCh+ALBA4-Venus - bright field.tif]

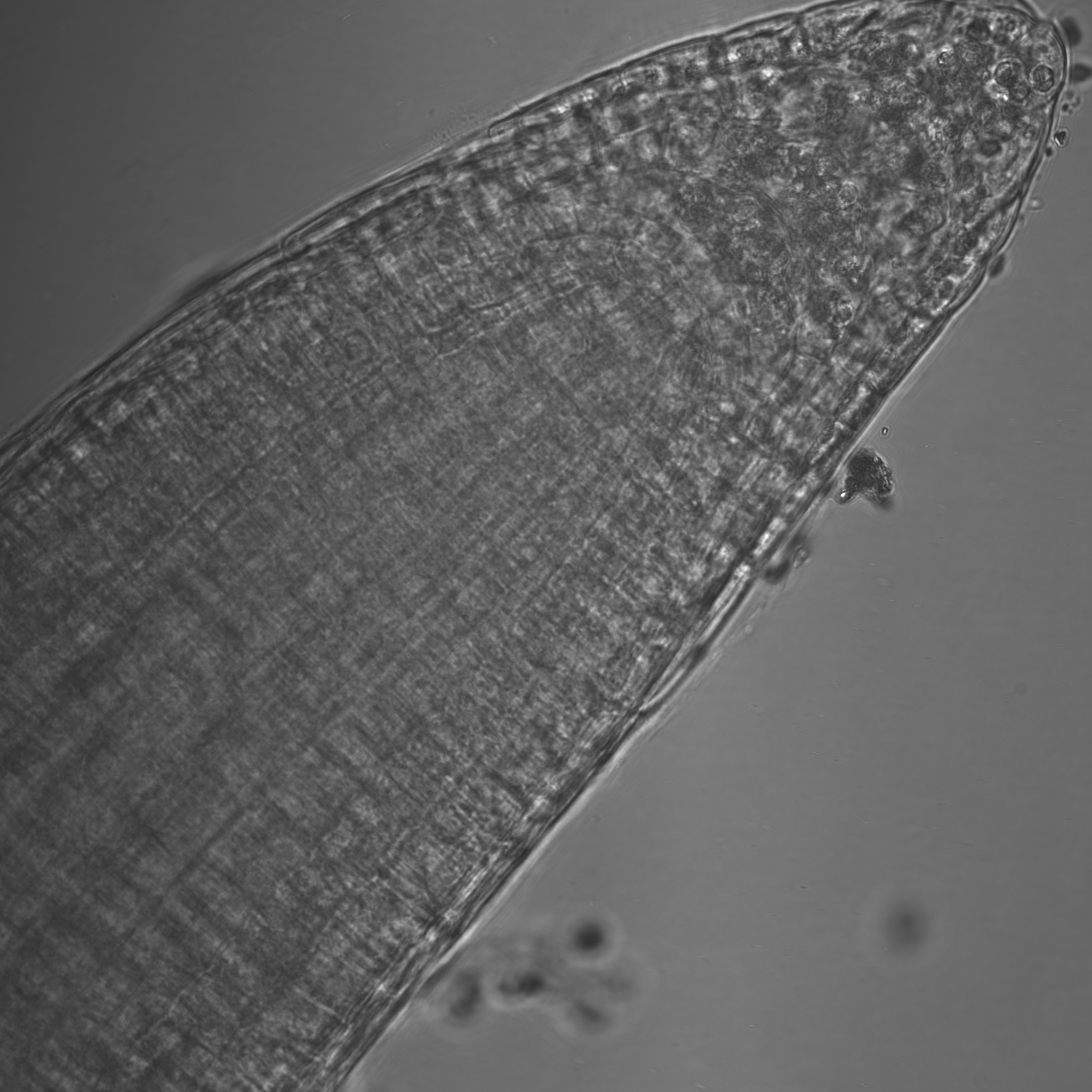

Supplement: Supplementary file 12 — Source data Fig. 3 [file 44318_2024_312_MOESM12_ESM.zip › Source data for Fig 3/3B/ECT2-mCh+ALBA1-TFP - bright field.tiff]

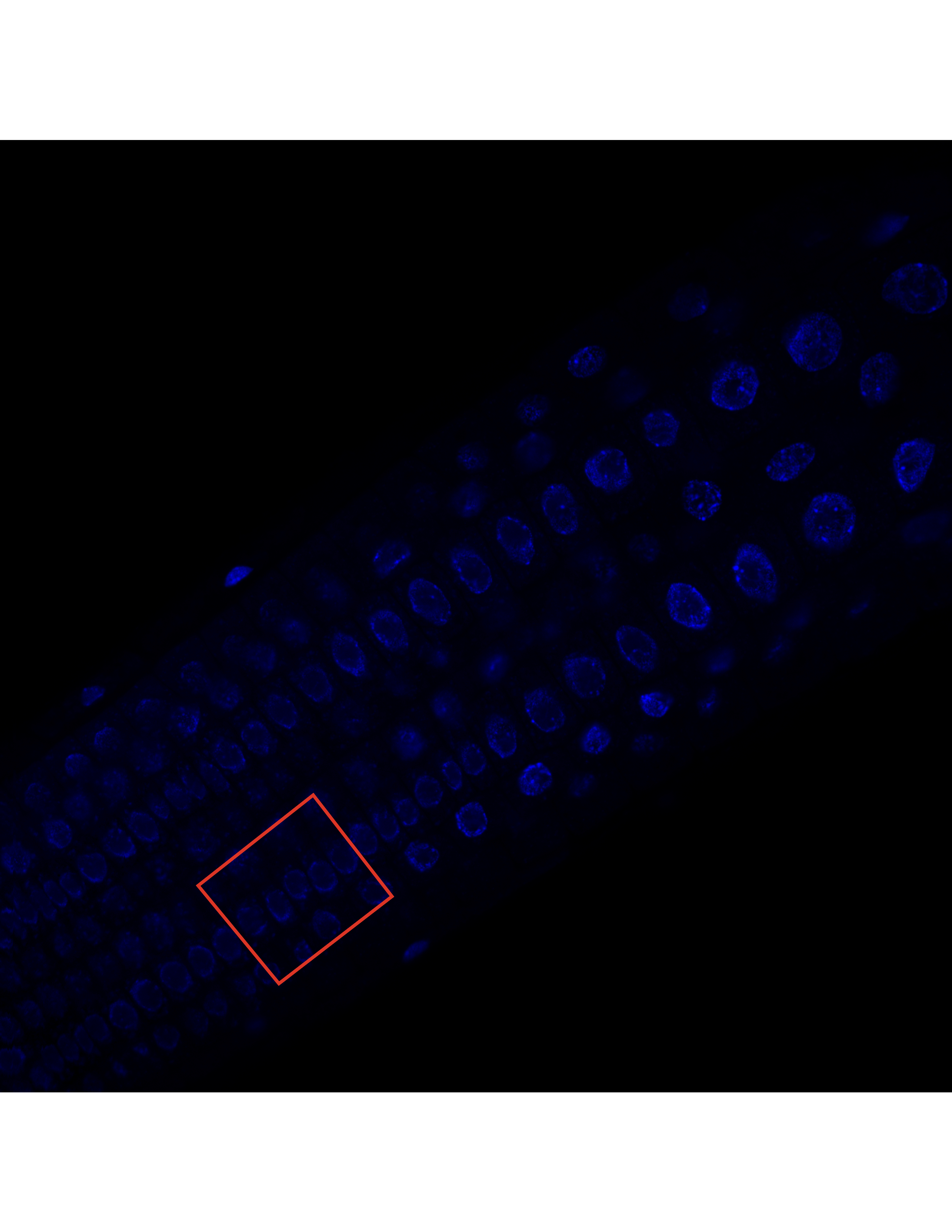

Supplement: Supplementary file 12 — Source data Fig. 3 [file 44318_2024_312_MOESM12_ESM.zip › Source data for Fig 3/3C/ALBA5-GFP/ALBA5 DAPI.jpg]

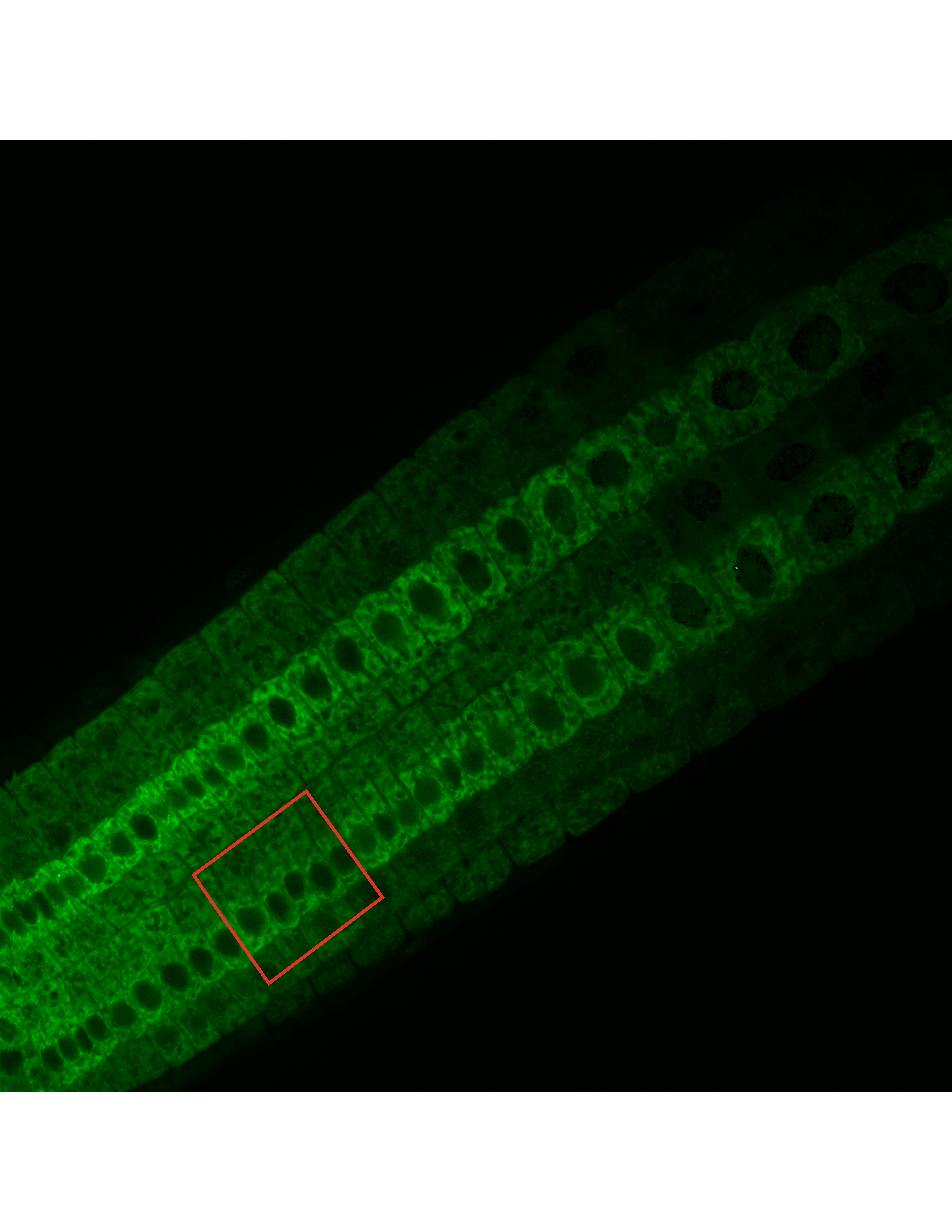

Supplement: Supplementary file 12 — Source data Fig. 3 [file 44318_2024_312_MOESM12_ESM.zip › Source data for Fig 3/3C/ALBA5-GFP/ALBA5 GFP.jpg]

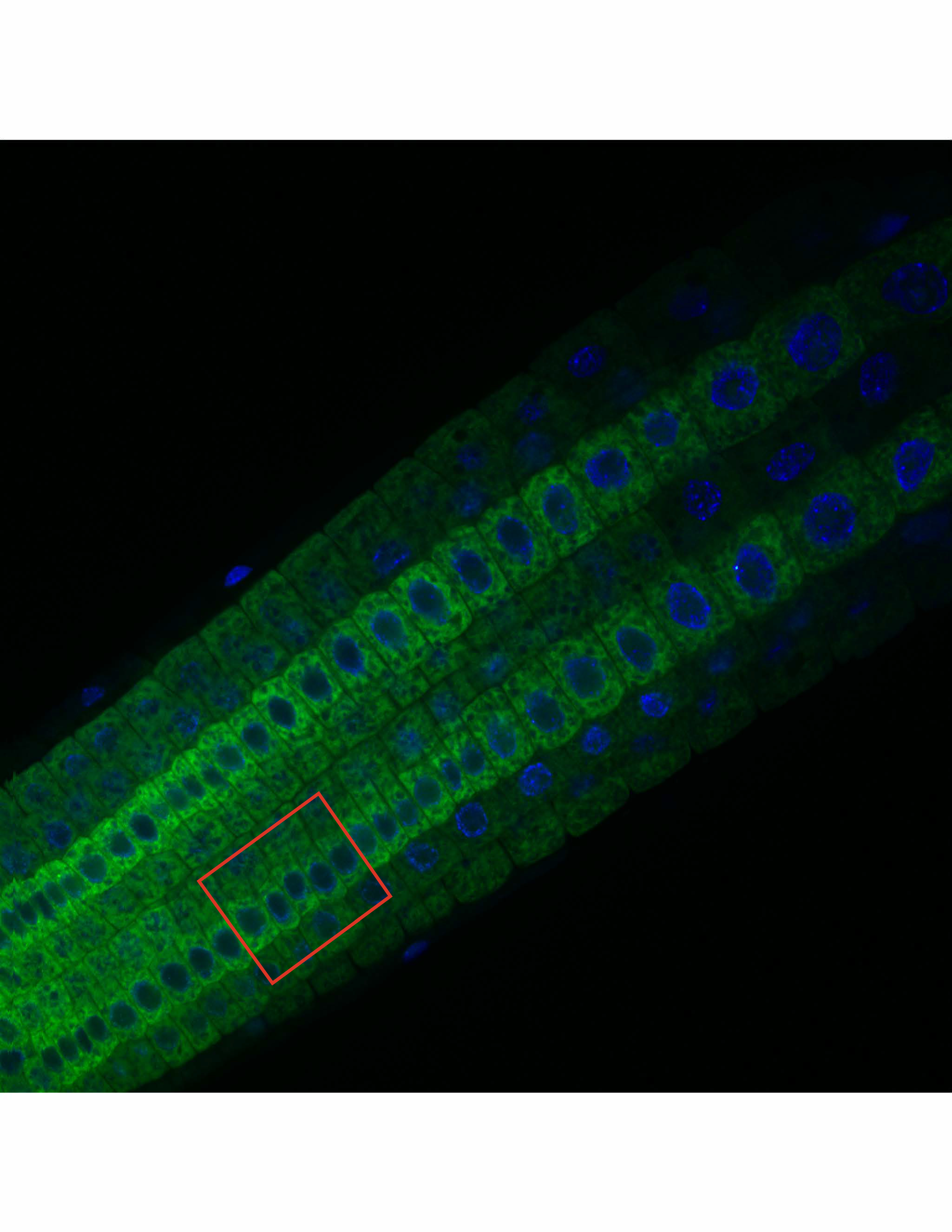

Supplement: Supplementary file 12 — Source data Fig. 3 [file 44318_2024_312_MOESM12_ESM.zip › Source data for Fig 3/3C/ALBA5-GFP/ALBA5 merge.jpg]

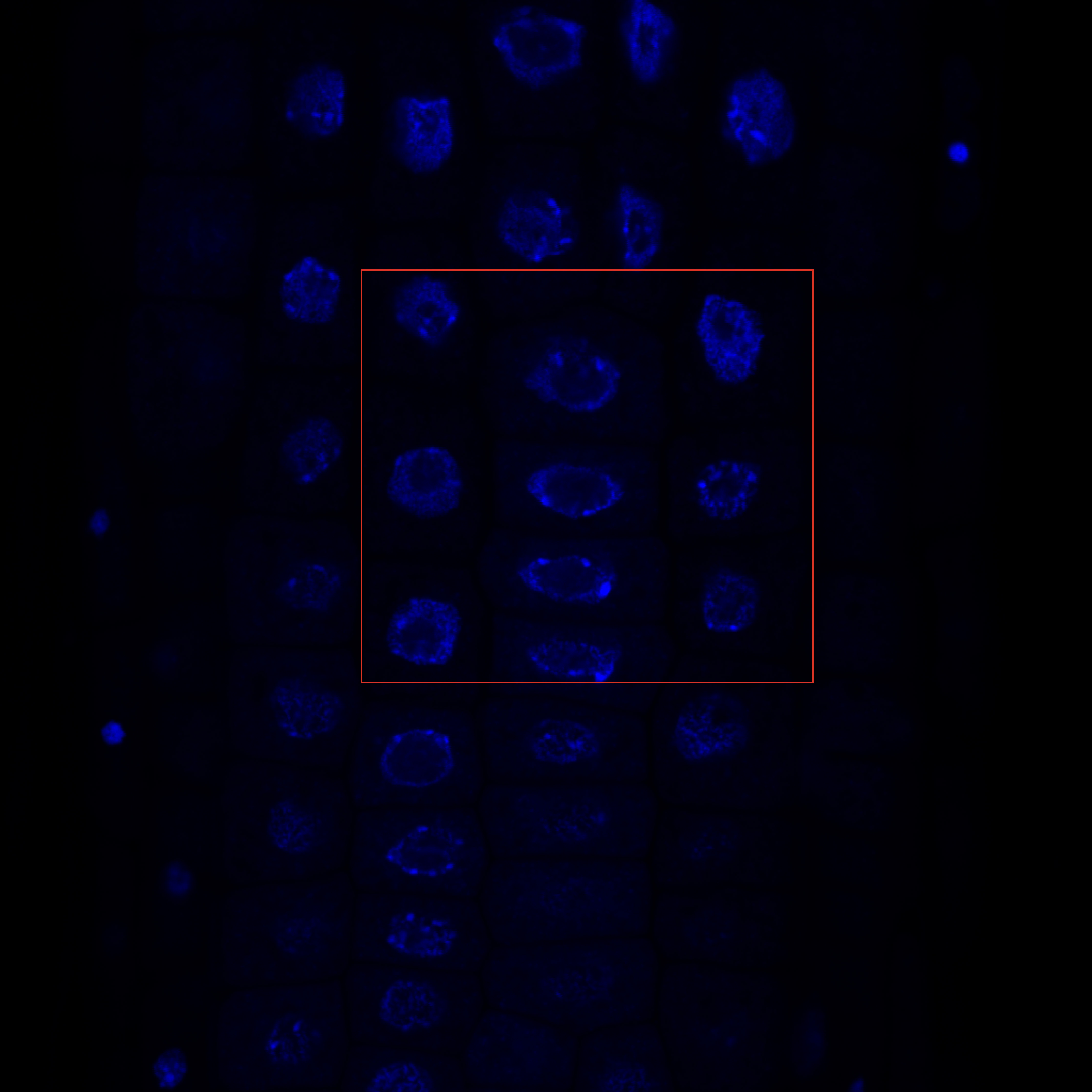

Supplement: Supplementary file 12 — Source data Fig. 3 [file 44318_2024_312_MOESM12_ESM.zip › Source data for Fig 3/3C/ALBA2-GFP/ALBA2 DAPI.jpg]

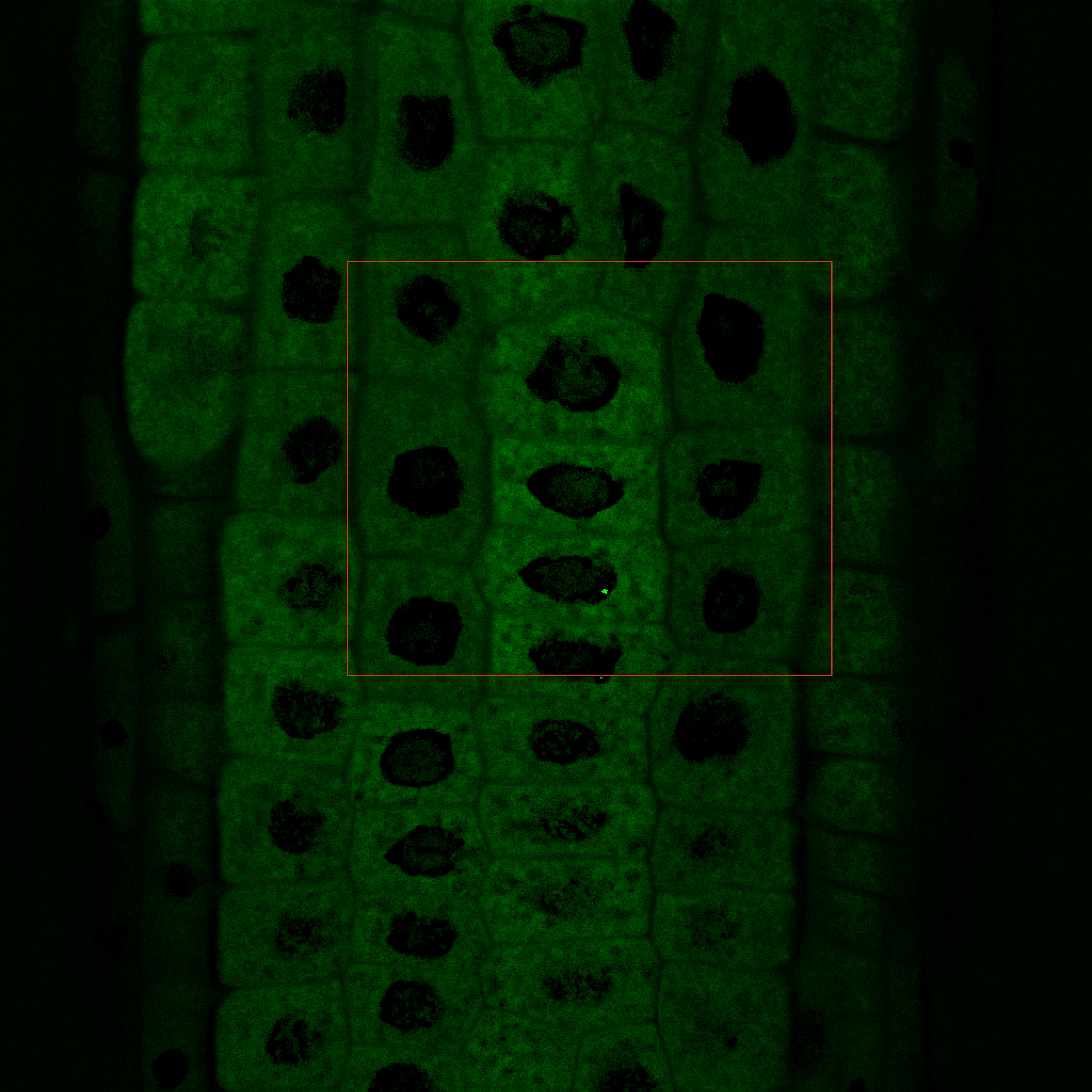

Supplement: Supplementary file 12 — Source data Fig. 3 [file 44318_2024_312_MOESM12_ESM.zip › Source data for Fig 3/3C/ALBA2-GFP/ALBA2 GFP.jpg]

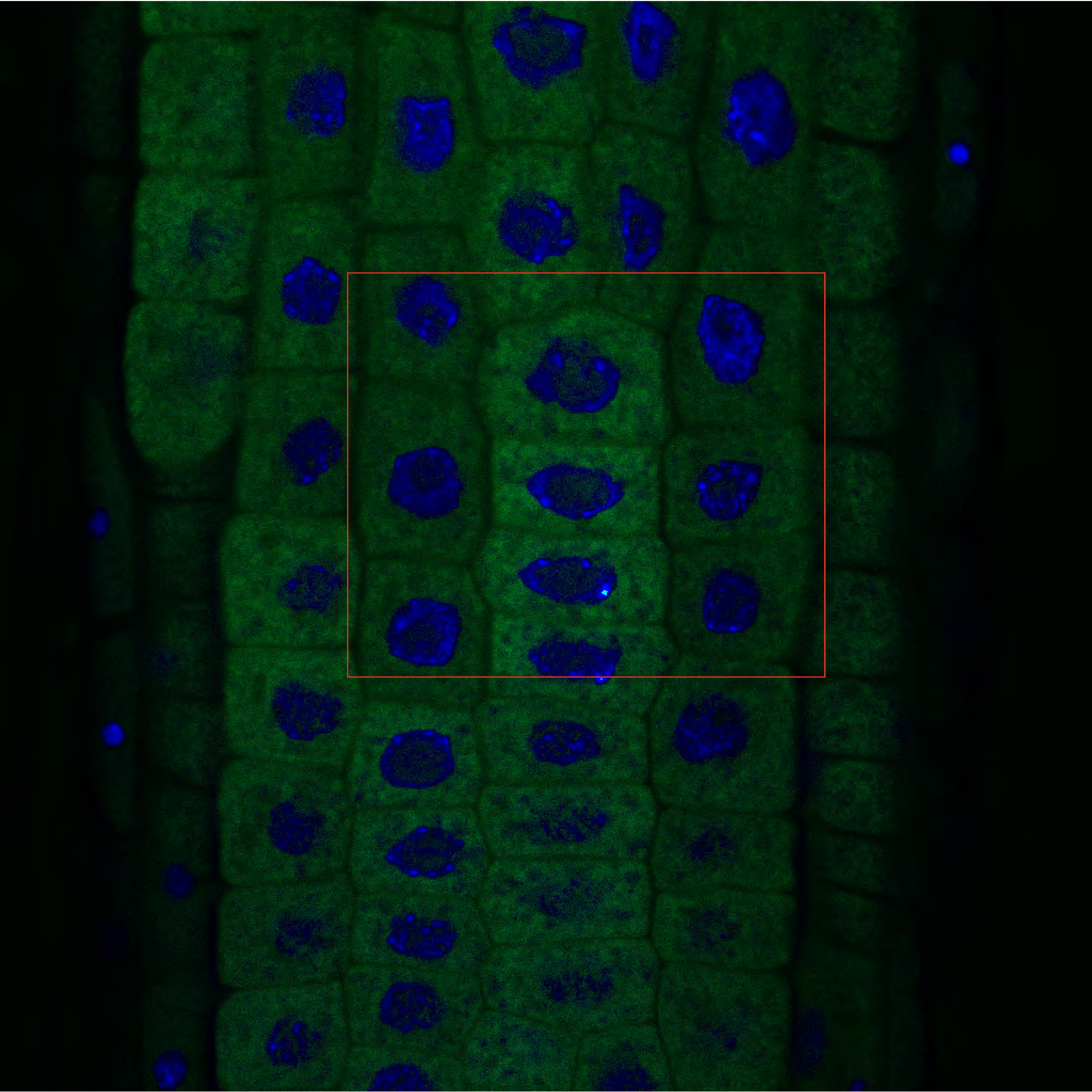

Supplement: Supplementary file 12 — Source data Fig. 3 [file 44318_2024_312_MOESM12_ESM.zip › Source data for Fig 3/3C/ALBA2-GFP/ALBA2 merge.jpg]

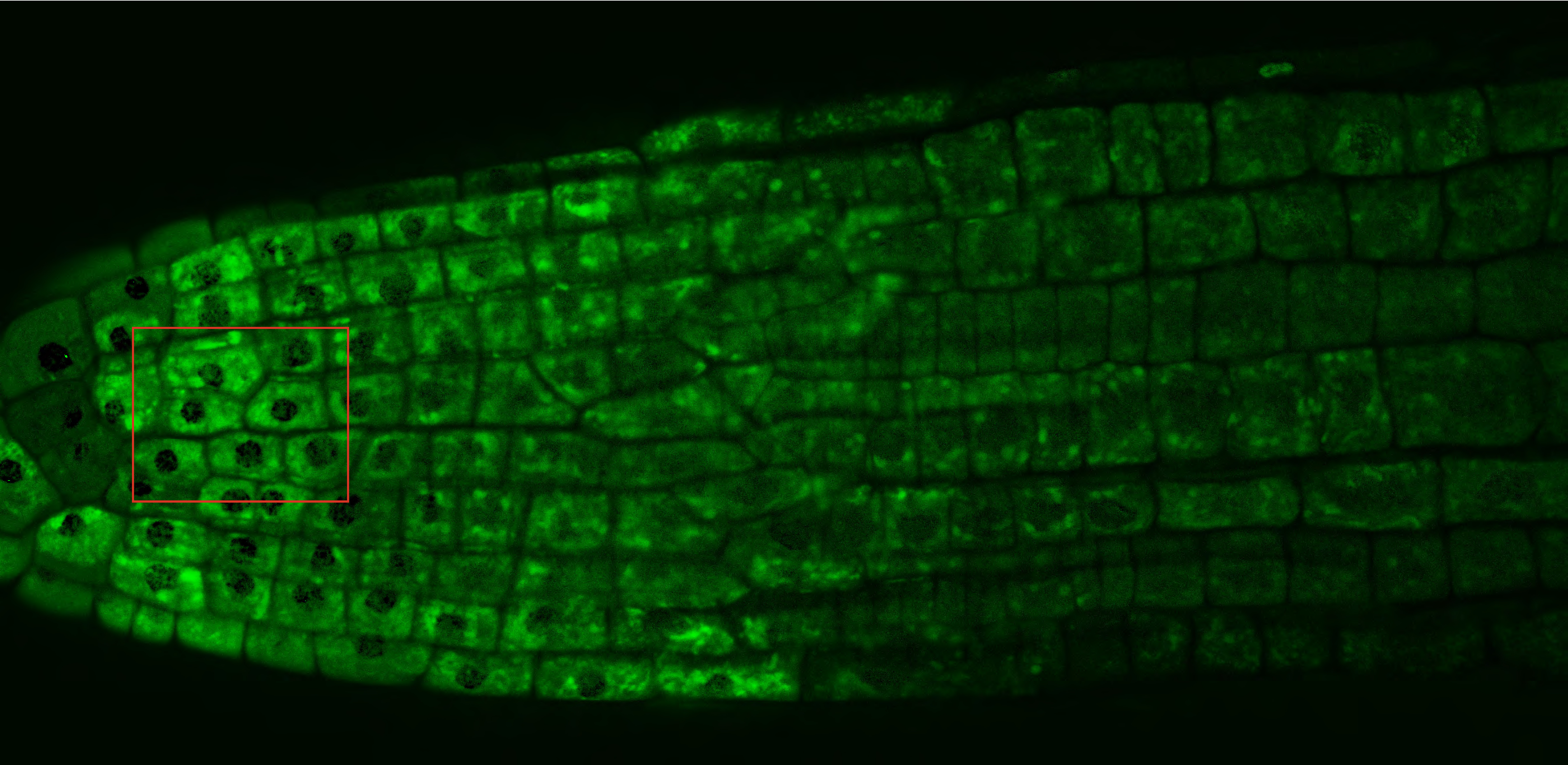

Supplement: Supplementary file 12 — Source data Fig. 3 [file 44318_2024_312_MOESM12_ESM.zip › Source data for Fig 3/3C/35S-GFP/35S GFP.jpg]

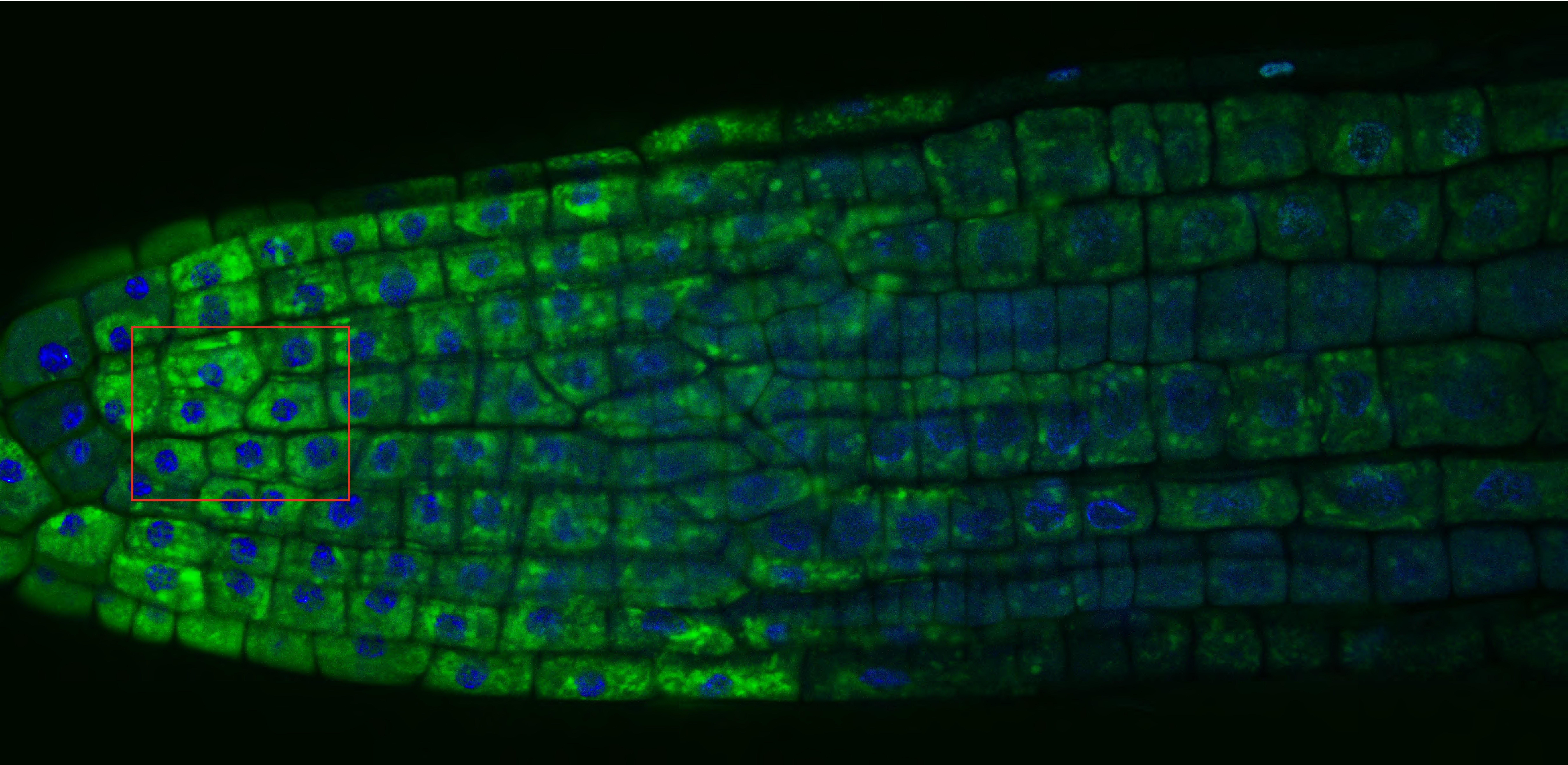

Supplement: Supplementary file 12 — Source data Fig. 3 [file 44318_2024_312_MOESM12_ESM.zip › Source data for Fig 3/3C/35S-GFP/35S merge.jpg]

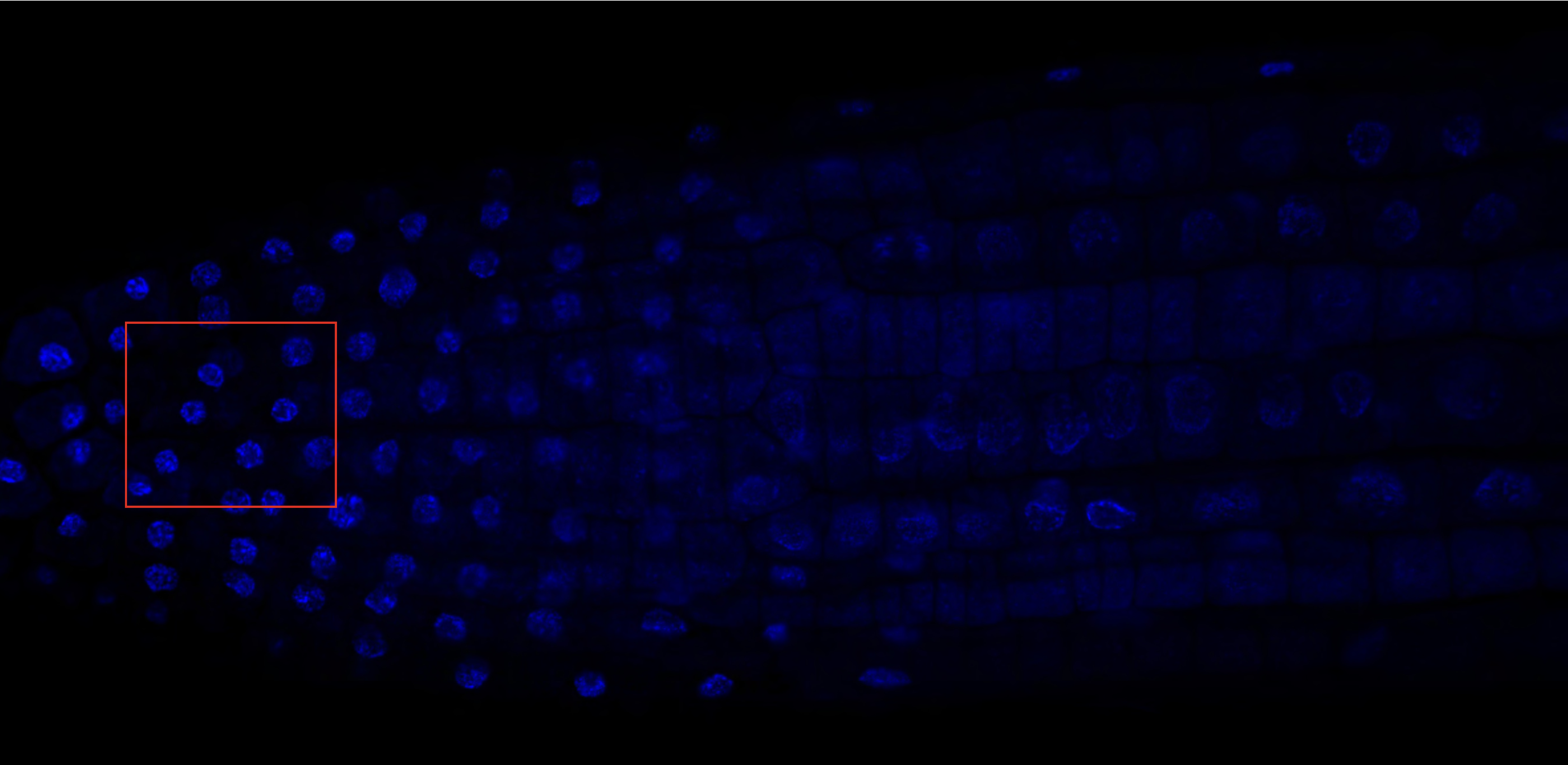

Supplement: Supplementary file 12 — Source data Fig. 3 [file 44318_2024_312_MOESM12_ESM.zip › Source data for Fig 3/3C/35S-GFP/35S DAPI.jpg]

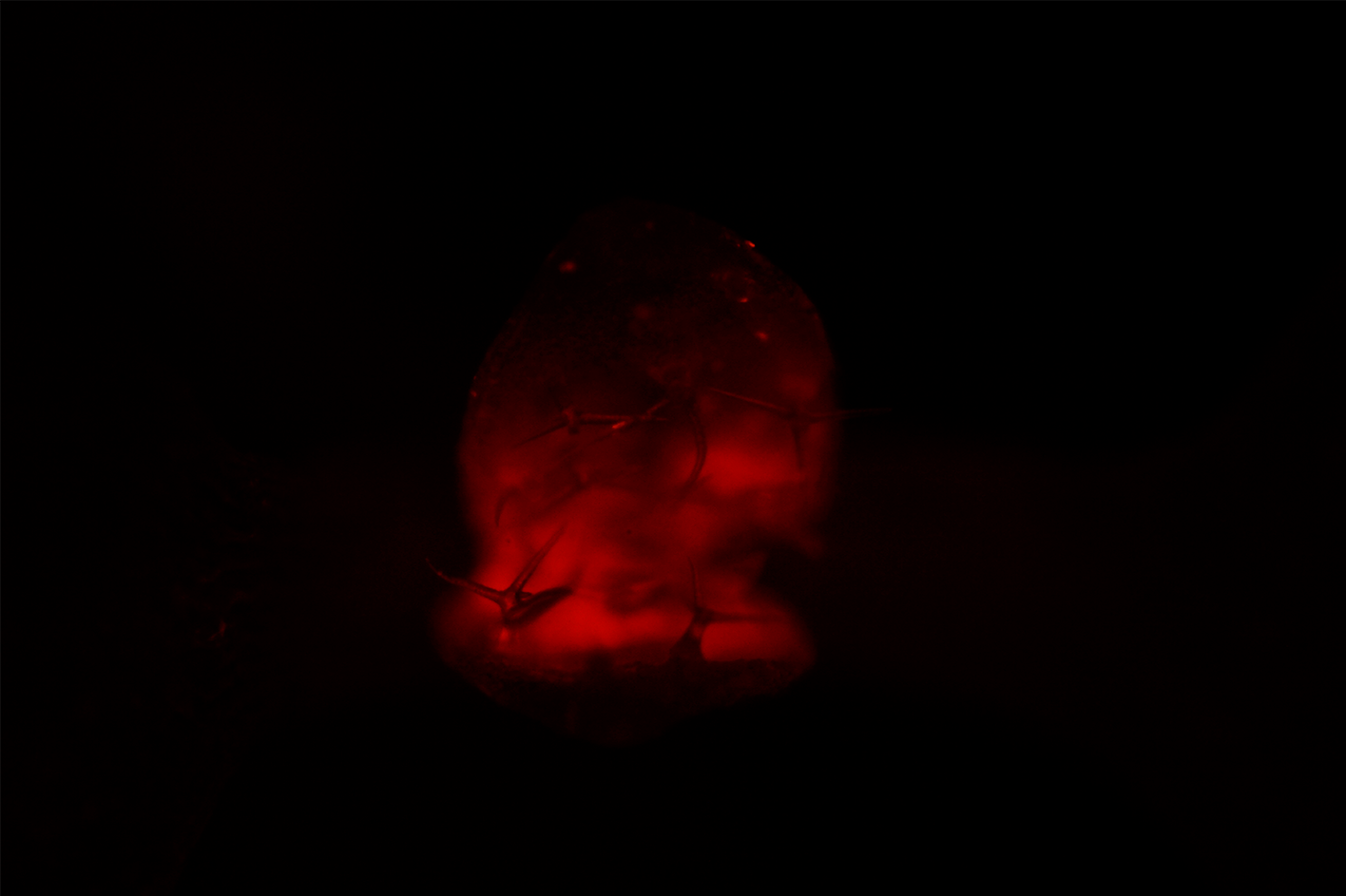

Supplement: Supplementary file 12 — Source data Fig. 3 [file 44318_2024_312_MOESM12_ESM.zip › Source data for Fig 3/3A/ECT2-mCh+ALBA2-TFP/ECT2-mCherry.tif]

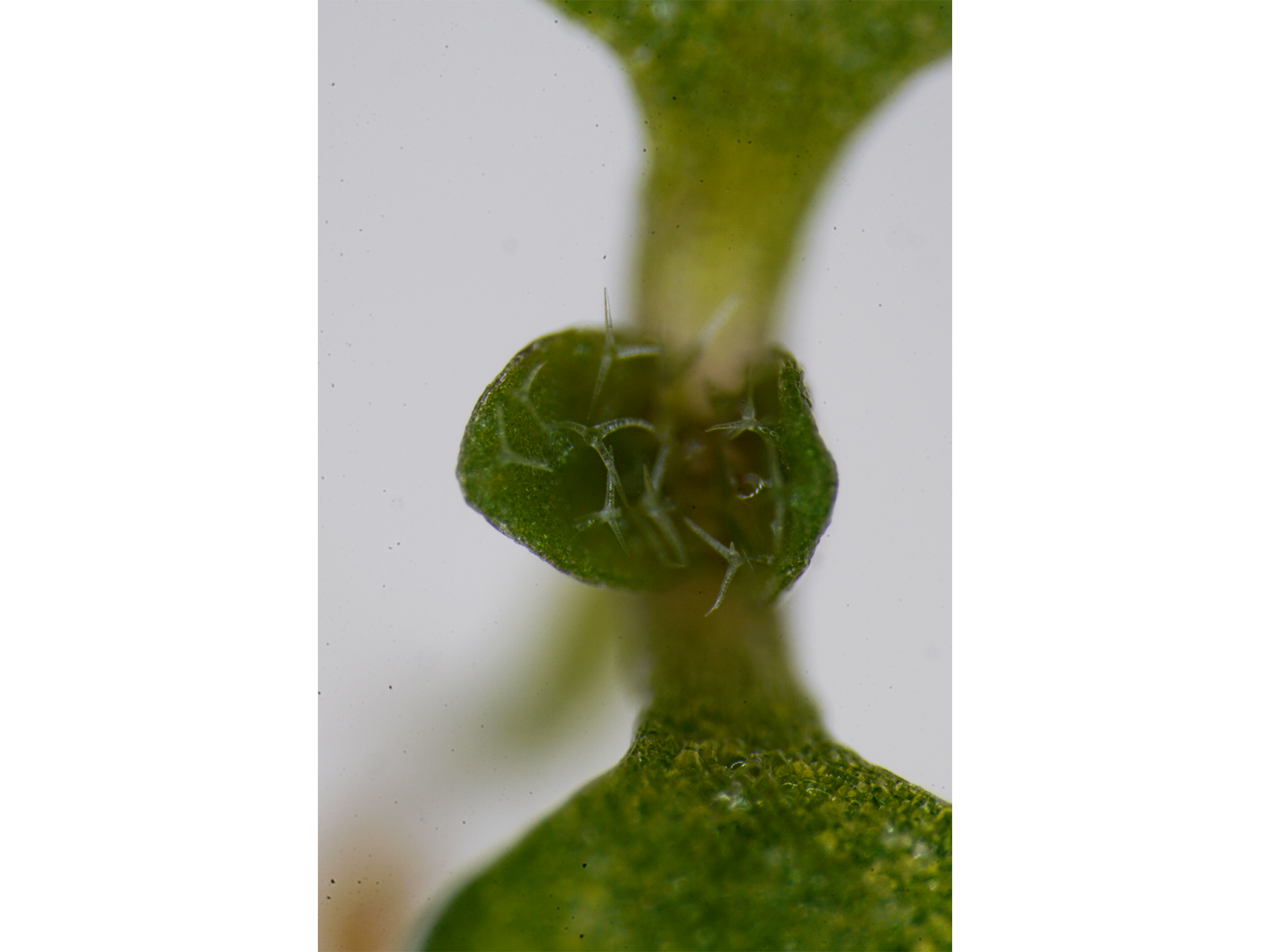

Supplement: Supplementary file 12 — Source data Fig. 3 [file 44318_2024_312_MOESM12_ESM.zip › Source data for Fig 3/3A/ECT2-mCh+ALBA2-TFP/Bright field.tif]

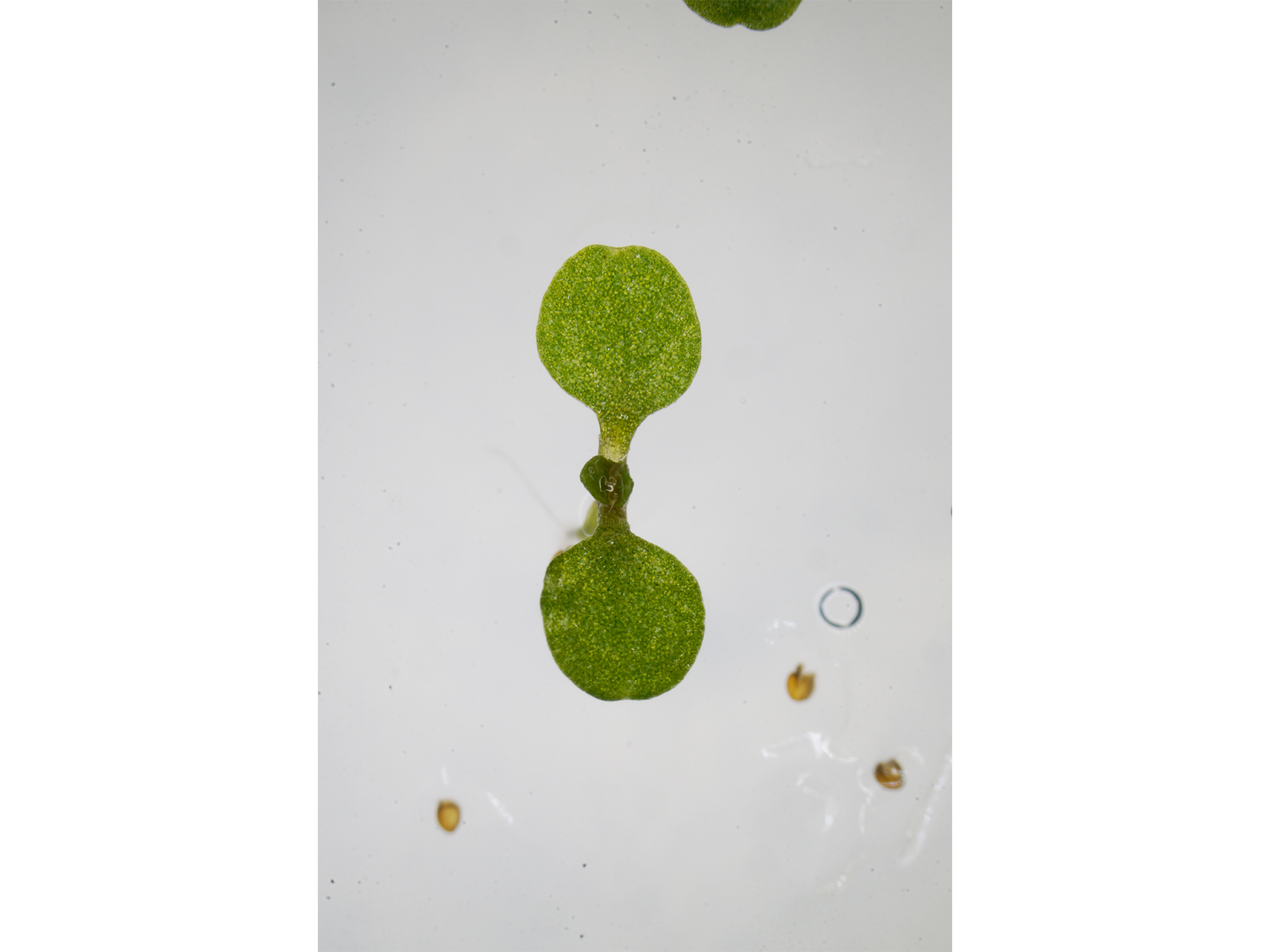

Supplement: Supplementary file 12 — Source data Fig. 3 [file 44318_2024_312_MOESM12_ESM.zip › Source data for Fig 3/3A/ECT2-mCh+ALBA2-TFP/Bright field seedling.tif]

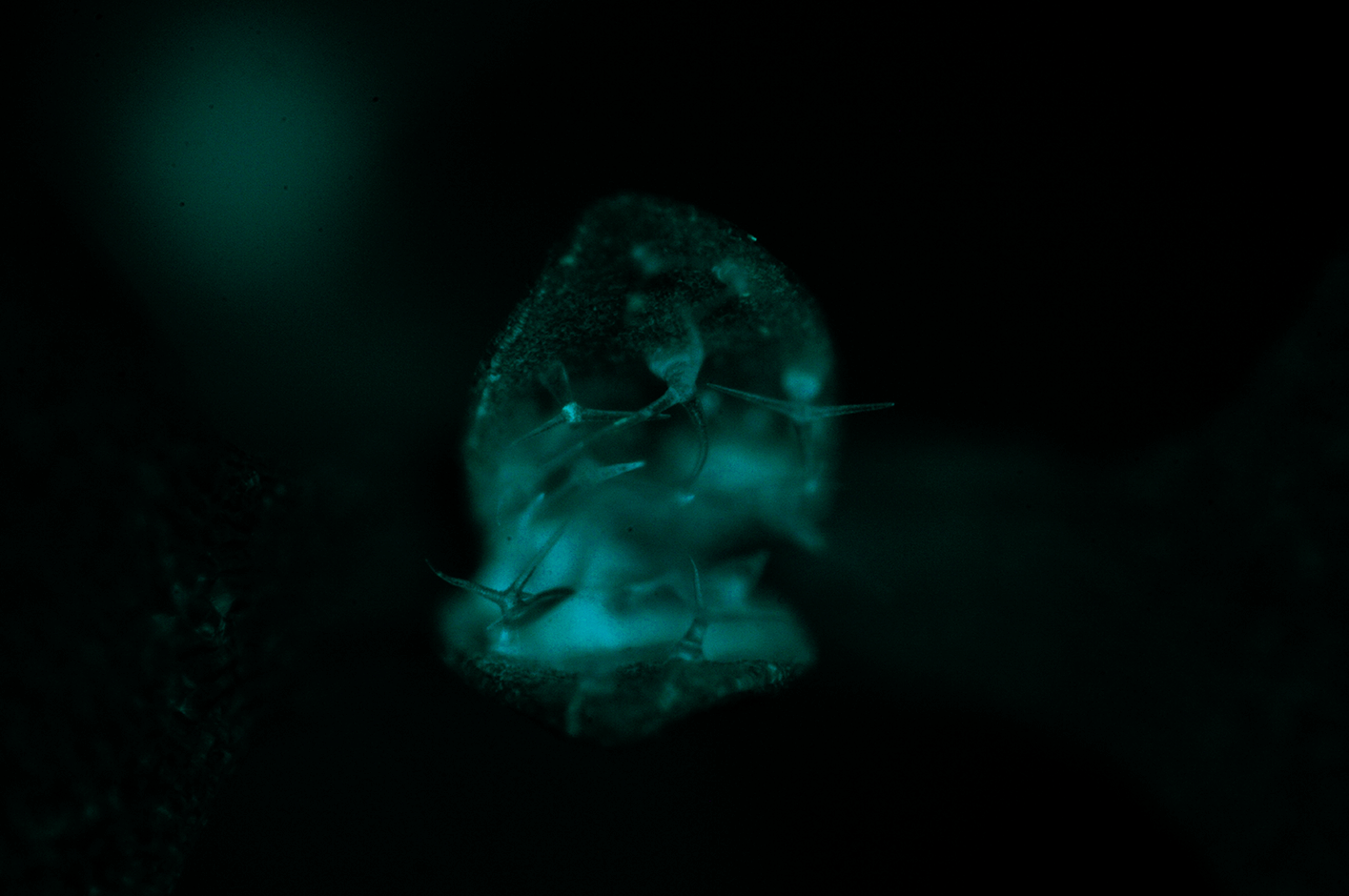

Supplement: Supplementary file 12 — Source data Fig. 3 [file 44318_2024_312_MOESM12_ESM.zip › Source data for Fig 3/3A/ECT2-mCh+ALBA2-TFP/ALBA2-TFP.tif]

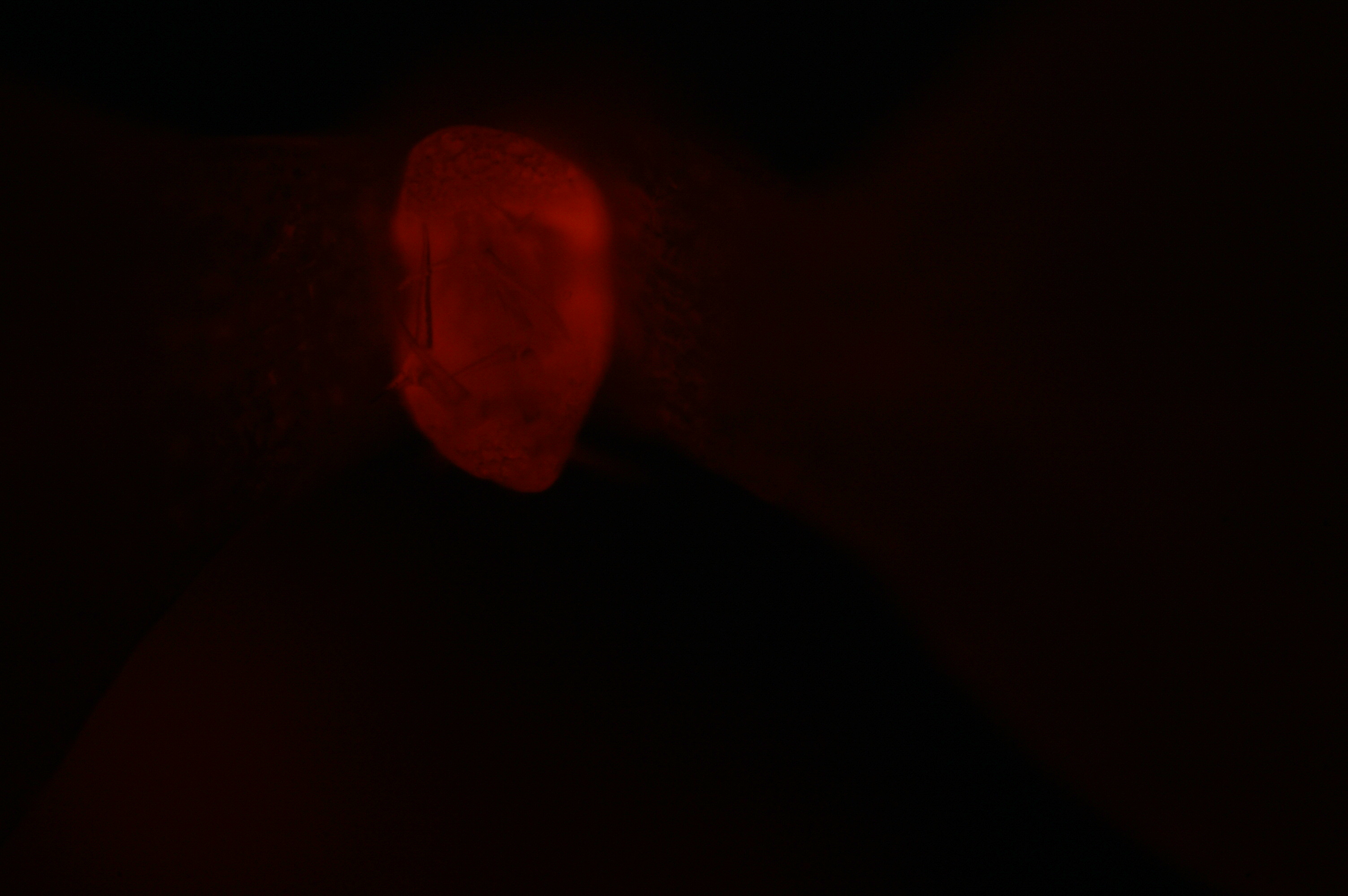

Supplement: Supplementary file 12 — Source data Fig. 3 [file 44318_2024_312_MOESM12_ESM.zip › Source data for Fig 3/3A/ECT2-mCh+ALBA1-TFP/ECT2-mCherry.tif]

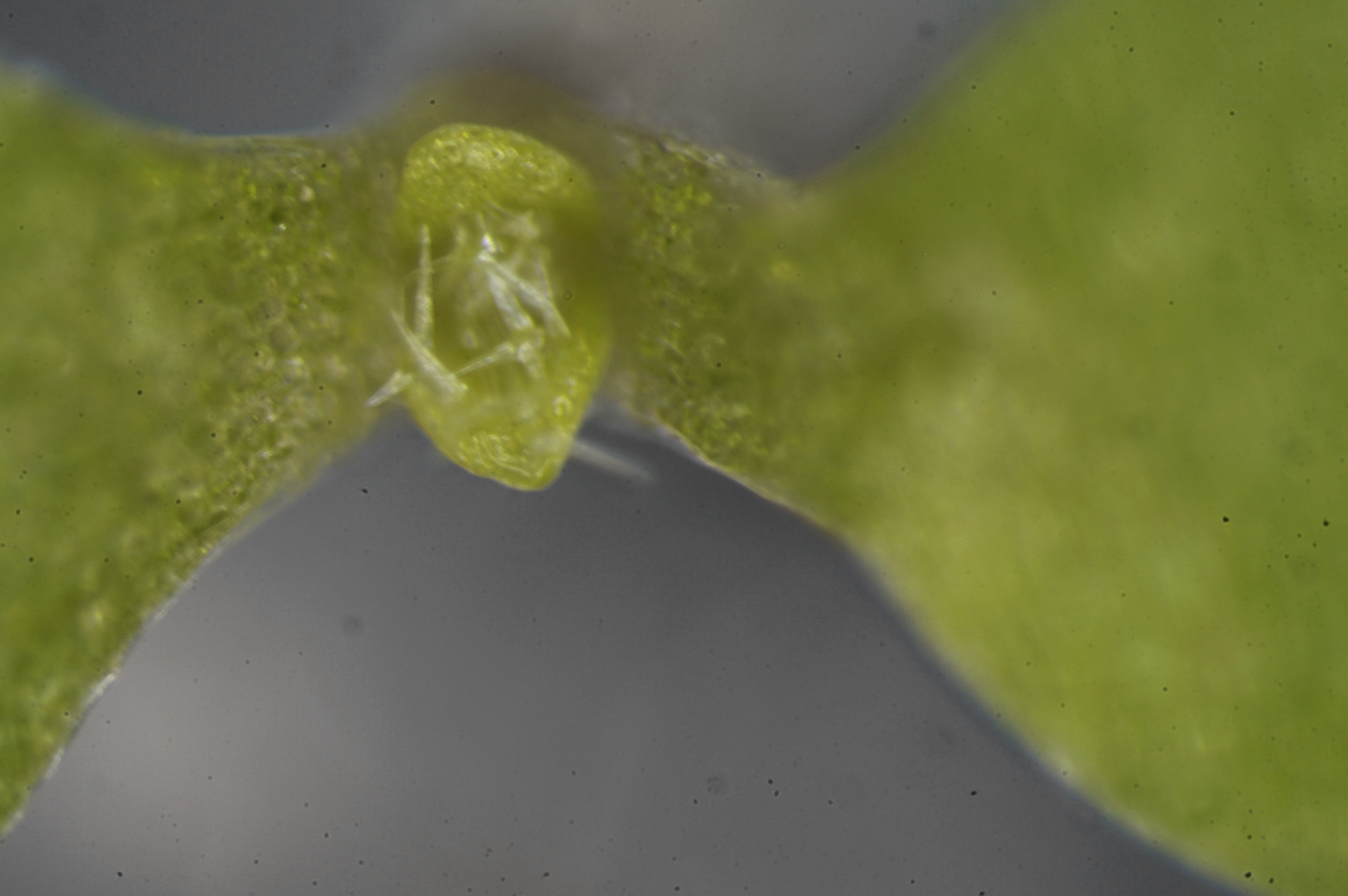

Supplement: Supplementary file 12 — Source data Fig. 3 [file 44318_2024_312_MOESM12_ESM.zip › Source data for Fig 3/3A/ECT2-mCh+ALBA1-TFP/Bright field.tif]

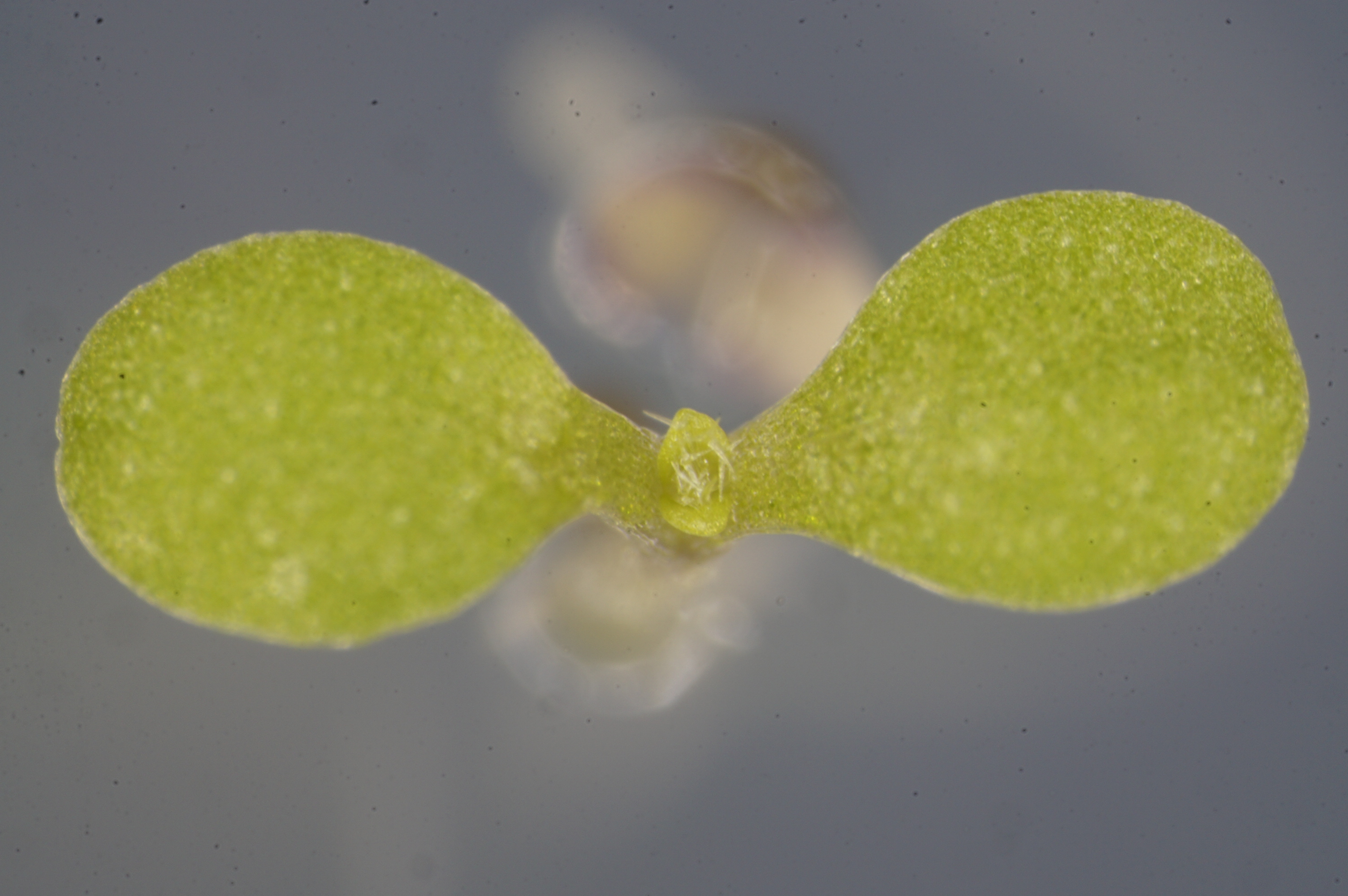

Supplement: Supplementary file 12 — Source data Fig. 3 [file 44318_2024_312_MOESM12_ESM.zip › Source data for Fig 3/3A/ECT2-mCh+ALBA1-TFP/Bright field seedling.JPG]

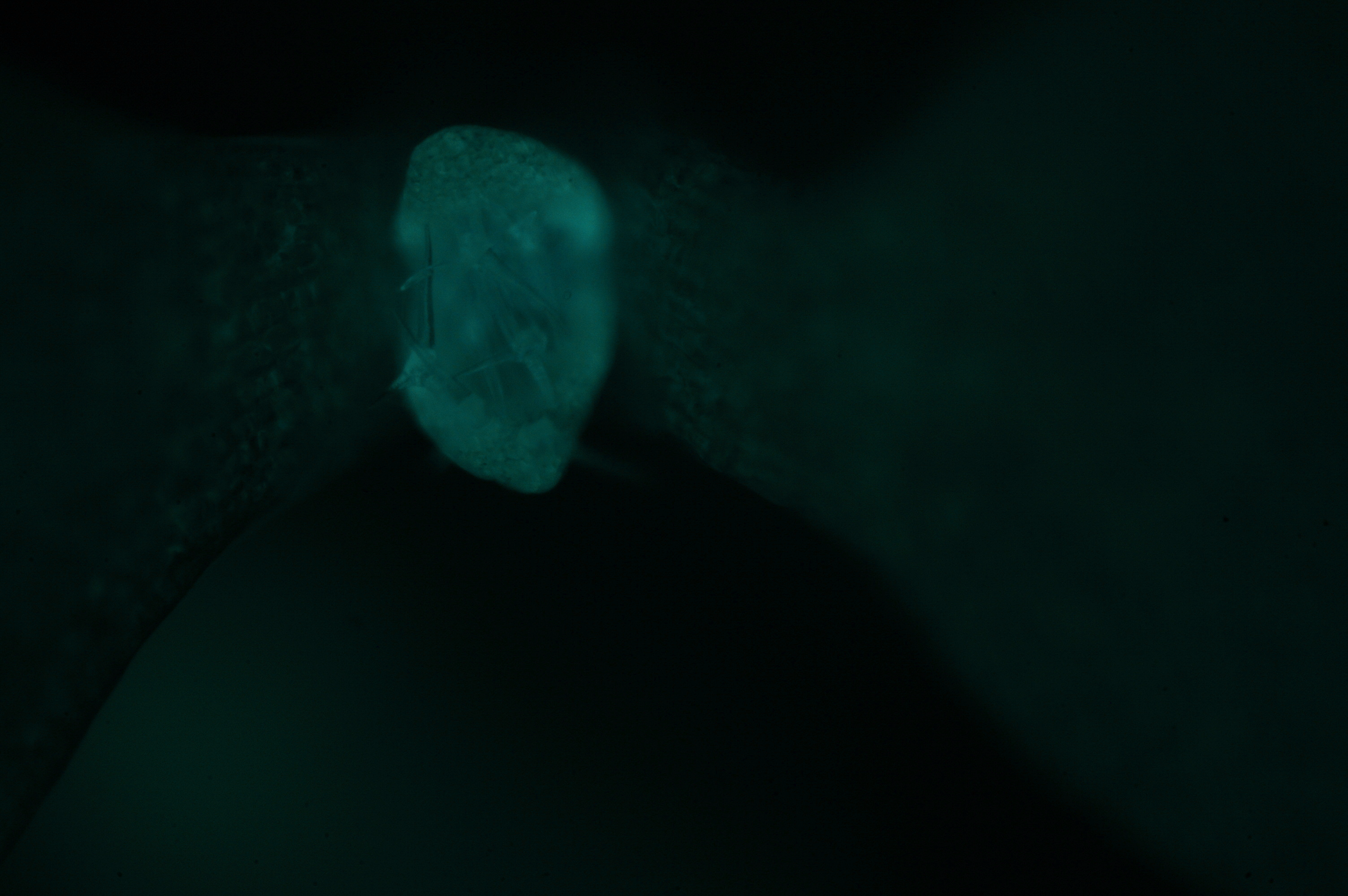

Supplement: Supplementary file 12 — Source data Fig. 3 [file 44318_2024_312_MOESM12_ESM.zip › Source data for Fig 3/3A/ECT2-mCh+ALBA1-TFP/ALBA1-TFP.tif]

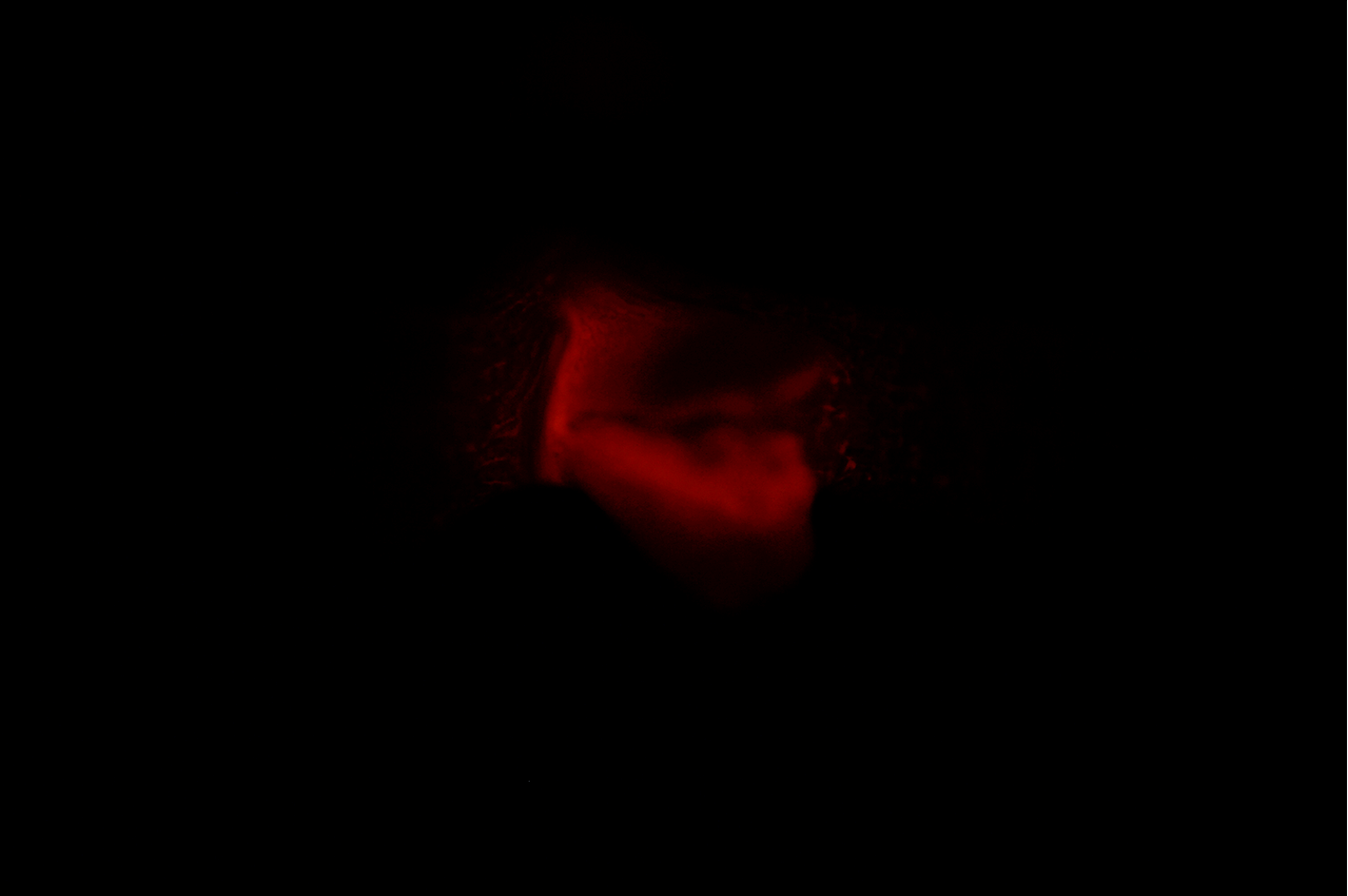

Supplement: Supplementary file 12 — Source data Fig. 3 [file 44318_2024_312_MOESM12_ESM.zip › Source data for Fig 3/3A/ECT2-mCh+ALBA4-Venus/ECT2-mCh.tif]

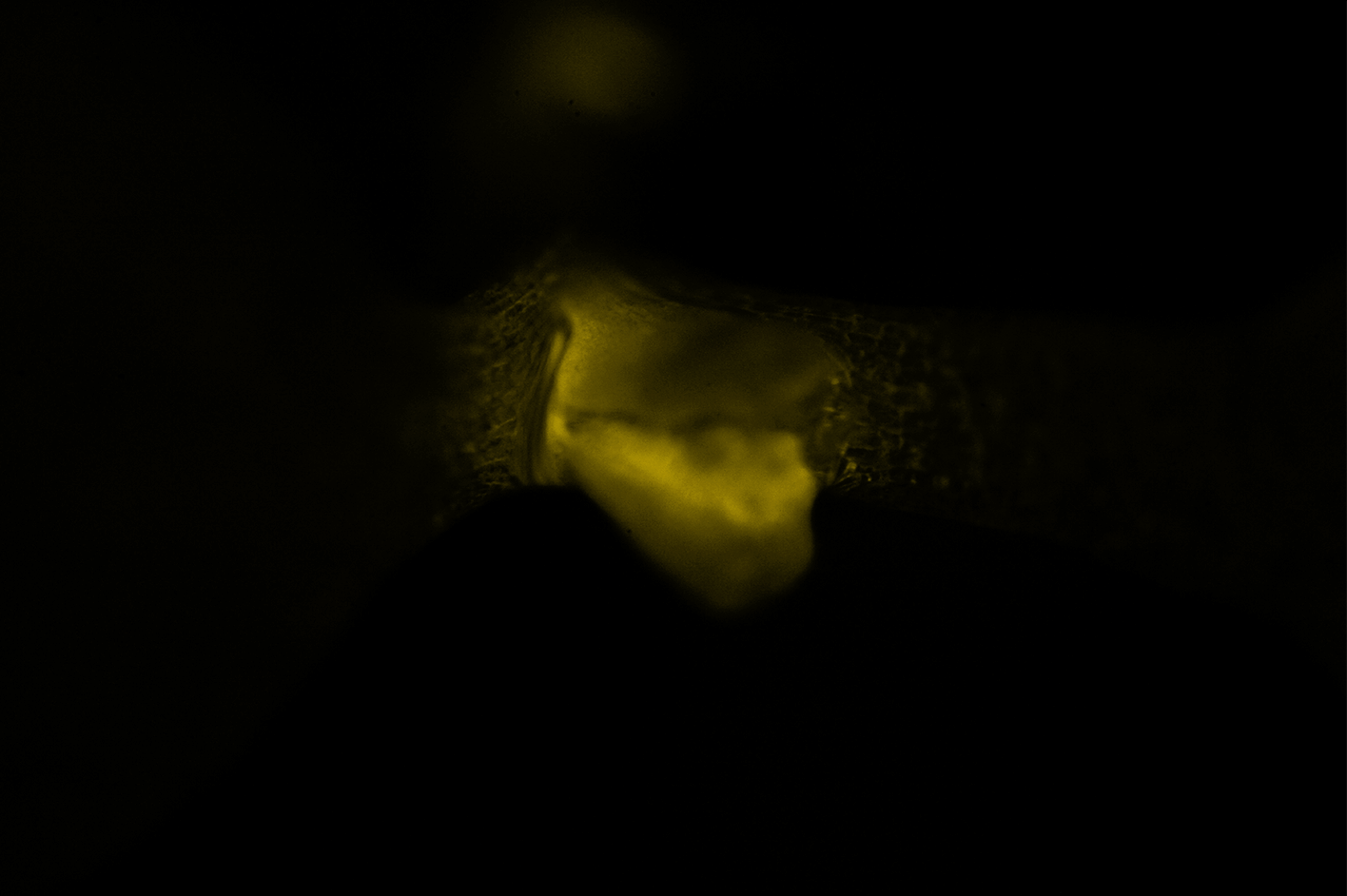

Supplement: Supplementary file 12 — Source data Fig. 3 [file 44318_2024_312_MOESM12_ESM.zip › Source data for Fig 3/3A/ECT2-mCh+ALBA4-Venus/ALBA4-Venus.tif]

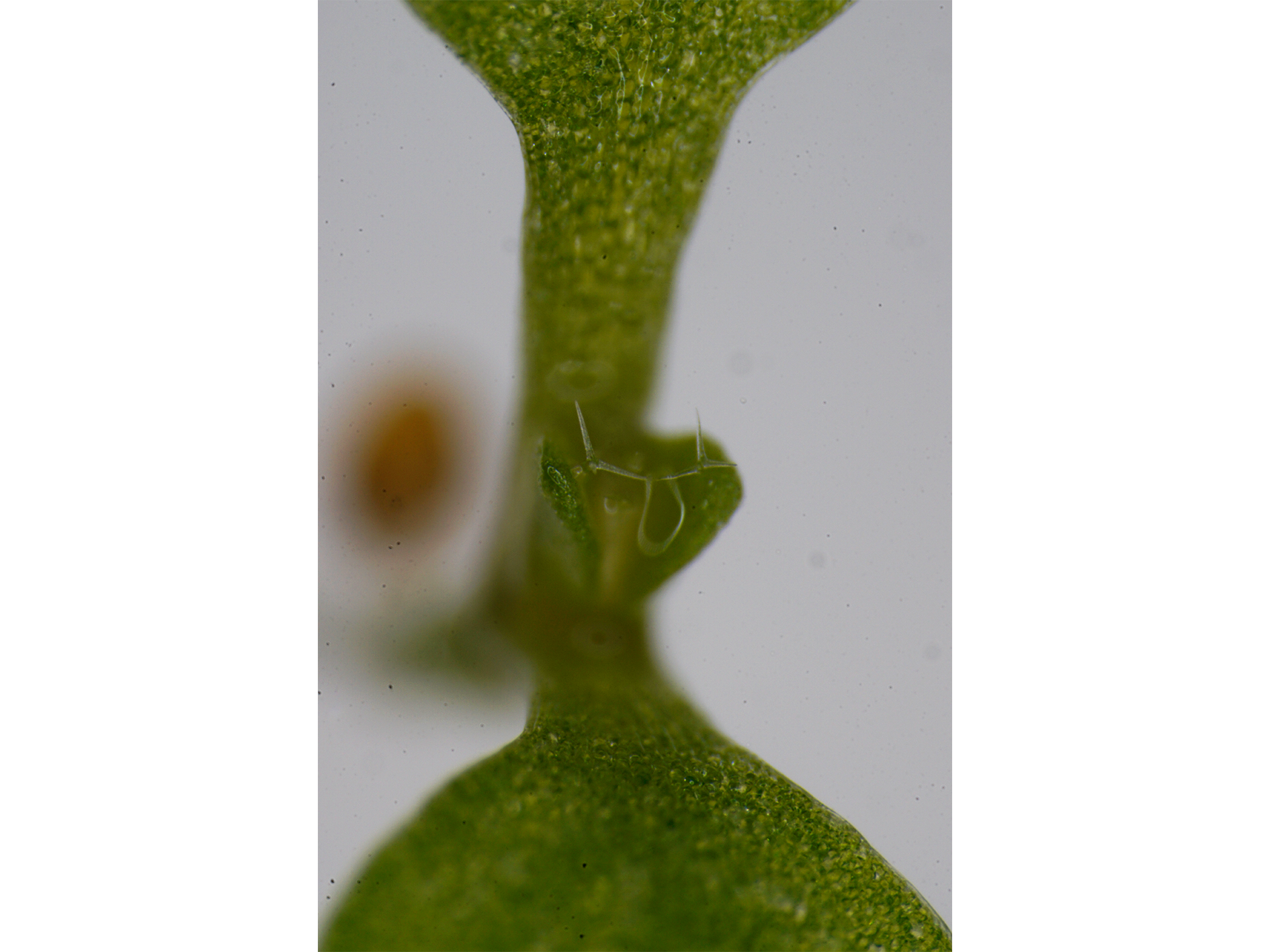

Supplement: Supplementary file 12 — Source data Fig. 3 [file 44318_2024_312_MOESM12_ESM.zip › Source data for Fig 3/3A/ECT2-mCh+ALBA4-Venus/Bright field.tif]

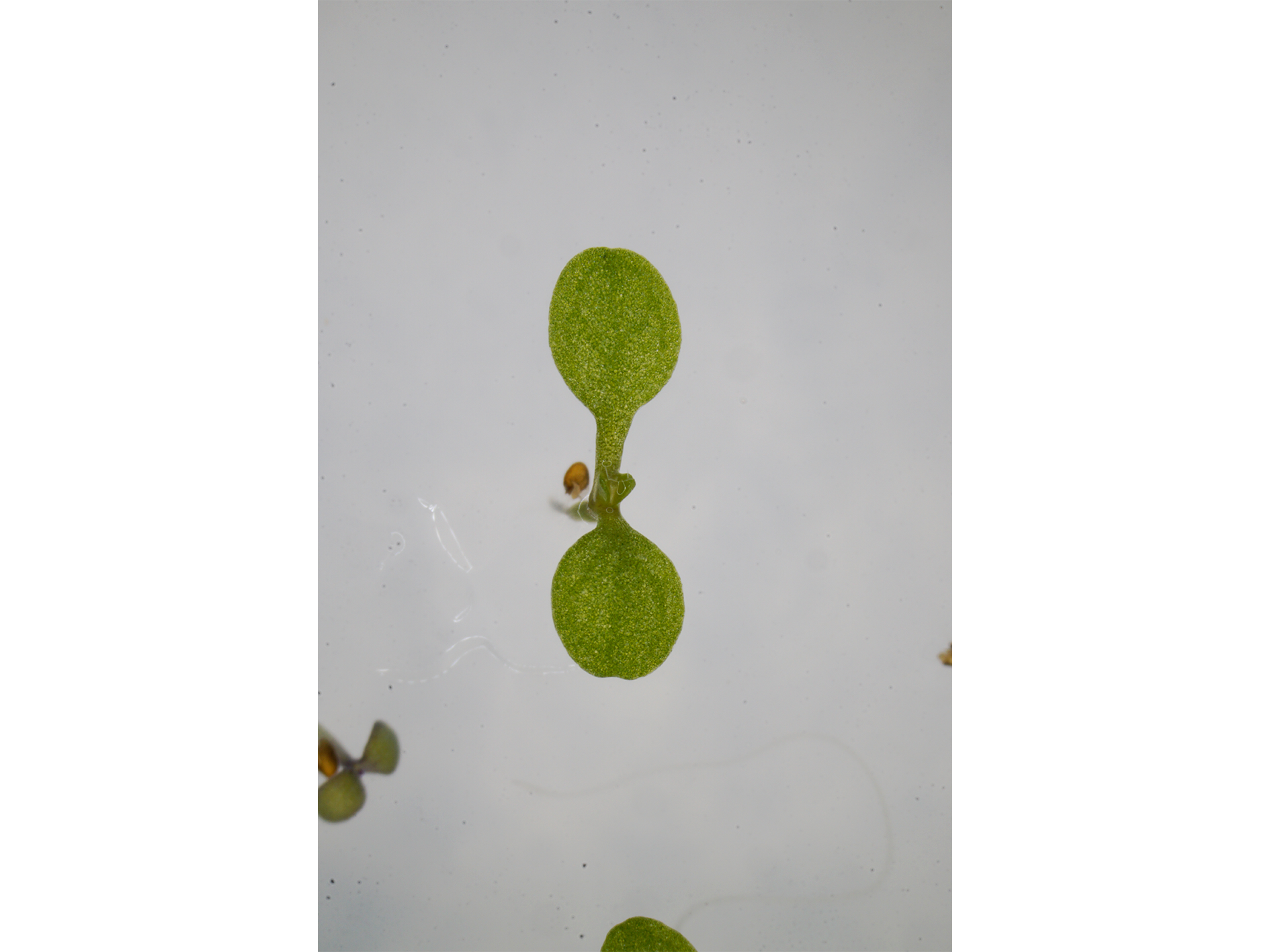

Supplement: Supplementary file 12 — Source data Fig. 3 [file 44318_2024_312_MOESM12_ESM.zip › Source data for Fig 3/3A/ECT2-mCh+ALBA4-Venus/Bright field seedling.tif]

Source data for Figure 4A

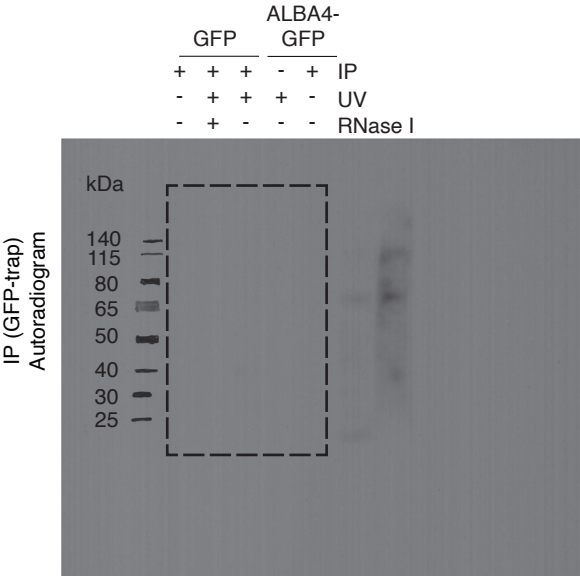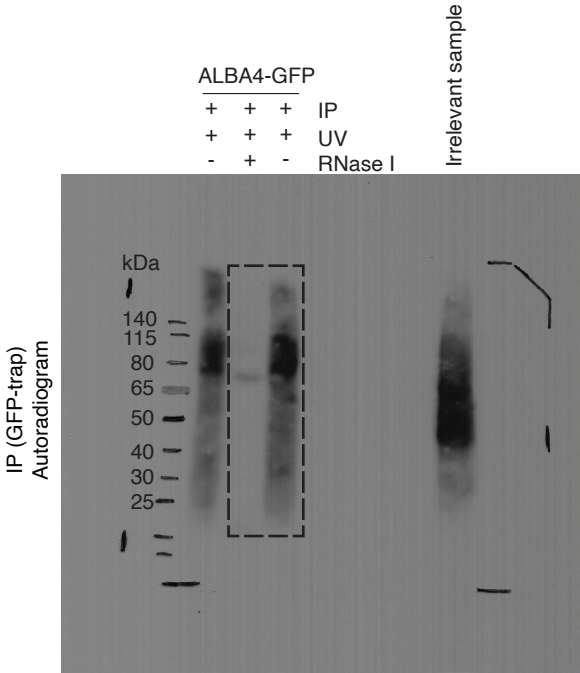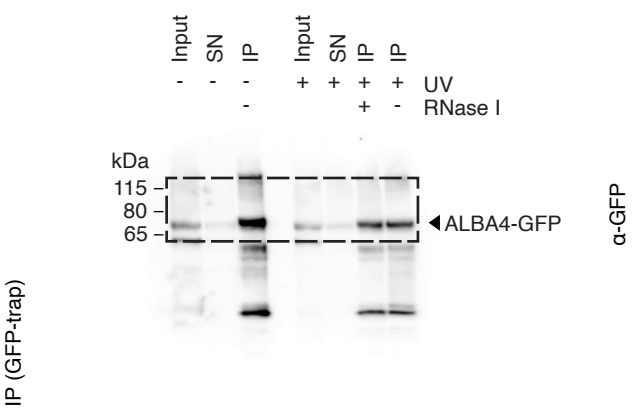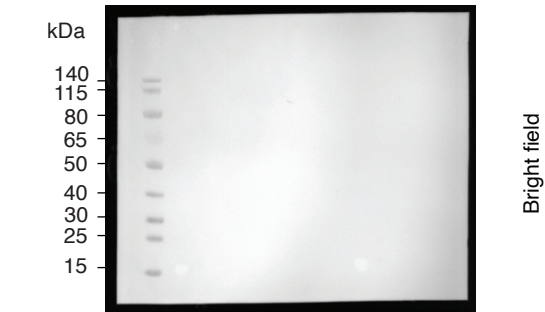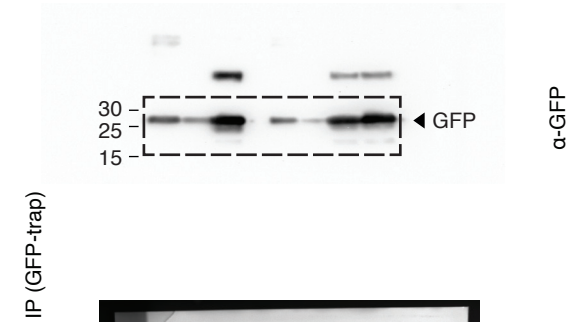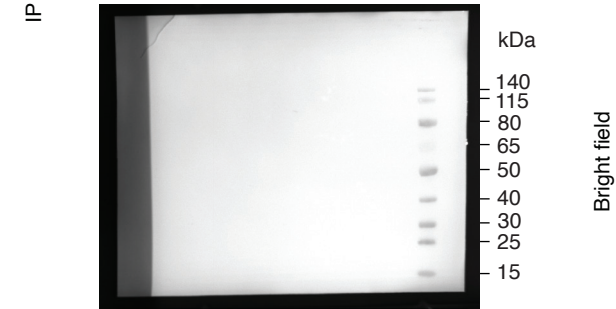

Supplement: Supplementary file 13 — Source data Fig. 4 [file 44318_2024_312_MOESM13_ESM.zip › Source data for Fig 4/4A/Autoradiogram and western after UV-Xlink and CoIP.pdf]

Source data for Figure 7A

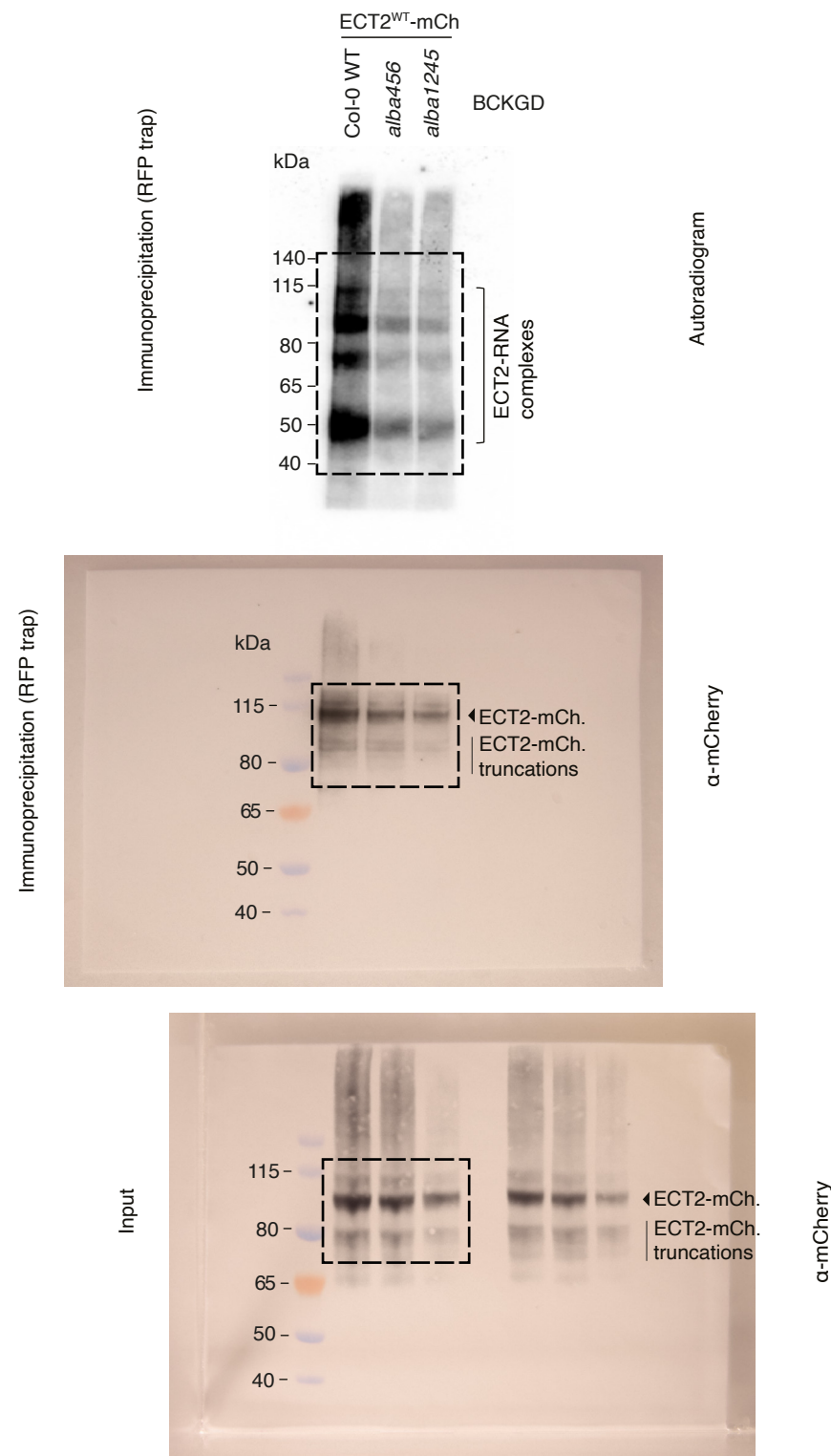

Supplement: Supplementary file 14 — Source data Fig. 7 [file 44318_2024_312_MOESM14_ESM.zip › Source data for Fig 7/7A/Autoradiogram and western after UV-Xlink and CoIP.pdf]

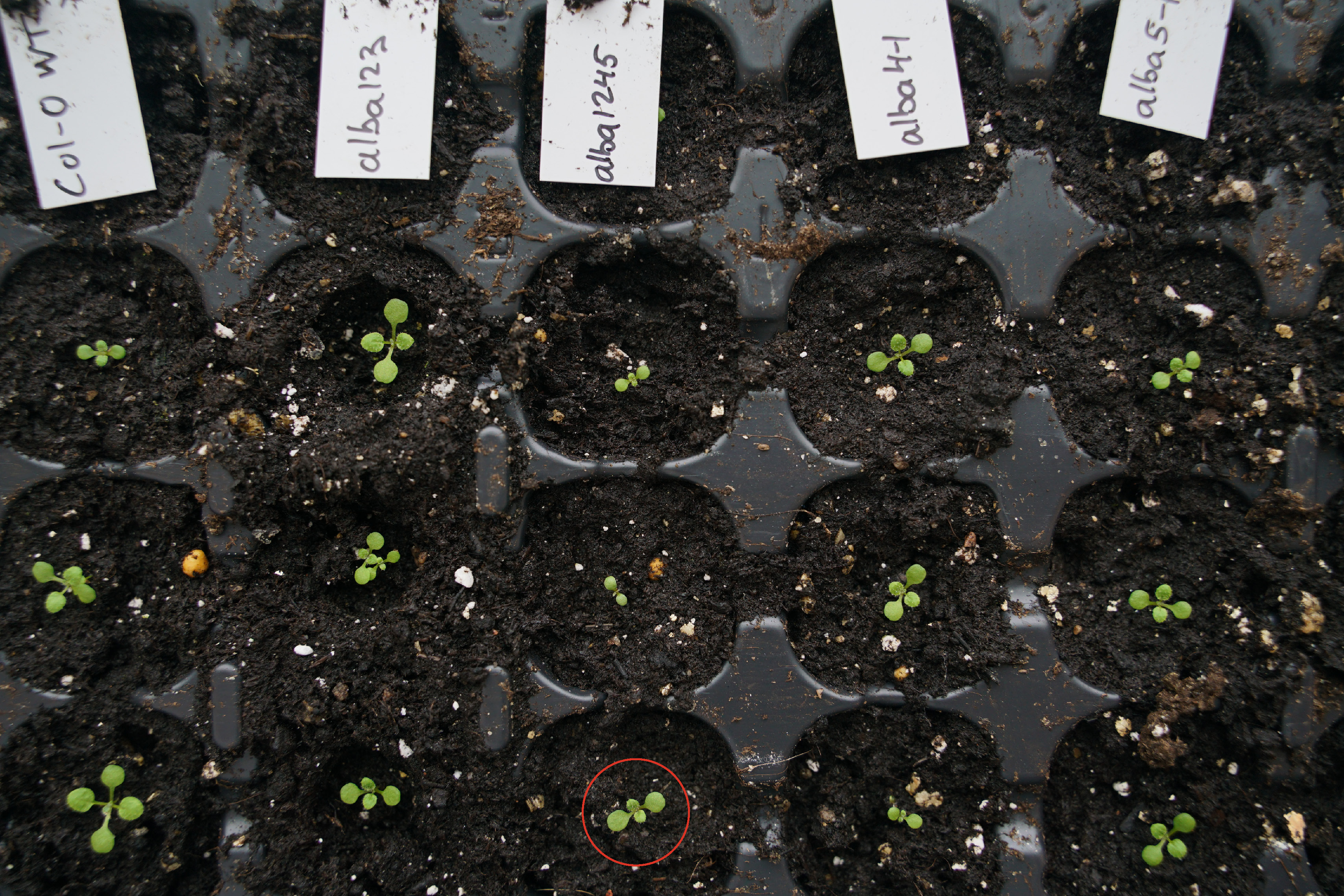

Supplement: Supplementary file 14 — Source data Fig. 7 [file 44318_2024_312_MOESM14_ESM.zip › Source data for Fig 7/7F/10 DAG/alba1245.jpg]

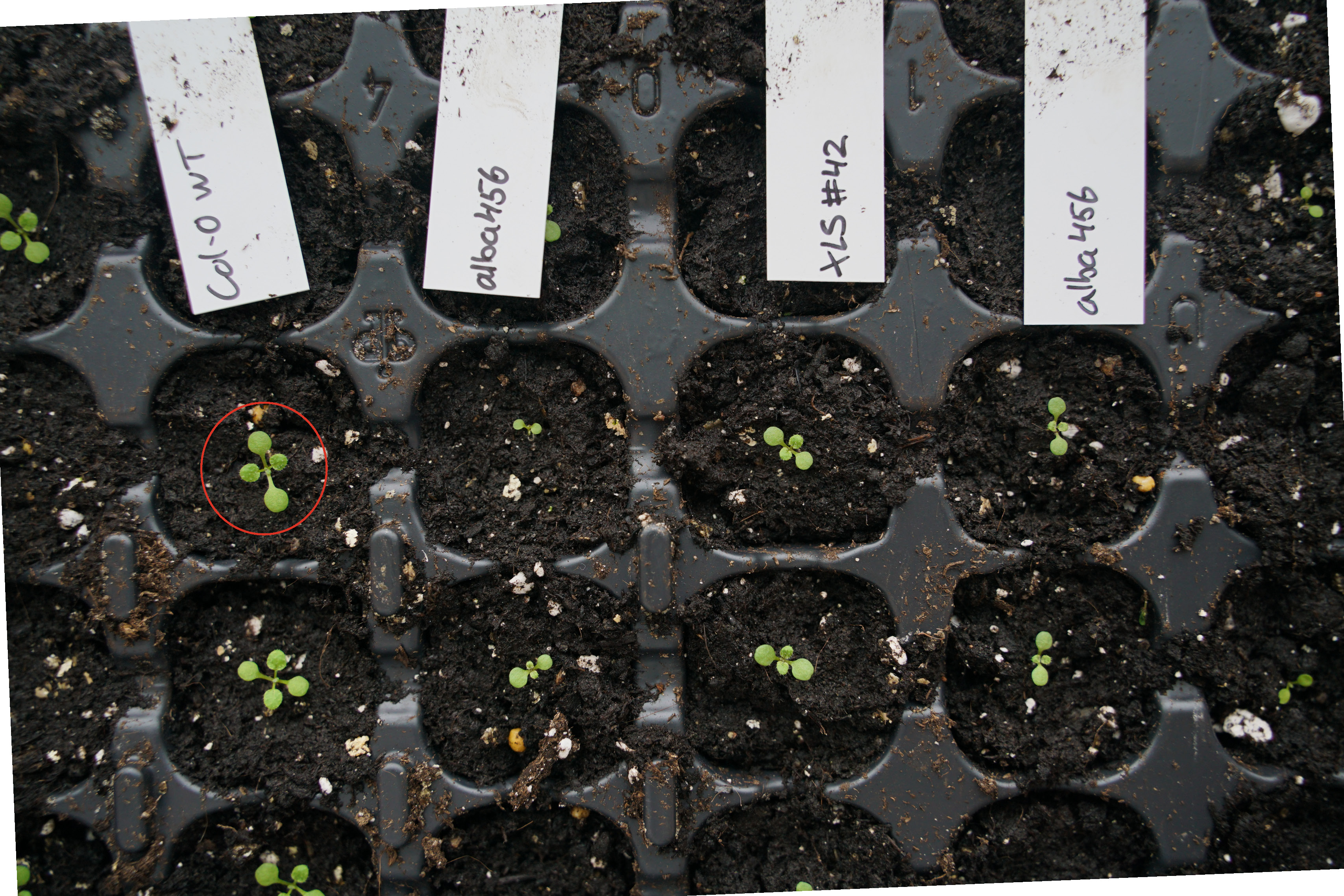

Supplement: Supplementary file 14 — Source data Fig. 7 [file 44318_2024_312_MOESM14_ESM.zip › Source data for Fig 7/7F/10 DAG/Col-0 WT.jpg]

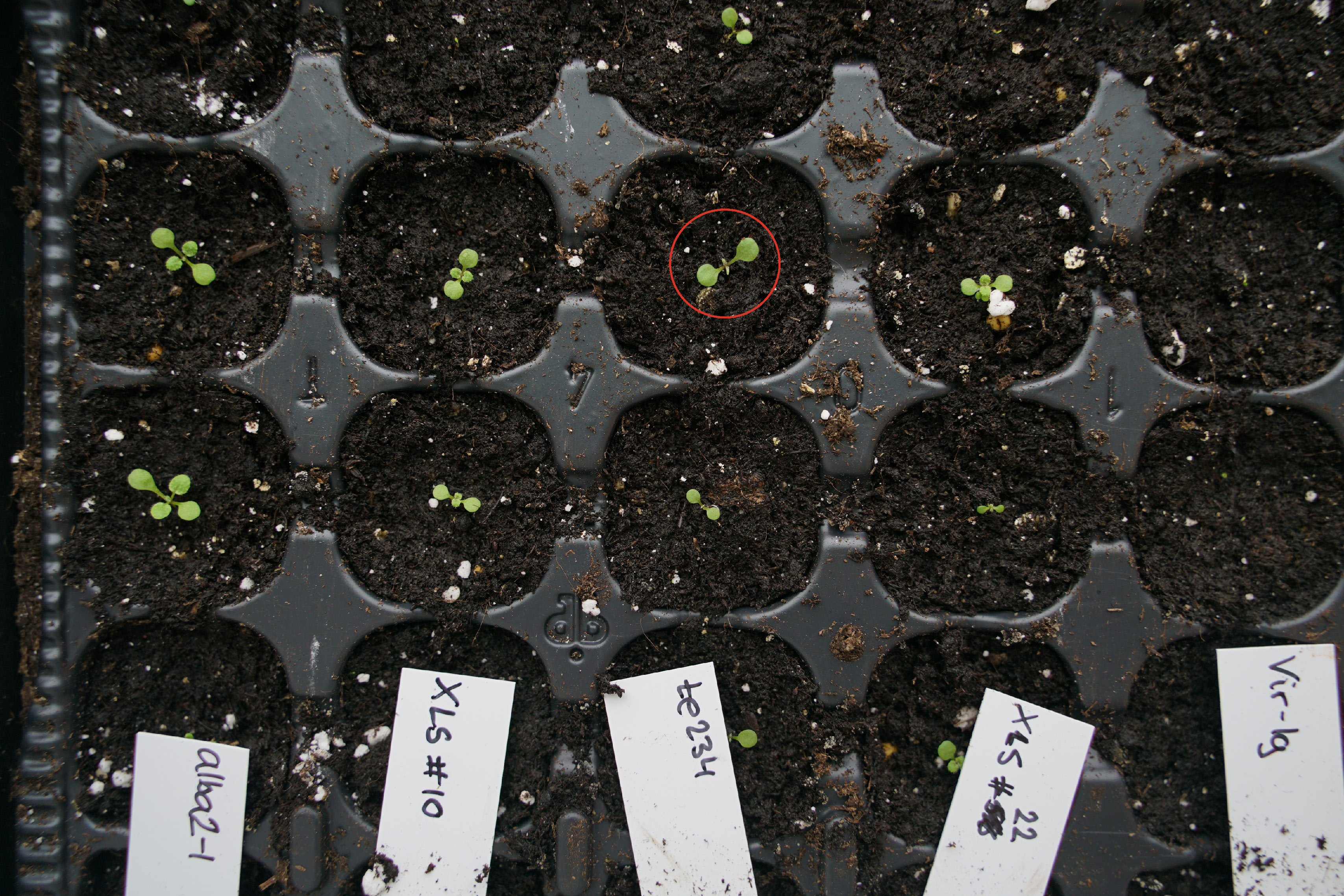

Supplement: Supplementary file 14 — Source data Fig. 7 [file 44318_2024_312_MOESM14_ESM.zip › Source data for Fig 7/7F/10 DAG/te234.jpg]

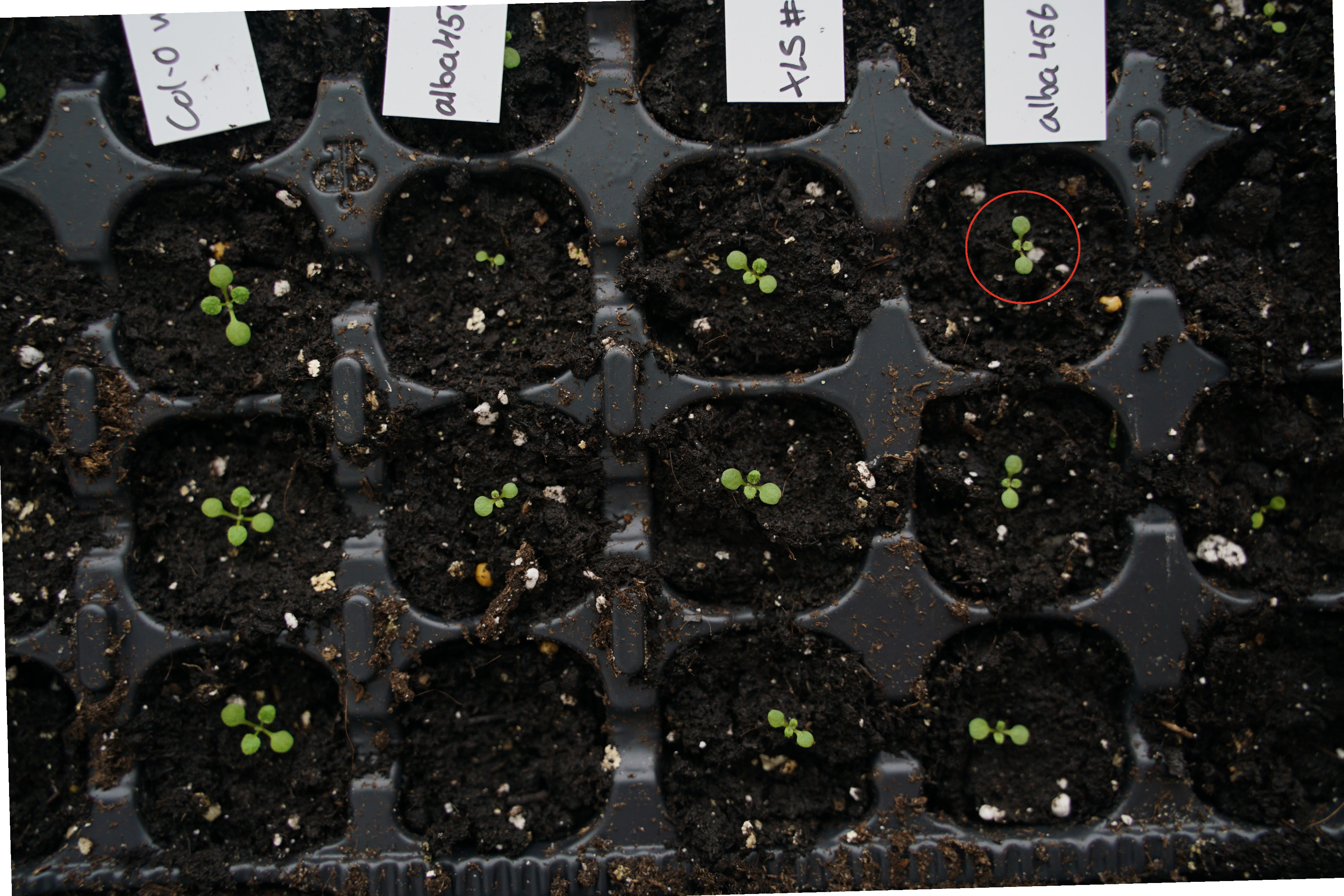

Supplement: Supplementary file 14 — Source data Fig. 7 [file 44318_2024_312_MOESM14_ESM.zip › Source data for Fig 7/7F/10 DAG/alba456.jpg]

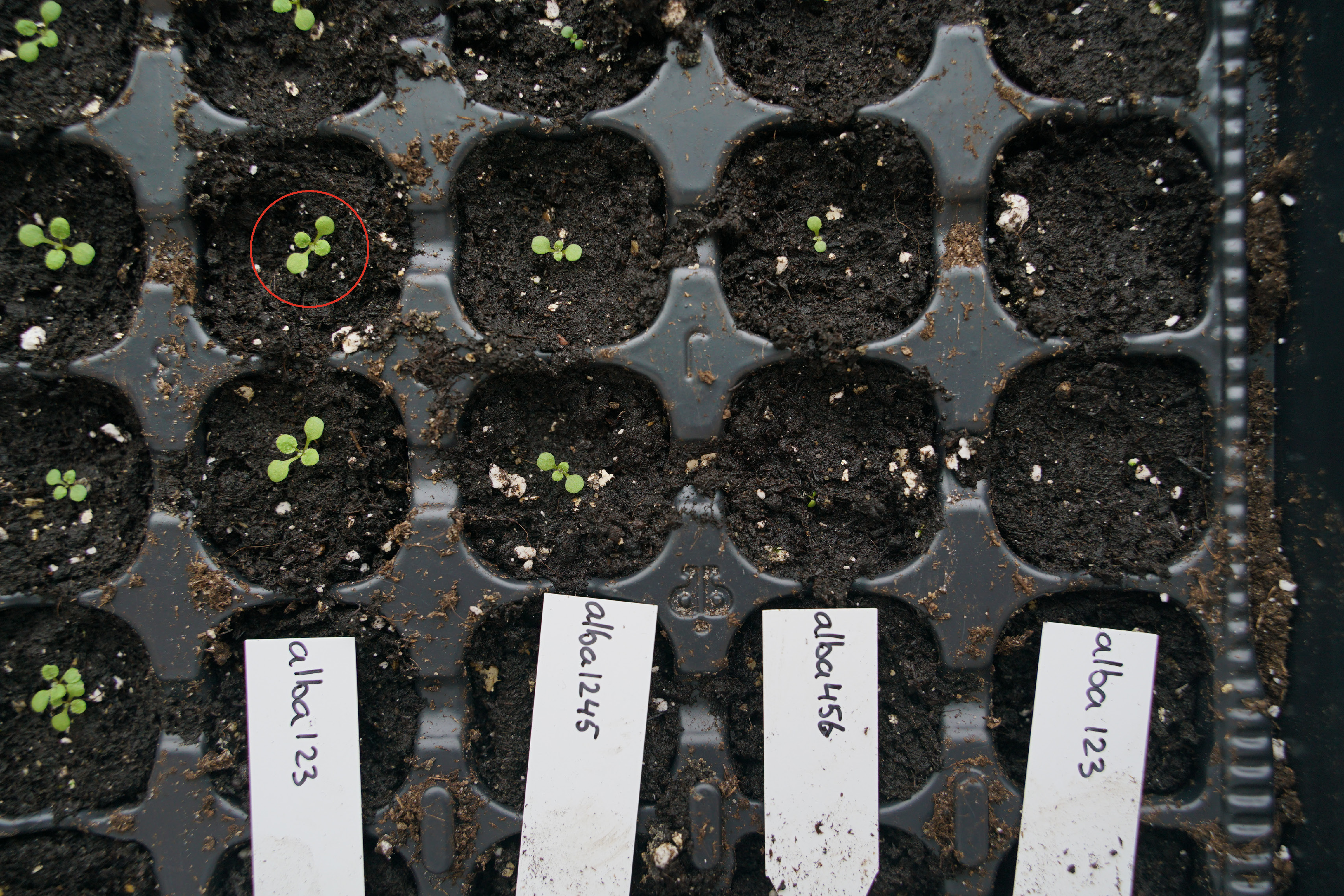

Supplement: Supplementary file 14 — Source data Fig. 7 [file 44318_2024_312_MOESM14_ESM.zip › Source data for Fig 7/7F/10 DAG/alba123.jpg]

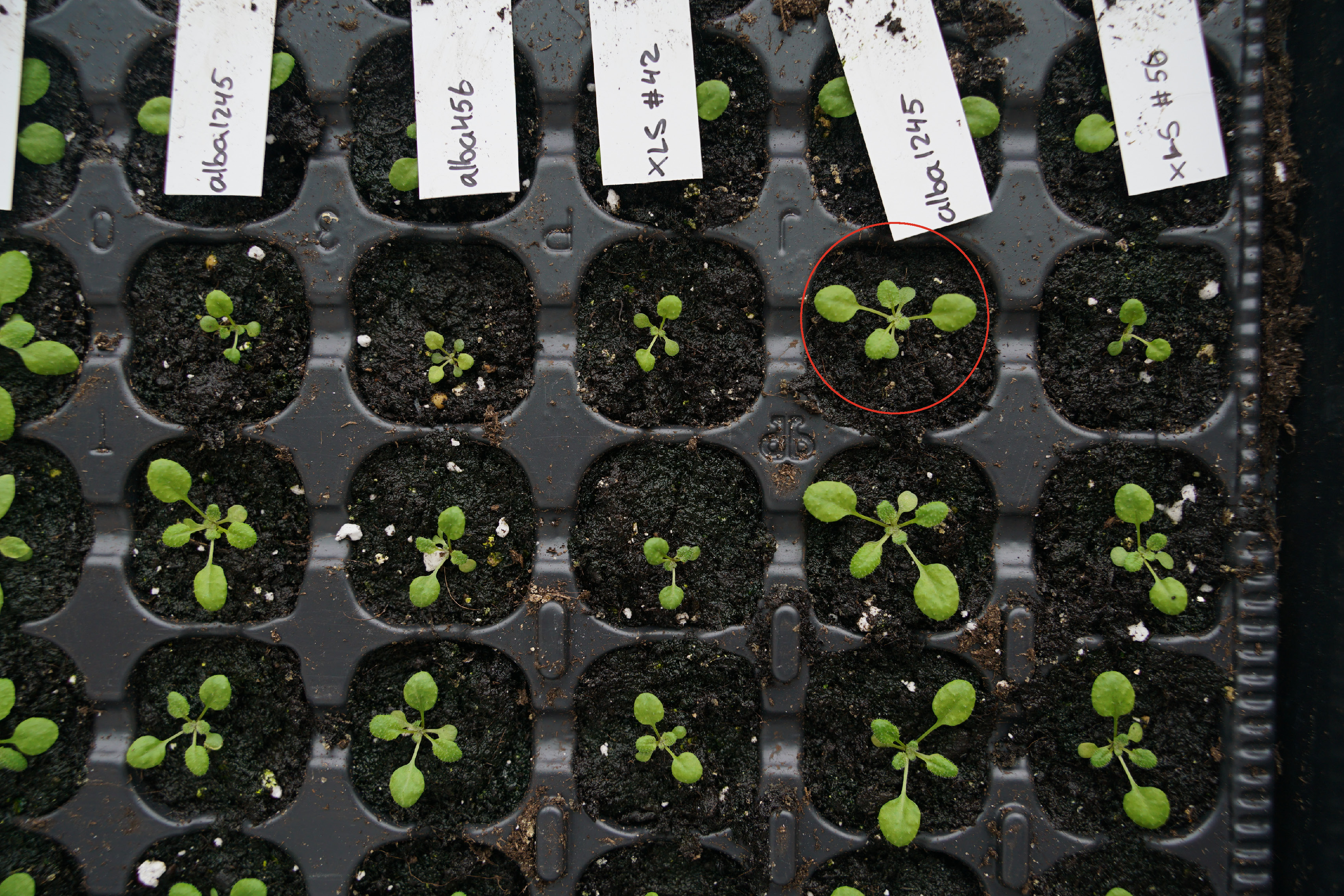

Supplement: Supplementary file 14 — Source data Fig. 7 [file 44318_2024_312_MOESM14_ESM.zip › Source data for Fig 7/7F/17 DAG/alba1245.jpg]

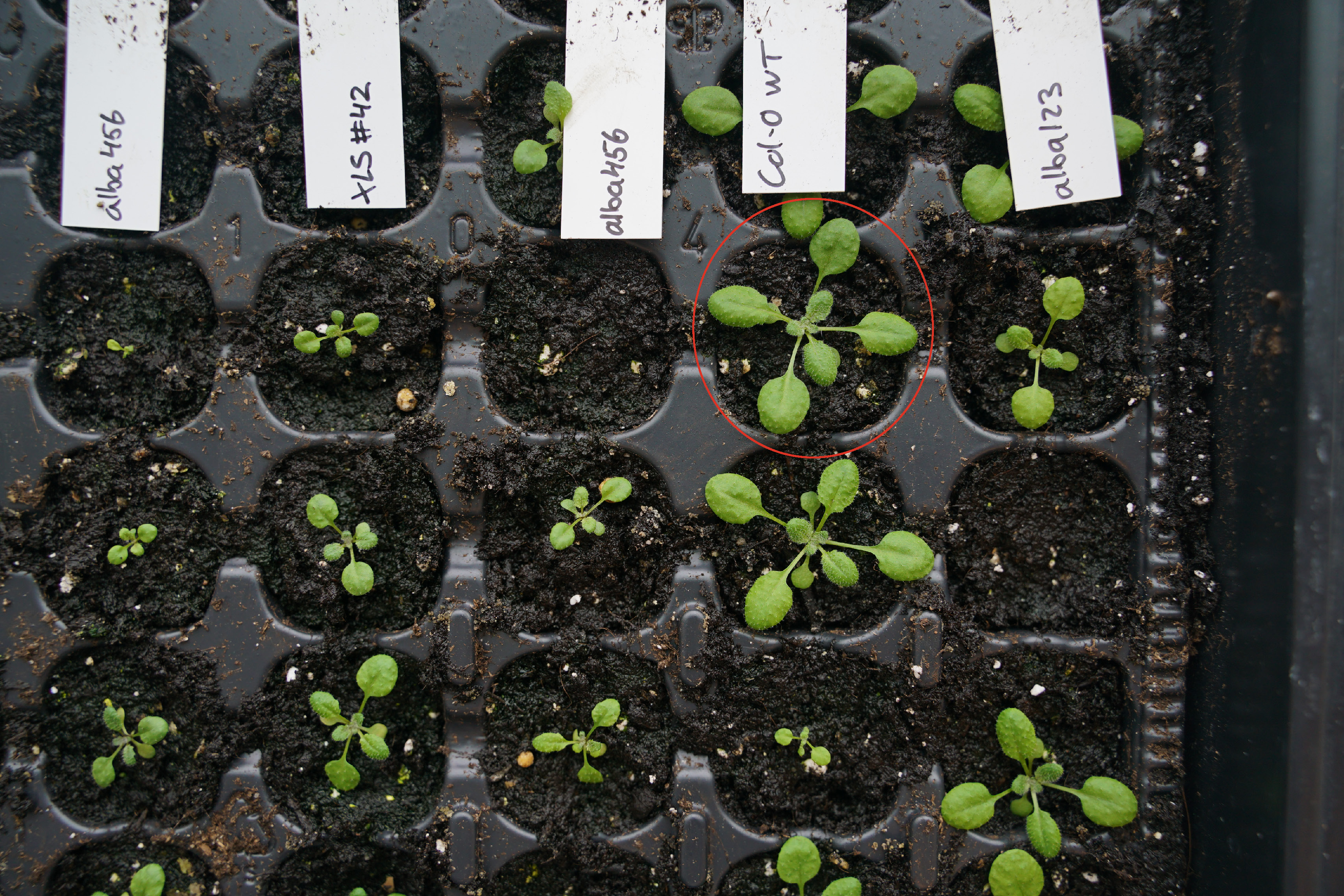

Supplement: Supplementary file 14 — Source data Fig. 7 [file 44318_2024_312_MOESM14_ESM.zip › Source data for Fig 7/7F/17 DAG/Col-0 WT.jpg]

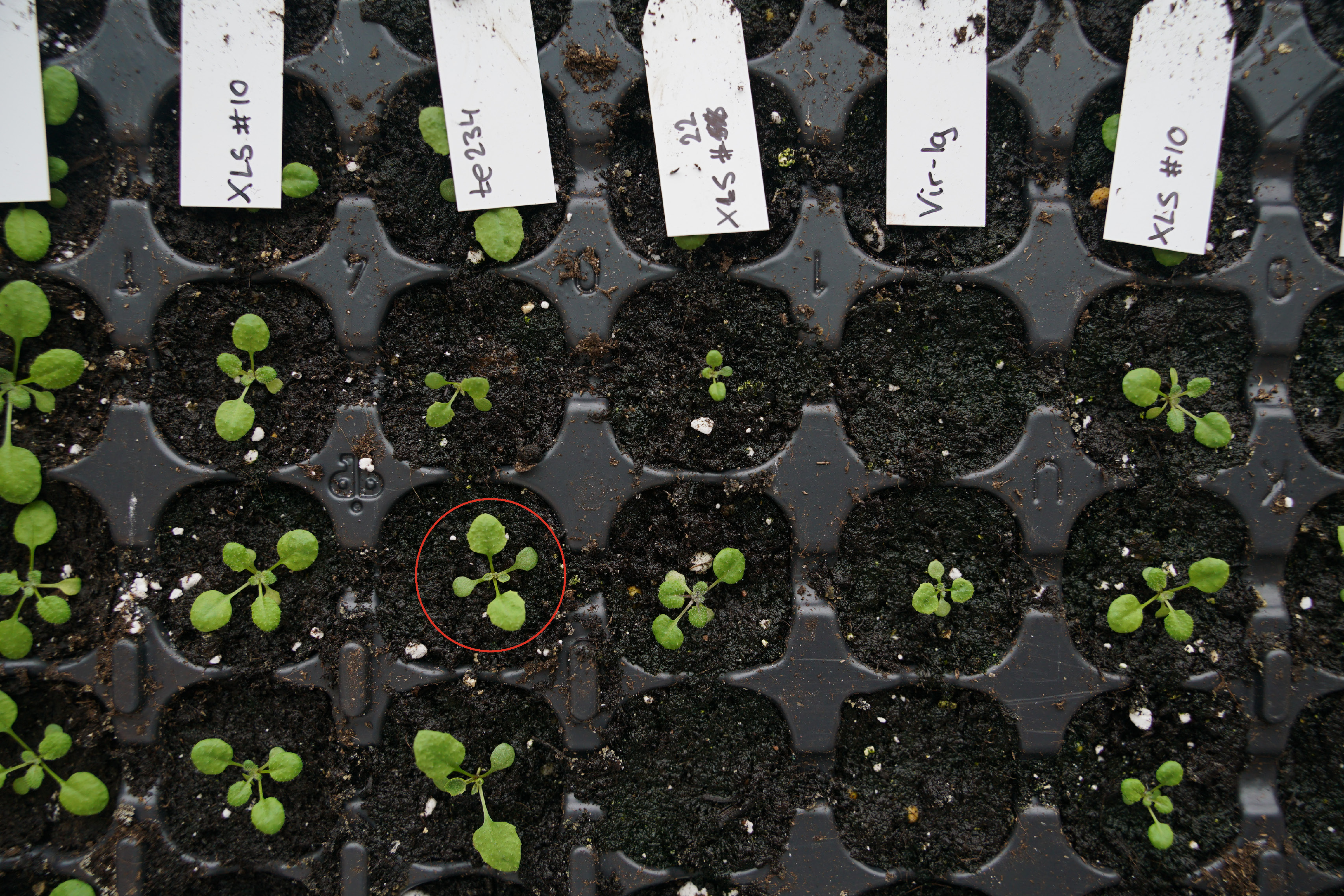

Supplement: Supplementary file 14 — Source data Fig. 7 [file 44318_2024_312_MOESM14_ESM.zip › Source data for Fig 7/7F/17 DAG/te234.jpg]

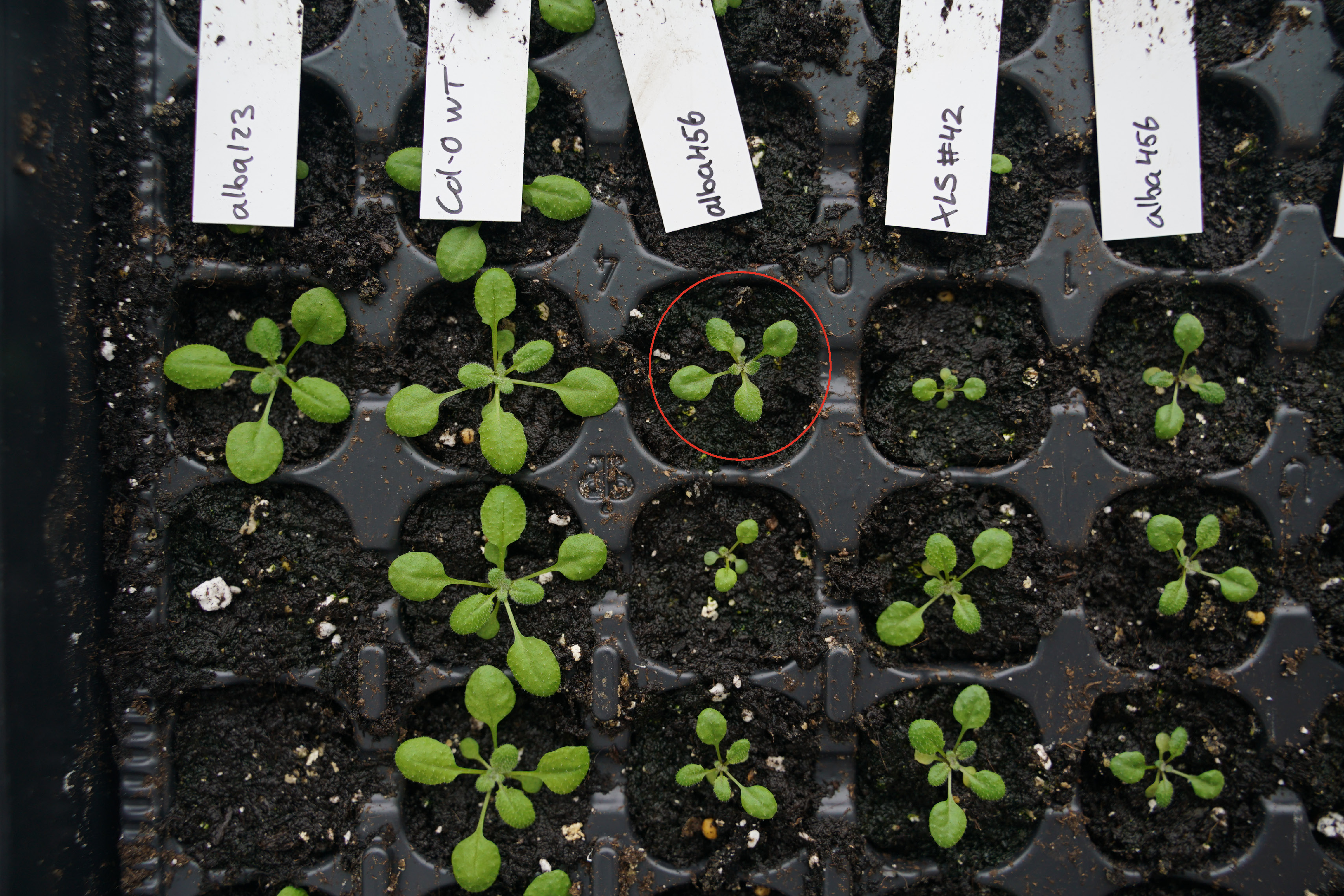

Supplement: Supplementary file 14 — Source data Fig. 7 [file 44318_2024_312_MOESM14_ESM.zip › Source data for Fig 7/7F/17 DAG/alba456.jpg]

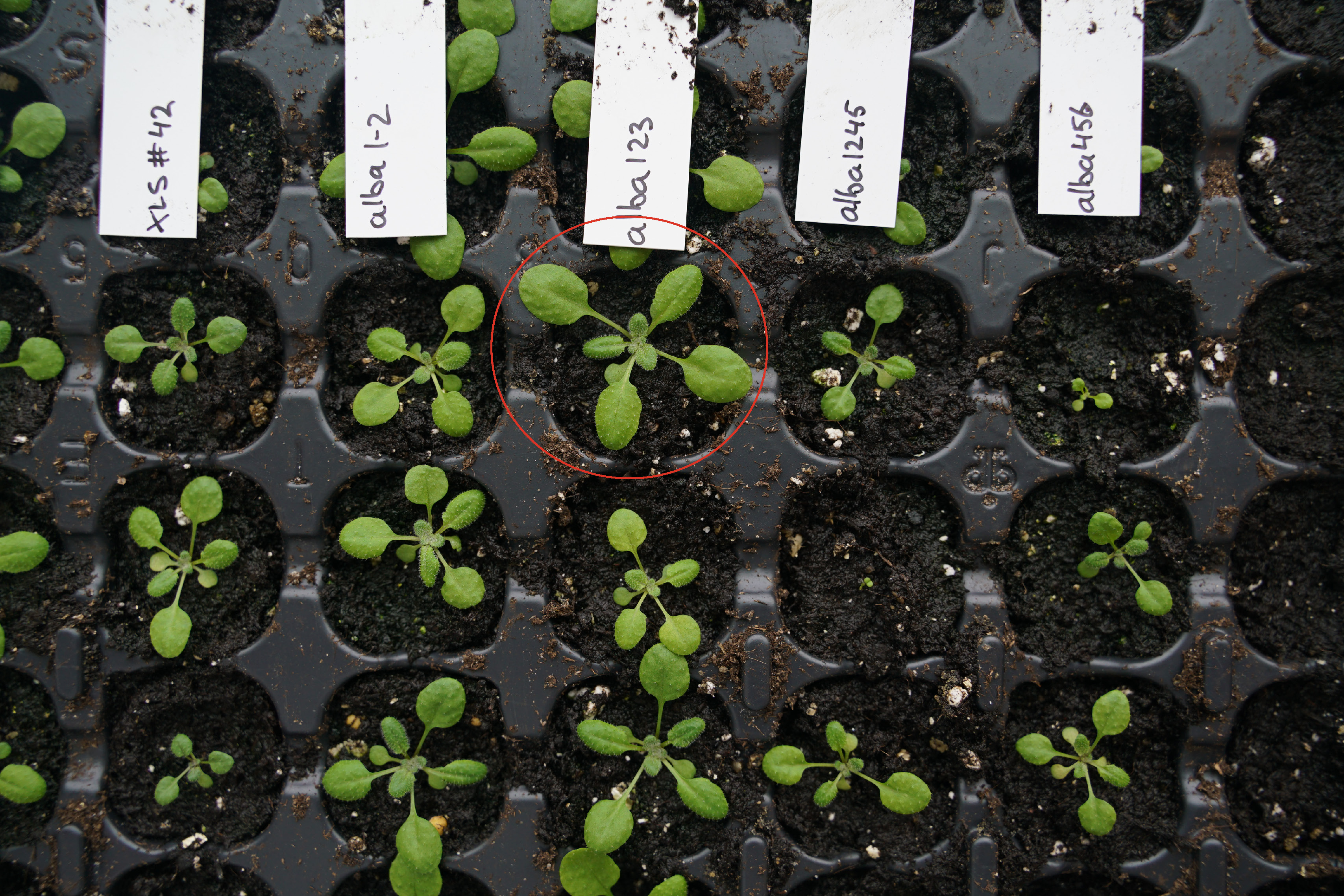

Supplement: Supplementary file 14 — Source data Fig. 7 [file 44318_2024_312_MOESM14_ESM.zip › Source data for Fig 7/7F/17 DAG/alba123.jpg]

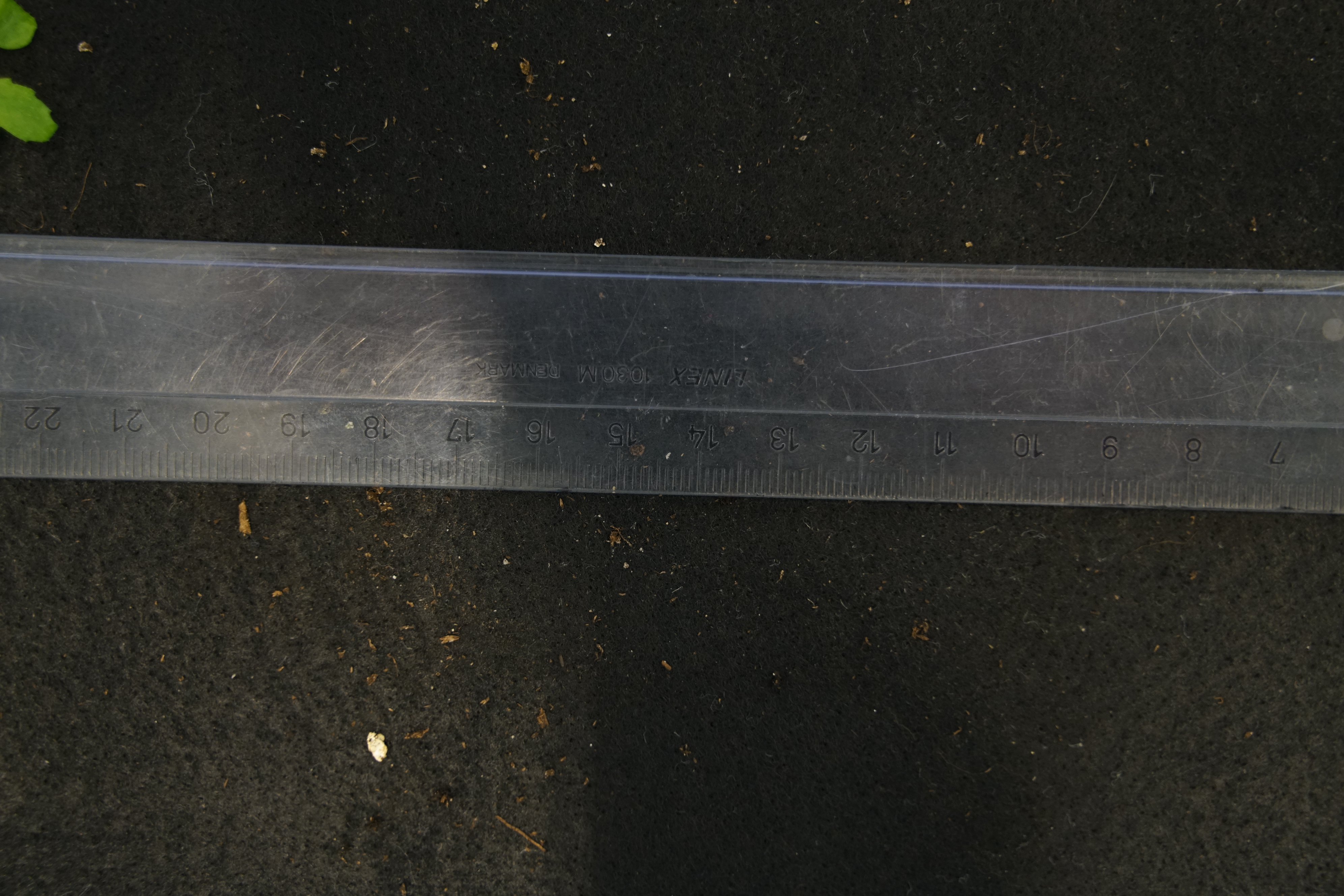

Supplement: Supplementary file 14 — Source data Fig. 7 [file 44318_2024_312_MOESM14_ESM.zip › Source data for Fig 7/7F/26 DAG/Scale.JPG]

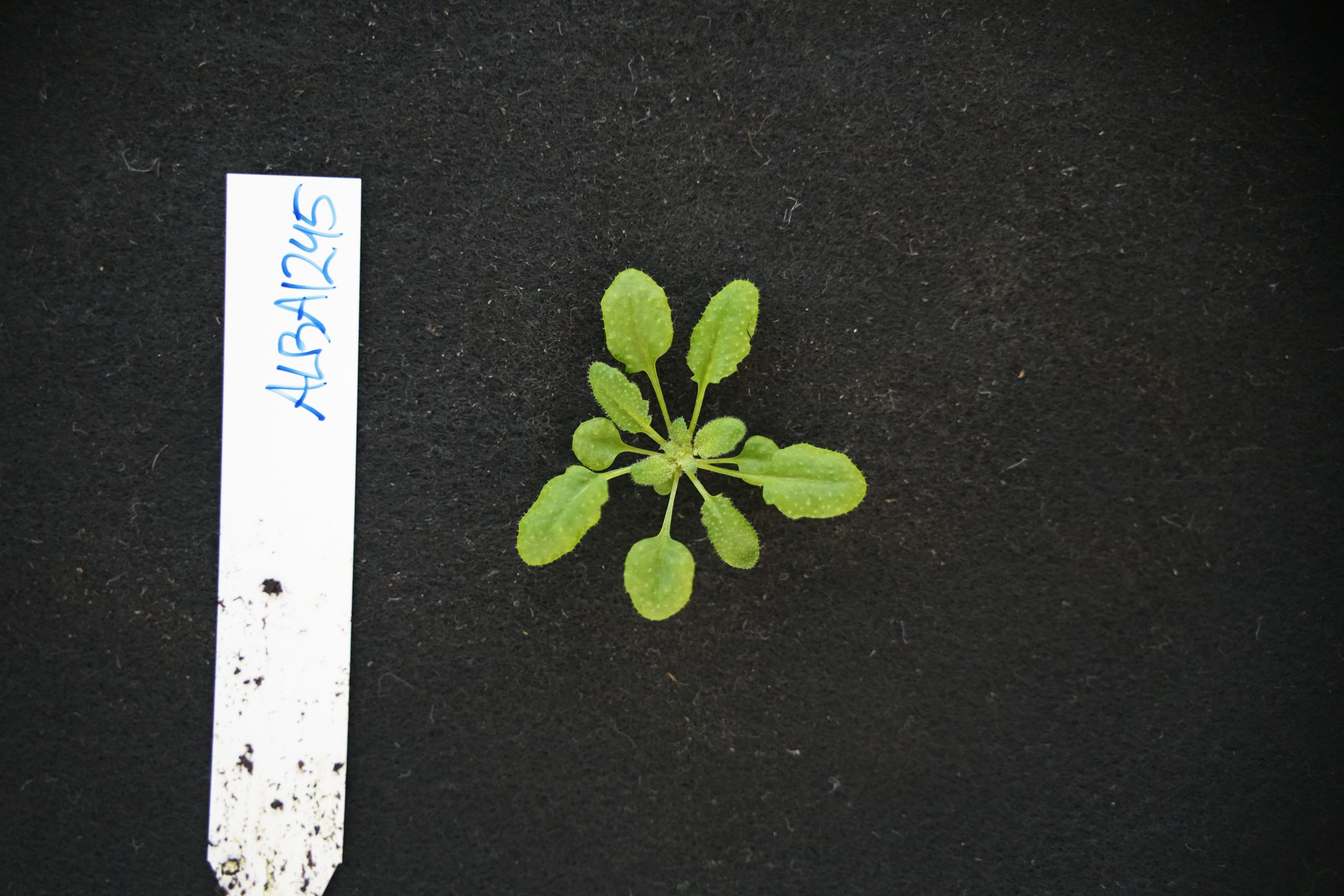

Supplement: Supplementary file 14 — Source data Fig. 7 [file 44318_2024_312_MOESM14_ESM.zip › Source data for Fig 7/7F/26 DAG/alba1245.JPG]

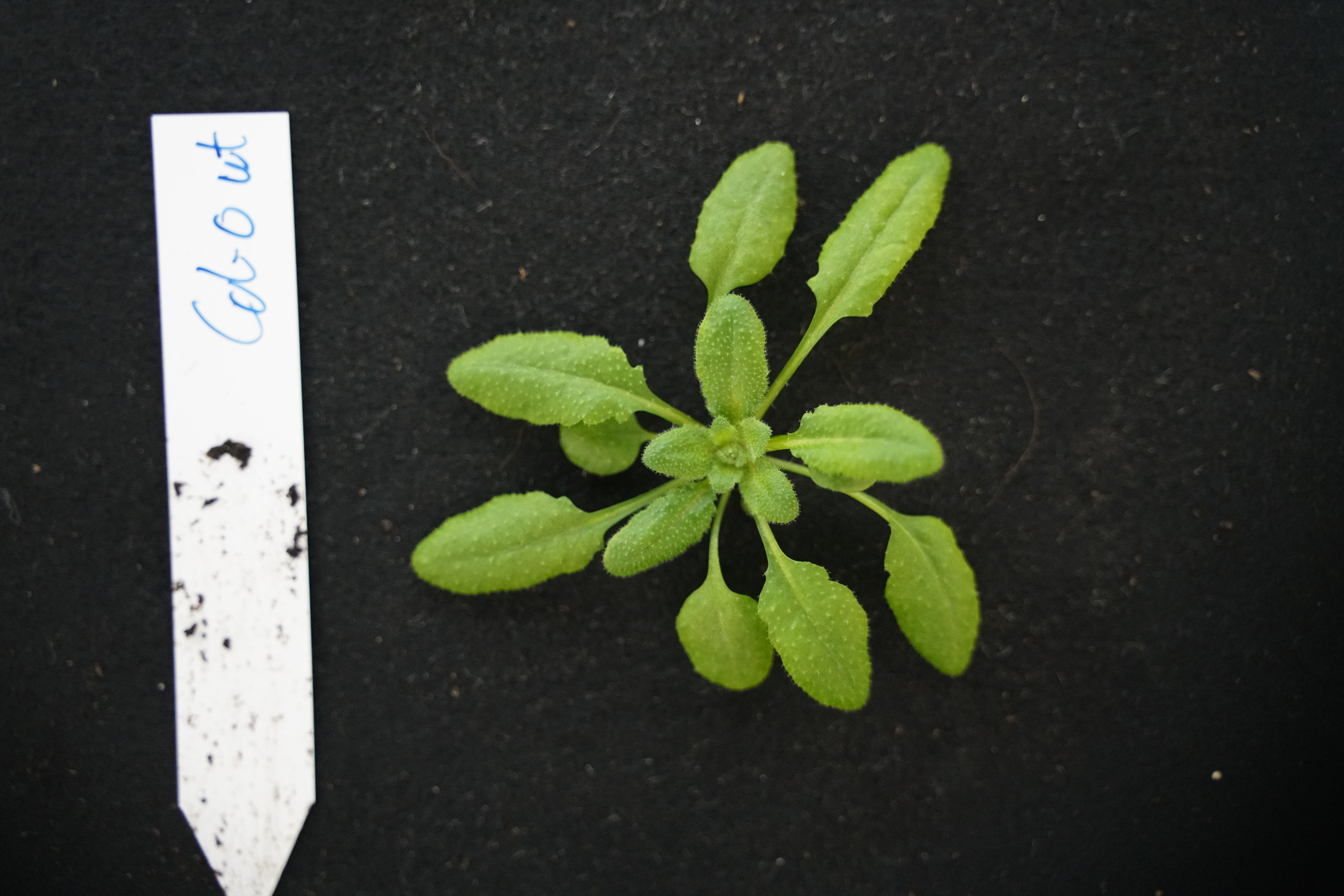

Supplement: Supplementary file 14 — Source data Fig. 7 [file 44318_2024_312_MOESM14_ESM.zip › Source data for Fig 7/7F/26 DAG/Col-0 WT.JPG]

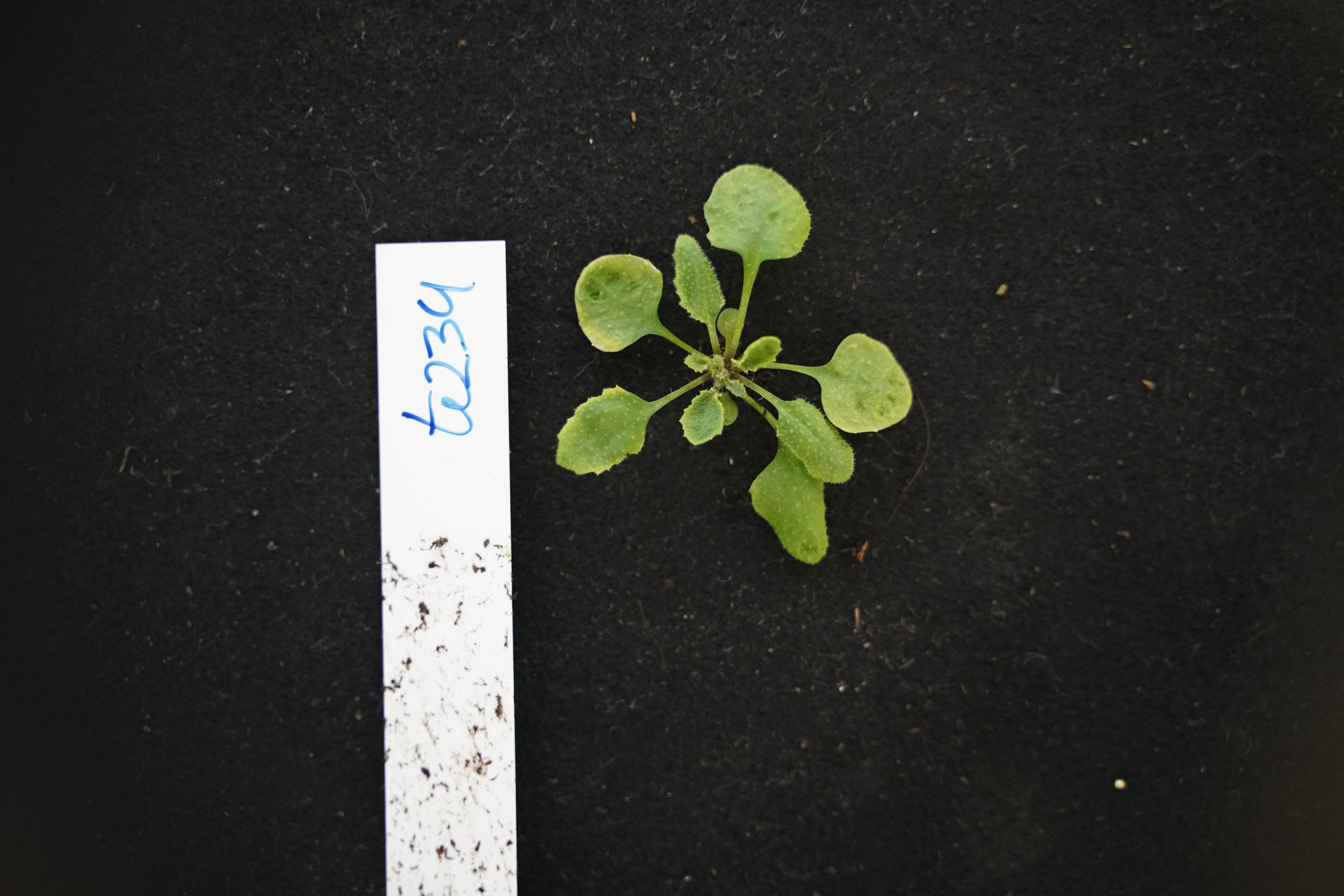

Supplement: Supplementary file 14 — Source data Fig. 7 [file 44318_2024_312_MOESM14_ESM.zip › Source data for Fig 7/7F/26 DAG/te234.JPG]

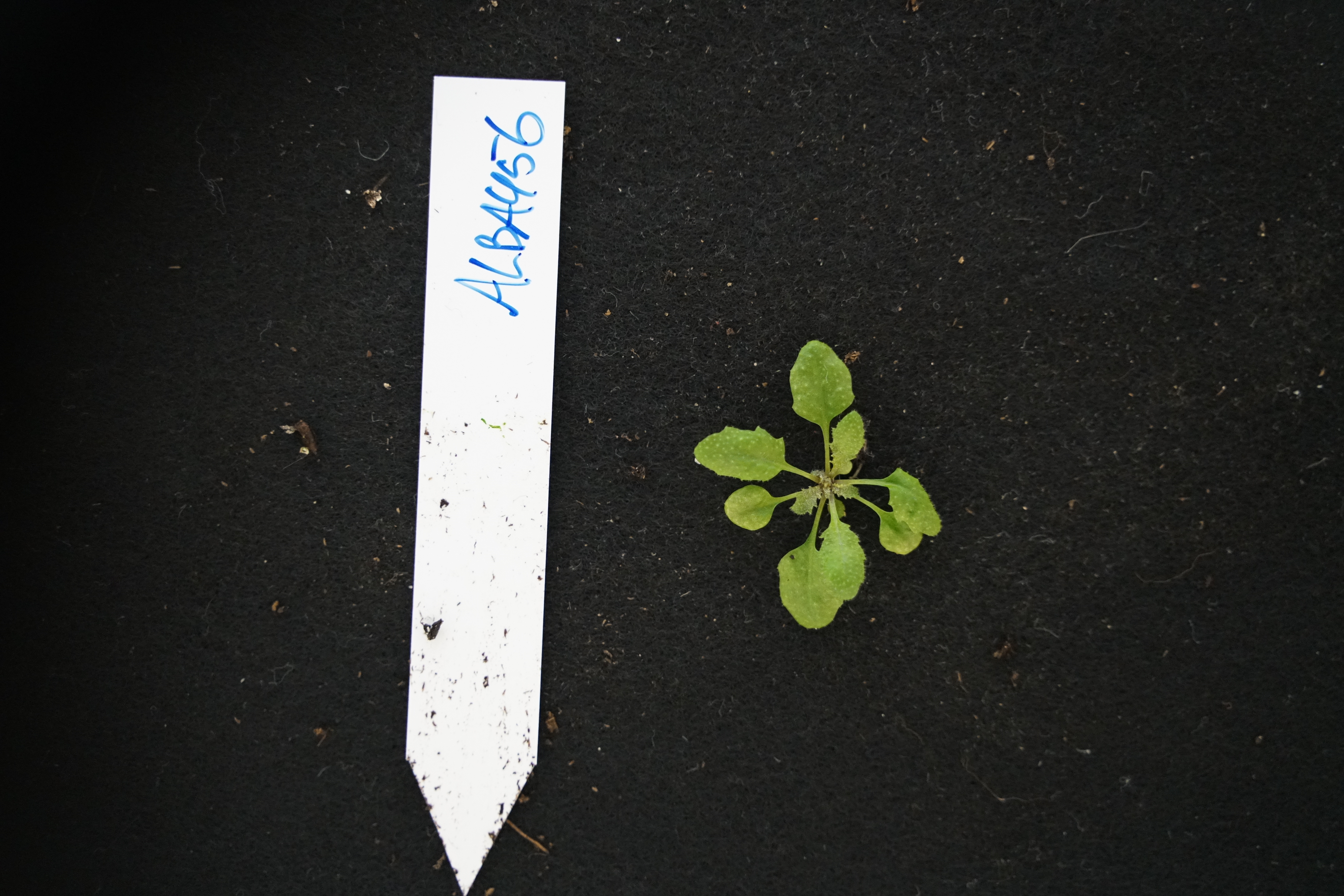

Supplement: Supplementary file 14 — Source data Fig. 7 [file 44318_2024_312_MOESM14_ESM.zip › Source data for Fig 7/7F/26 DAG/alba456.JPG]

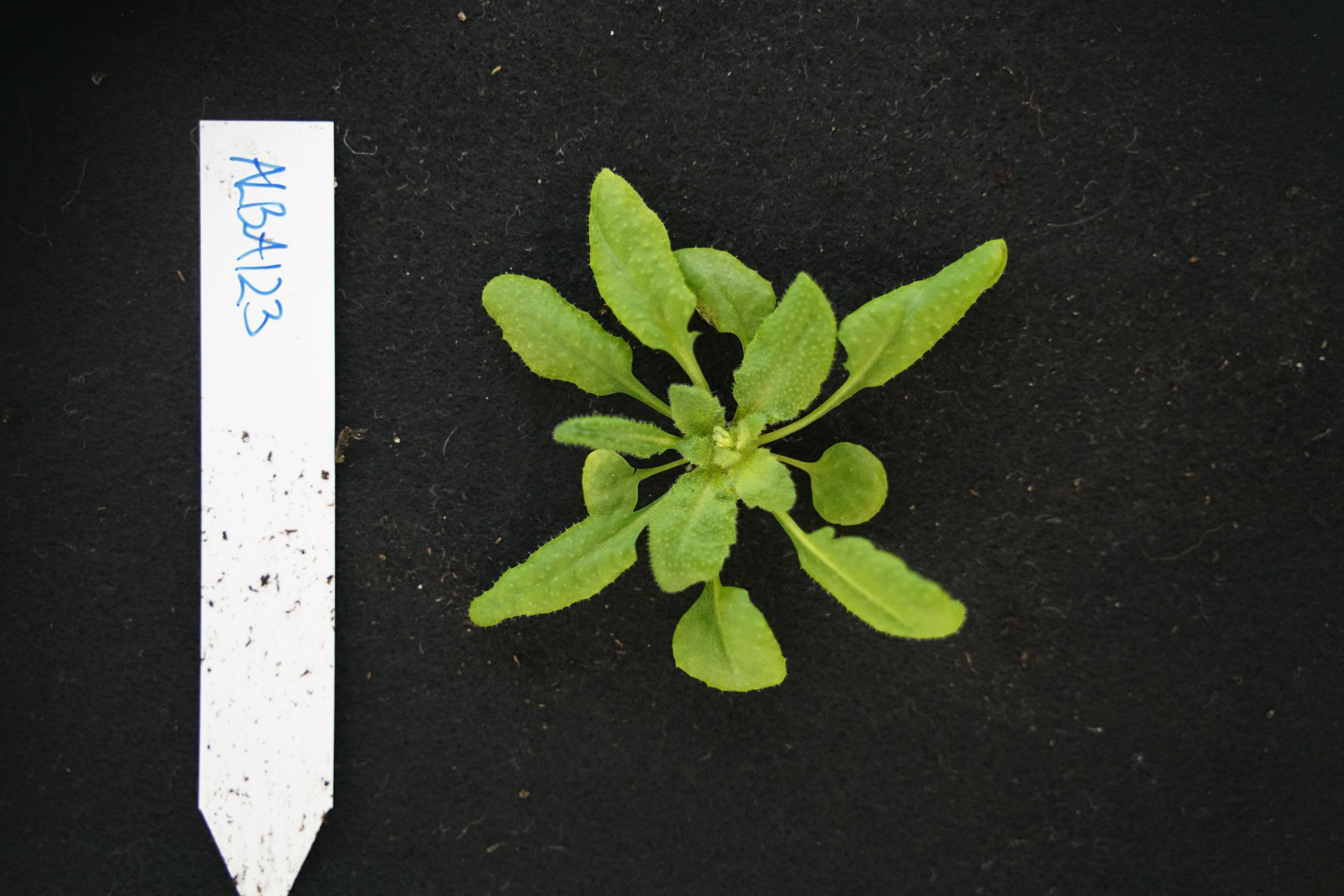

Supplement: Supplementary file 14 — Source data Fig. 7 [file 44318_2024_312_MOESM14_ESM.zip › Source data for Fig 7/7F/26 DAG/ALBA123.JPG]

### Source data for Appendix Fig S2A

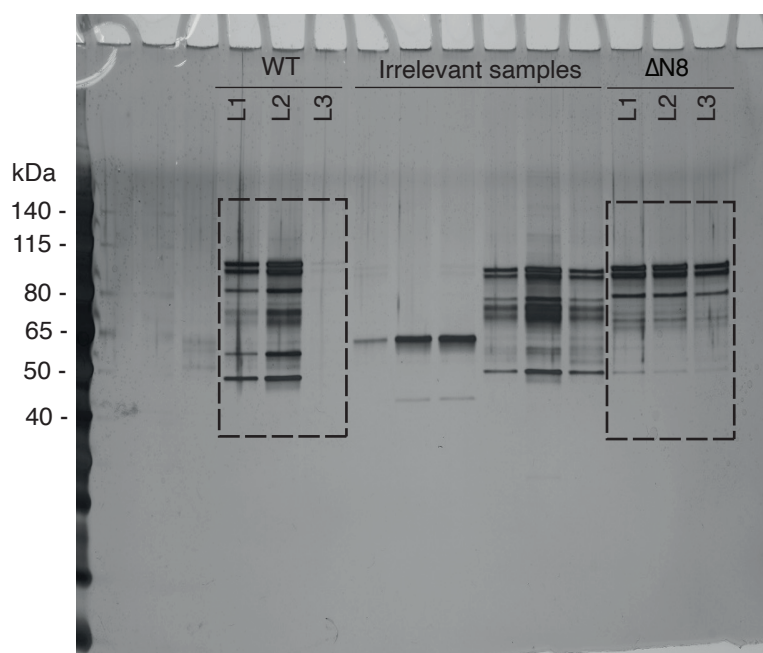

Supplement: Supplementary file 15 — EV and Appendix Figure Source Data [file 44318_2024_312_MOESM15_ESM.zip › Source data for Expanded View and Appendix/Appendix Fig S2/S2A/Silver staining.pdf]

Source data for Appendix Fig S2B

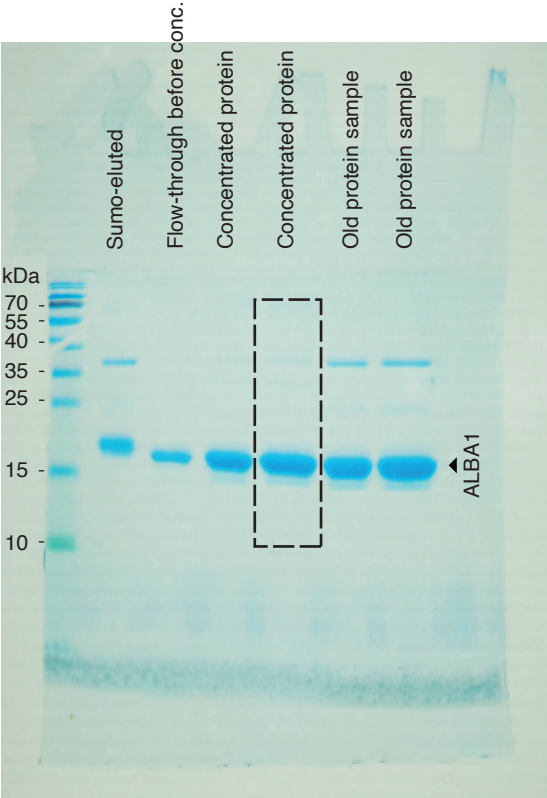

Coomassie

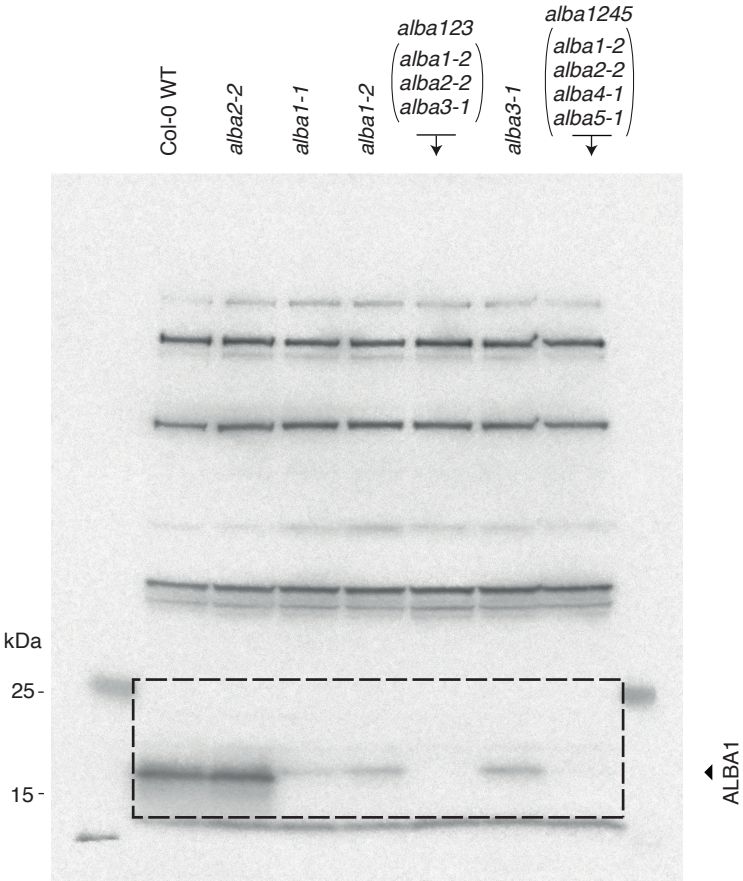

$\alpha$ -ALBA1

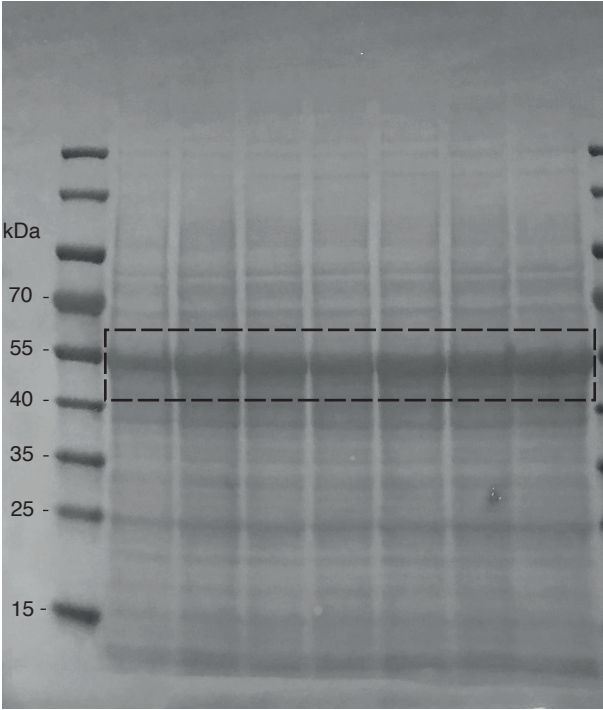

Ponceau

Supplement: Supplementary file 15 — EV and Appendix Figure Source Data [file 44318_2024_312_MOESM15_ESM.zip › Source data for Expanded View and Appendix/Appendix Fig S2/S2B/Coomassie and western blot.pdf]

Source data for Appendix Fig S2D

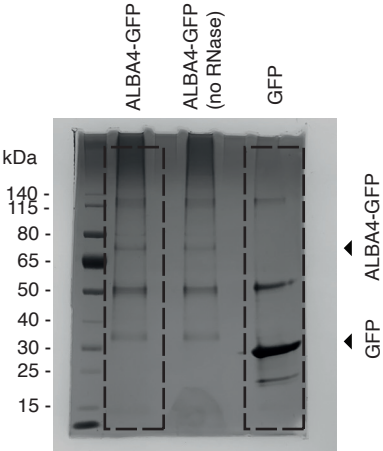

Supplement: Supplementary file 15 — EV and Appendix Figure Source Data [file 44318_2024_312_MOESM15_ESM.zip › Source data for Expanded View and Appendix/Appendix Fig S2/S2D/Silver staining.pdf]

Source data for Fig EV1C

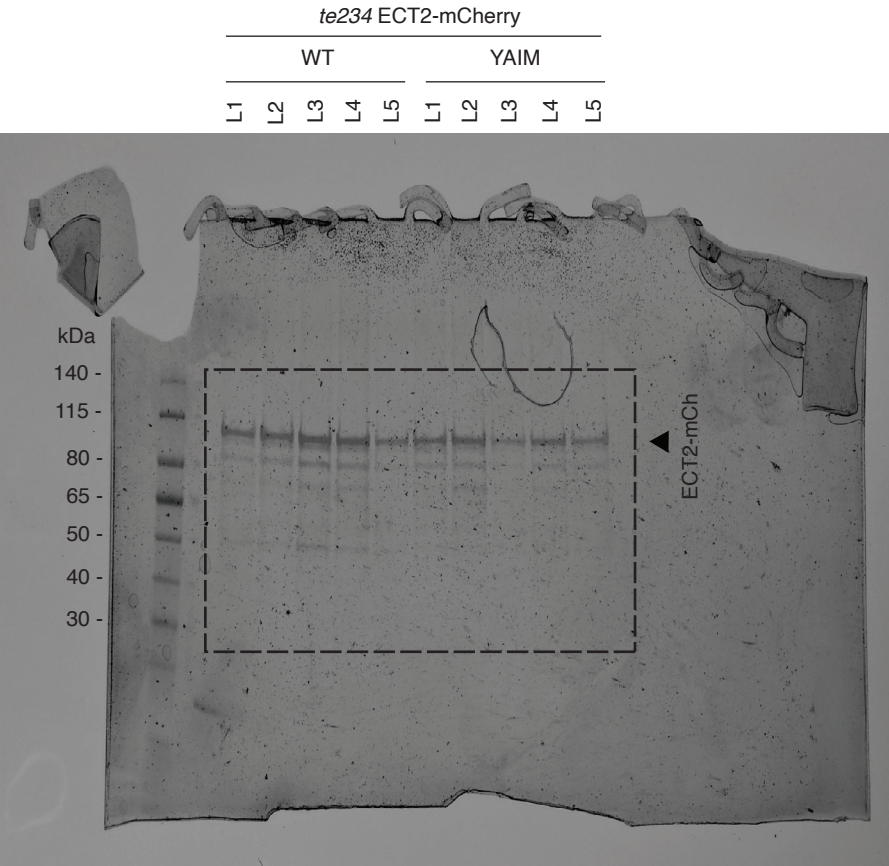

Supplement: Supplementary file 15 — EV and Appendix Figure Source Data [file 44318_2024_312_MOESM15_ESM.zip › Source data for Expanded View and Appendix/Fig EV1/EV1C/Silverstaining RFP IP.pdf]

Source data for Fig EV1D

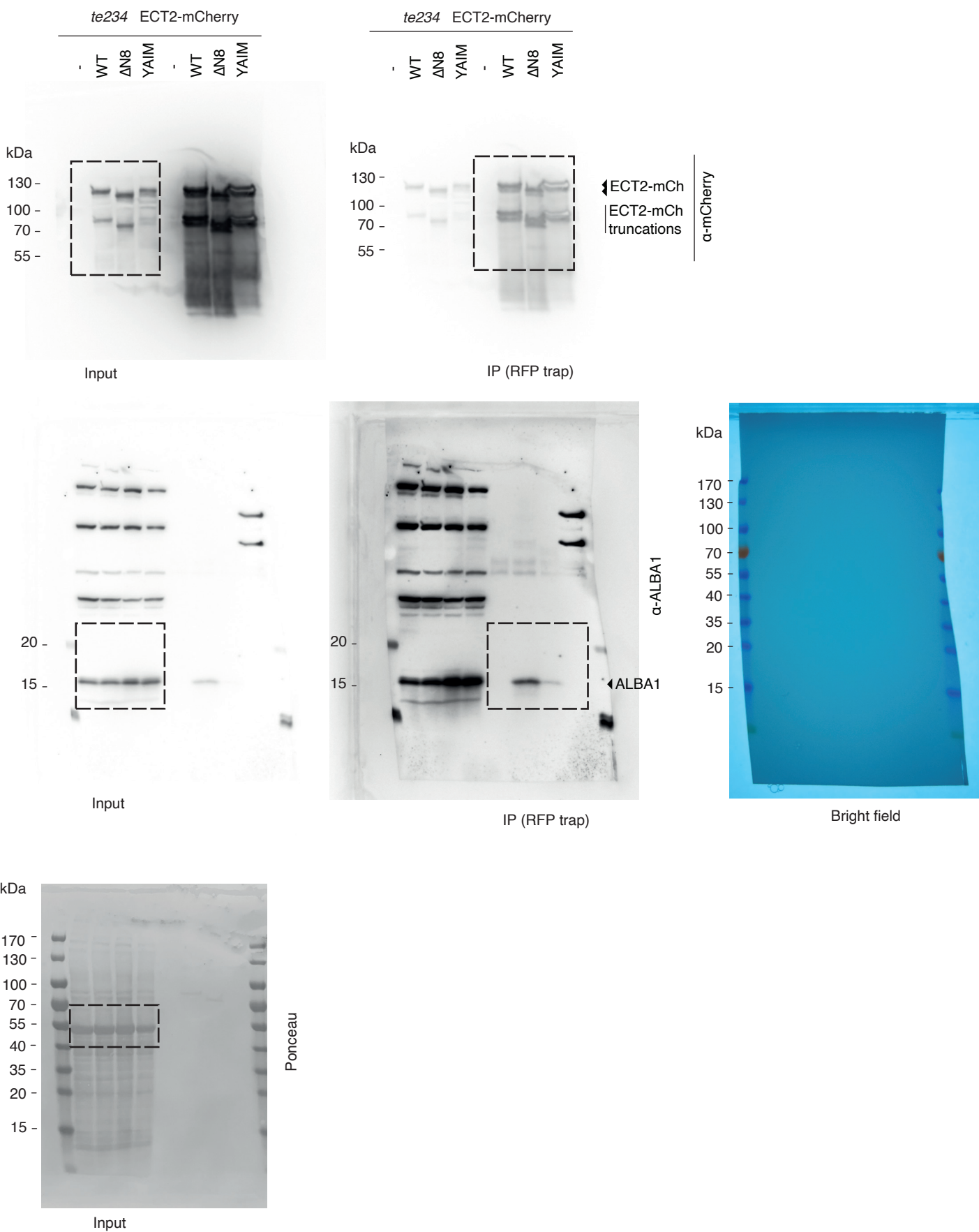

Supplement: Supplementary file 15 — EV and Appendix Figure Source Data [file 44318_2024_312_MOESM15_ESM.zip › Source data for Expanded View and Appendix/Fig EV1/EV1D/Western blot RFP IP.pdf]

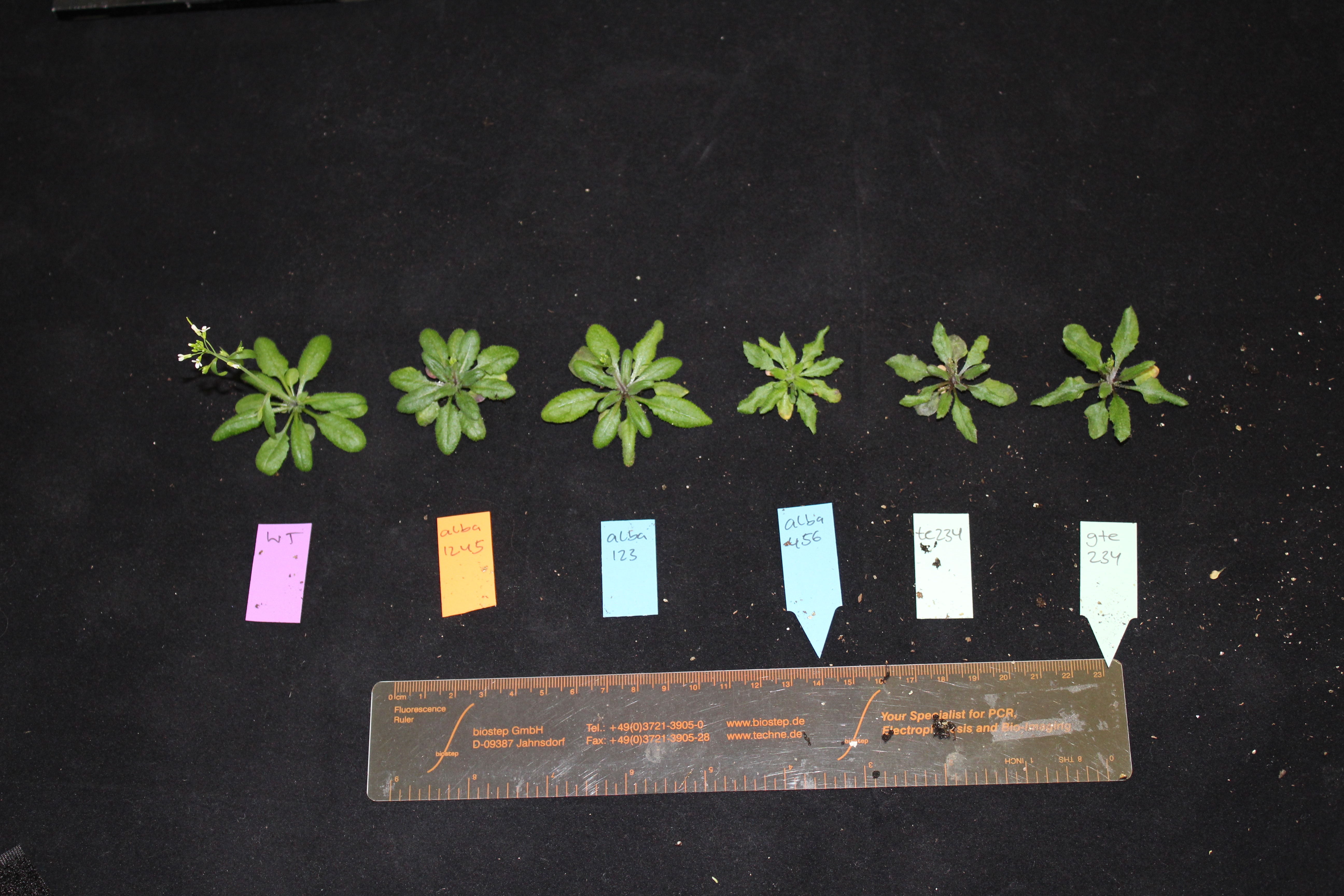

Supplement: Supplementary file 15 — EV and Appendix Figure Source Data [file 44318_2024_312_MOESM15_ESM.zip › Source data for Expanded View and Appendix/Fig EV5/EV5B/Delayed flowering phenotypes.JPG]

Source data for Fig EV5C

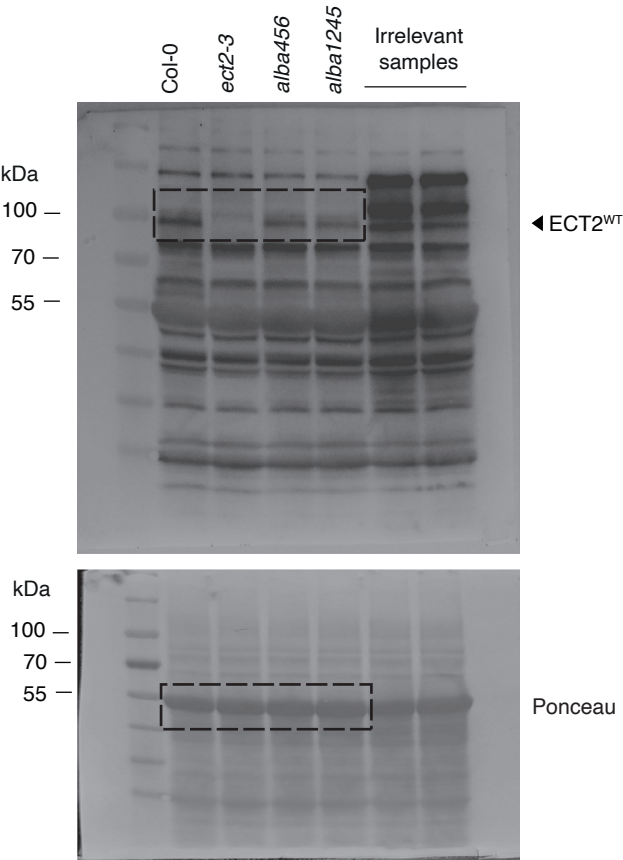

Supplement: Supplementary file 15 — EV and Appendix Figure Source Data [file 44318_2024_312_MOESM15_ESM.zip › Source data for Expanded View and Appendix/Fig EV5/EV5C/ECT2 western.pdf]

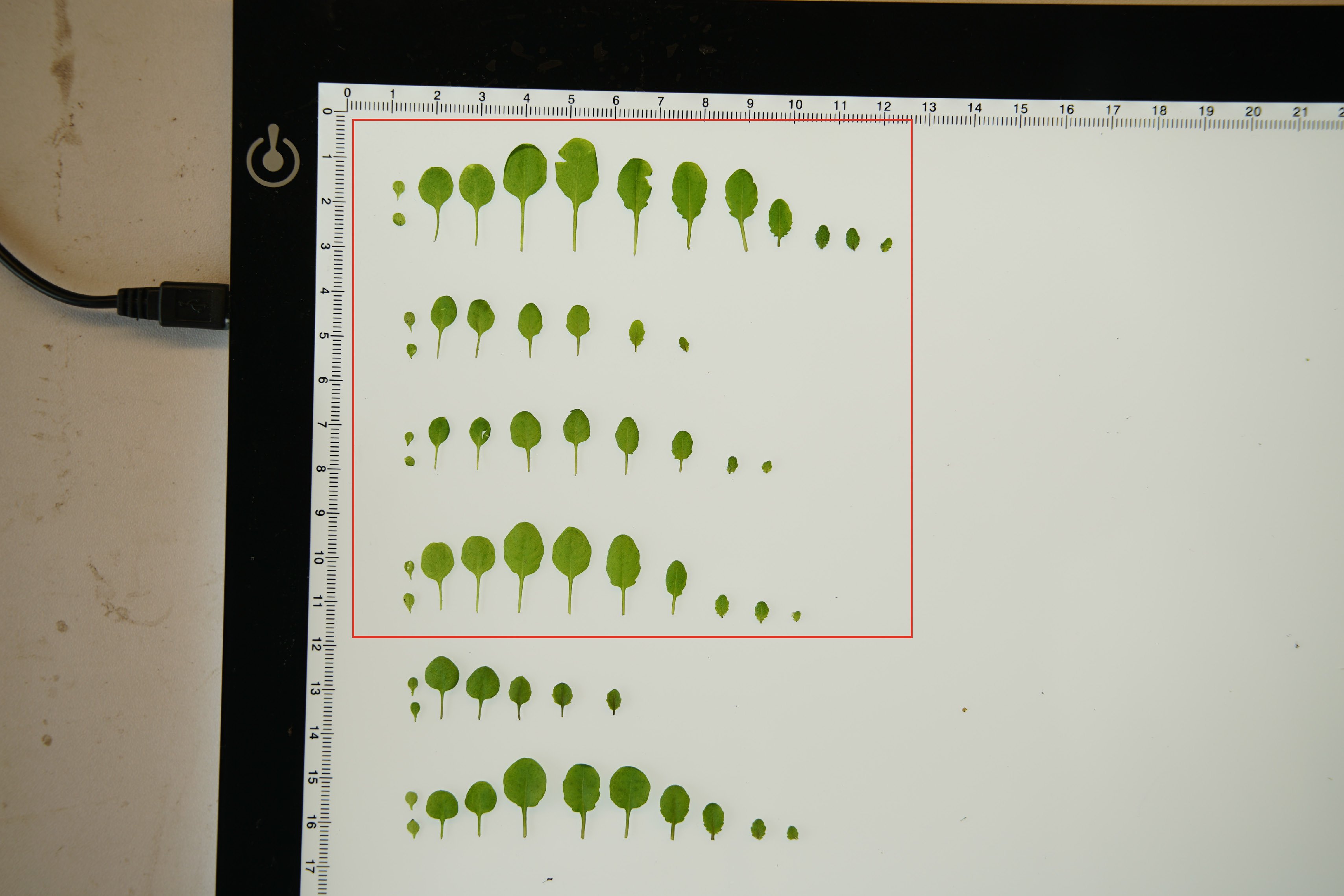

Supplement: Supplementary file 15 — EV and Appendix Figure Source Data [file 44318_2024_312_MOESM15_ESM.zip › Source data for Expanded View and Appendix/Fig EV5/EV5A/Leaf profiles.jpg]

Source data for Appendix Fig S6A

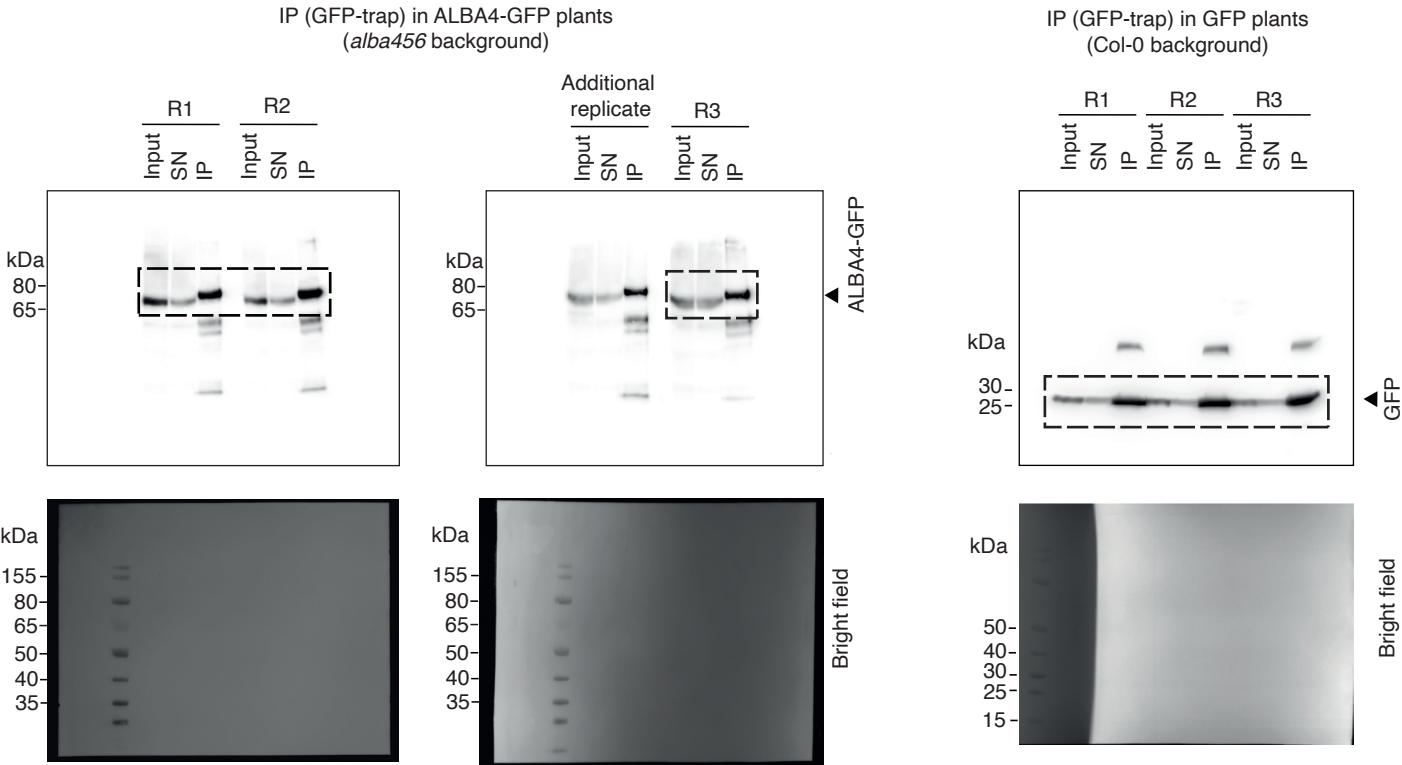

Supplement: Supplementary file 15 — EV and Appendix Figure Source Data [file 44318_2024_312_MOESM15_ESM.zip › Source data for Expanded View and Appendix/Appendix Fig S6/S6A/GFP western.pdf]

Source data for Appendix Fig S6D

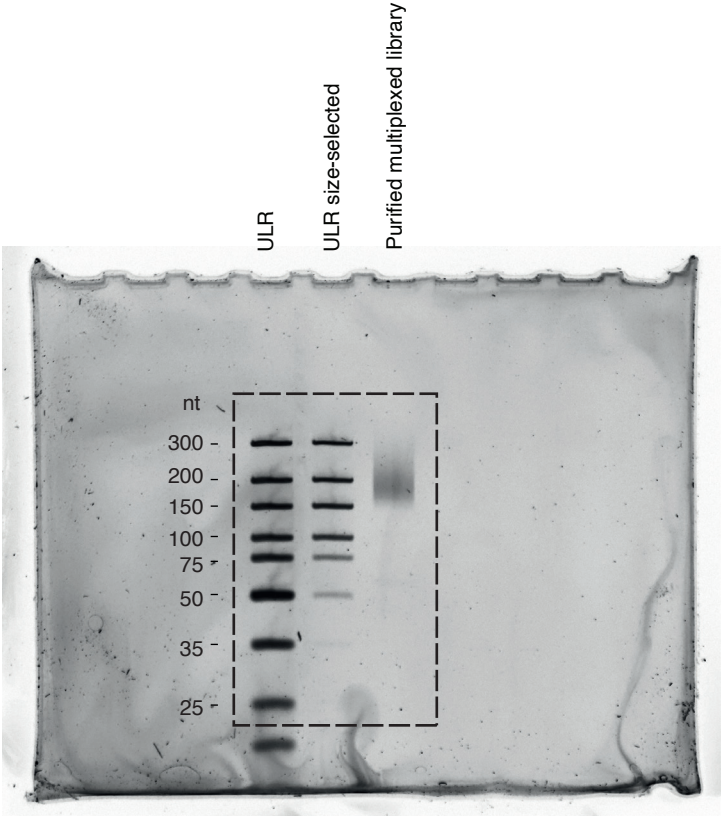

Supplement: Supplementary file 15 — EV and Appendix Figure Source Data [file 44318_2024_312_MOESM15_ESM.zip › Source data for Expanded View and Appendix/Appendix Fig S6/S6D/Purified and multiplexed iCLIP2 libray.pdf]

Source data for Appendix Fig S6C

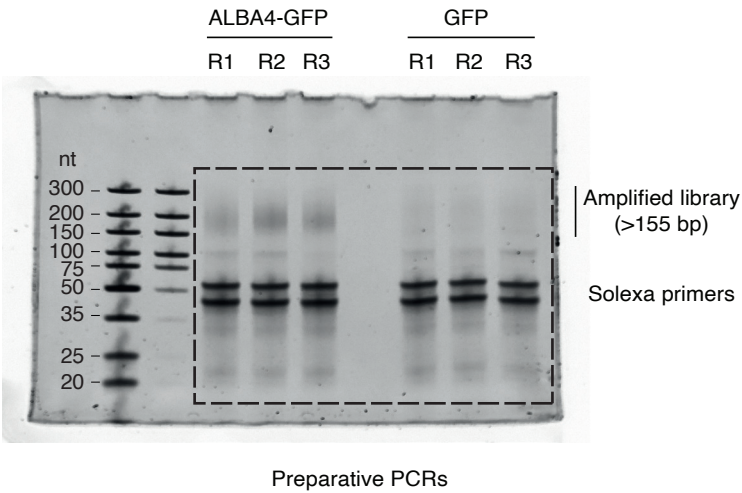

Supplement: Supplementary file 15 — EV and Appendix Figure Source Data [file 44318_2024_312_MOESM15_ESM.zip › Source data for Expanded View and Appendix/Appendix Fig S6/S6C/PCR-amplified iCLIP2 libraries.pdf]

Source data for Appendix Fig S6B

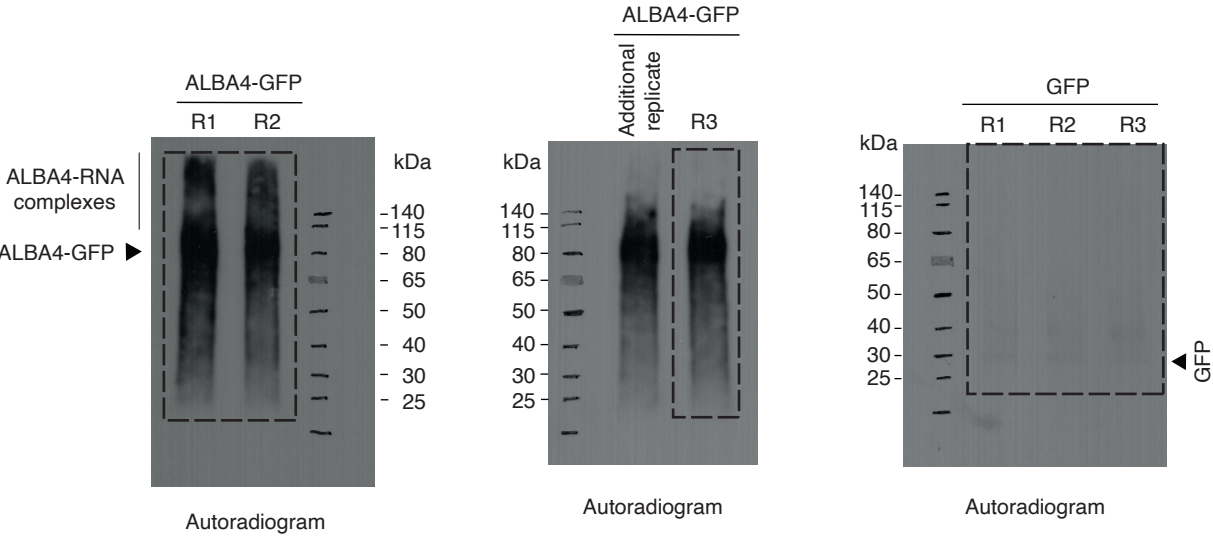

Supplement: Supplementary file 15 — EV and Appendix Figure Source Data [file 44318_2024_312_MOESM15_ESM.zip › Source data for Expanded View and Appendix/Appendix Fig S6/S6B/Autoradiograms ALBA4-GFP and GFP.pdf]

Source data for Fig EV4A

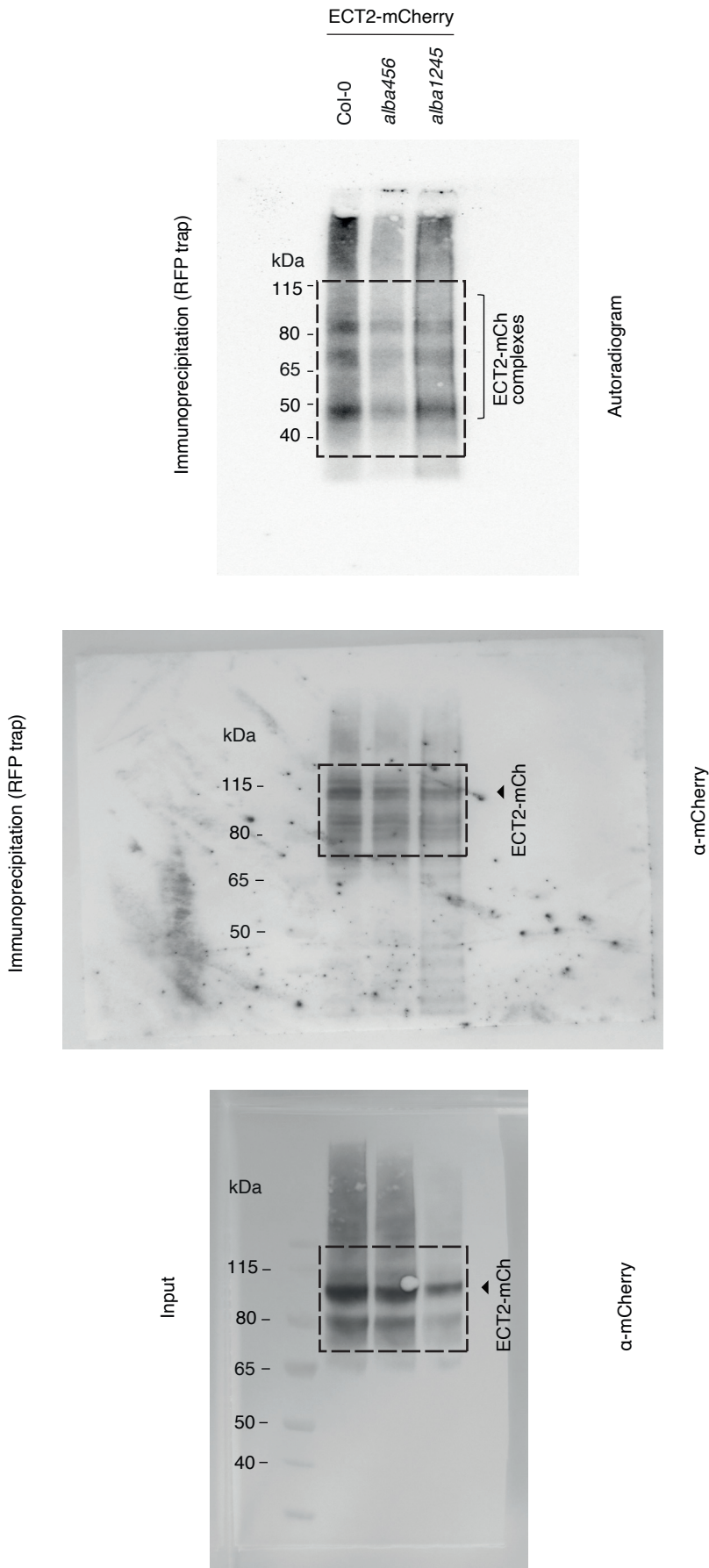

Supplement: Supplementary file 15 — EV and Appendix Figure Source Data [file 44318_2024_312_MOESM15_ESM.zip › Source data for Expanded View and Appendix/Fig EV4/EV4A/Autoradiogram and western after UVXlink and IP.pdf]

Source data for Fig EV4B and EV4D

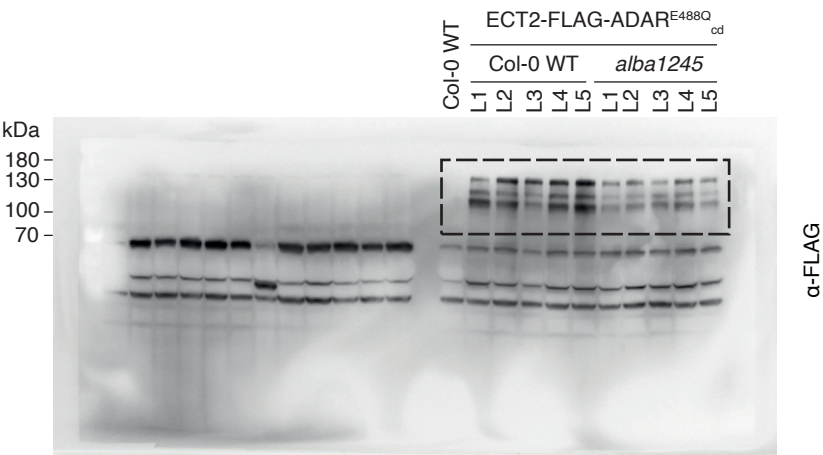

Panel EV4B

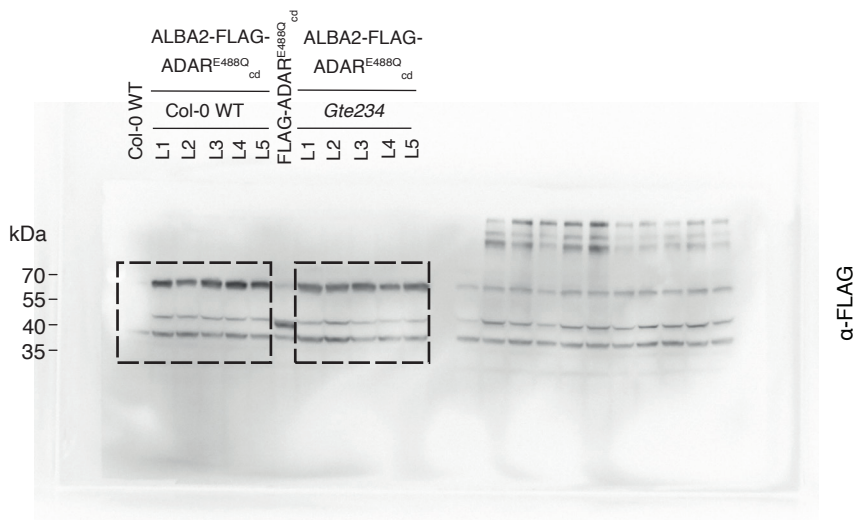

Panel EV4D

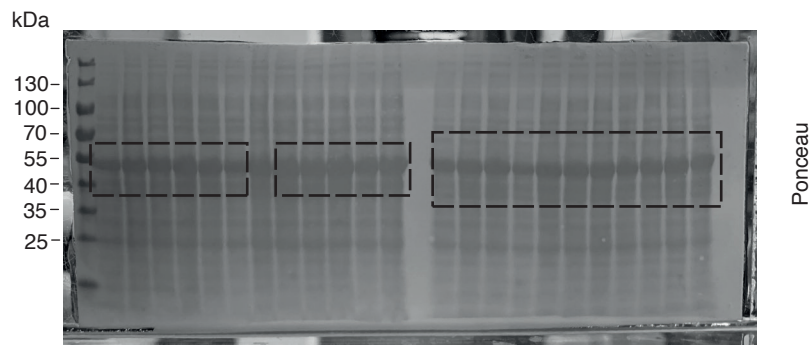

for both panels

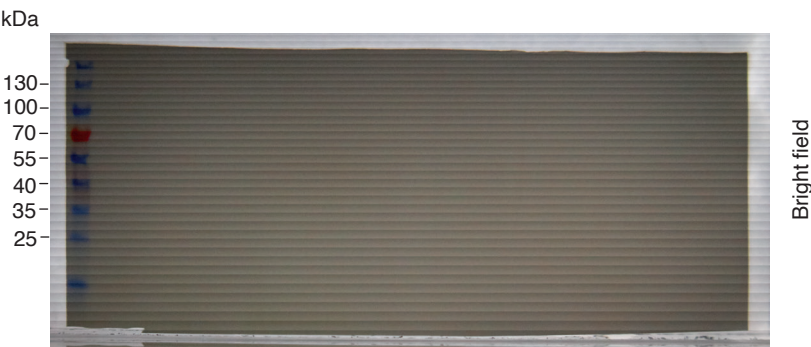

Supplement: Supplementary file 15 — EV and Appendix Figure Source Data [file 44318_2024_312_MOESM15_ESM.zip › Source data for Expanded View and Appendix/Fig EV4/EV4B+D/Western blot ECT2 and ALBA2 HT.pdf]

Source data for Appendix Fig S7B

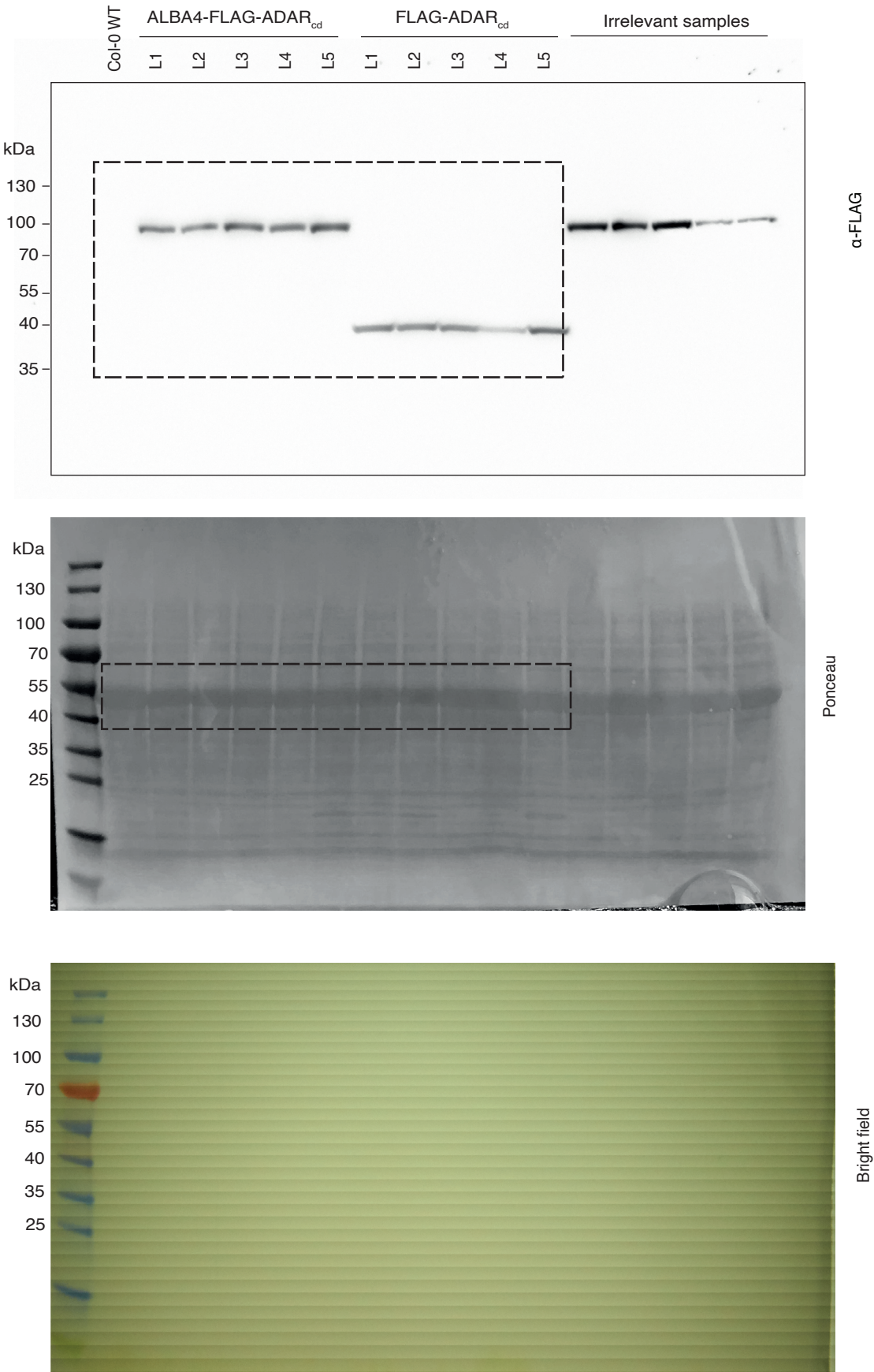

Supplement: Supplementary file 15 — EV and Appendix Figure Source Data [file 44318_2024_312_MOESM15_ESM.zip › Source data for Expanded View and Appendix/Appendix Fig S7/S7B/FLAG western ALBA4 TRIBE.pdf]

Source data for Appendix Fig S7A

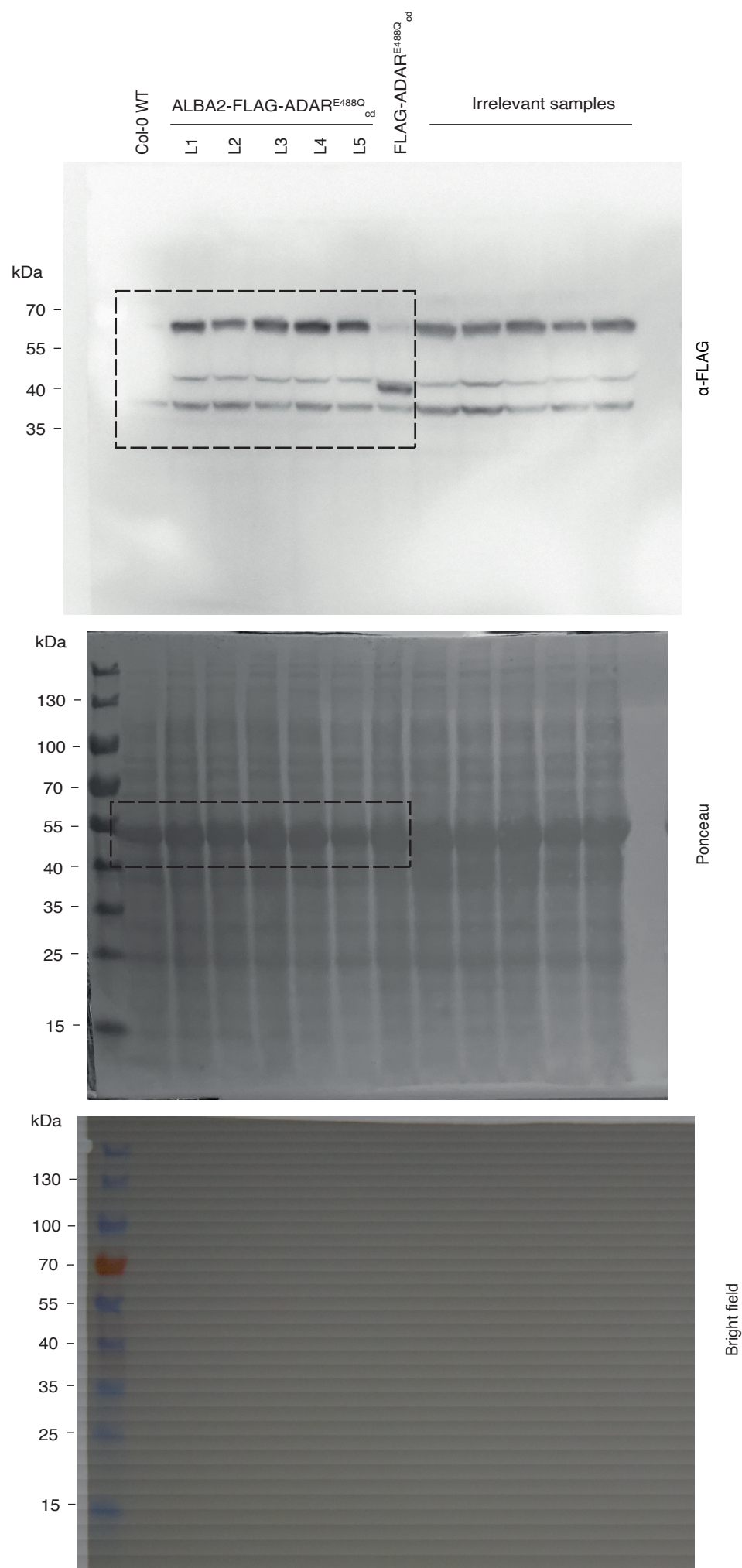

Supplement: Supplementary file 15 — EV and Appendix Figure Source Data [file 44318_2024_312_MOESM15_ESM.zip › Source data for Expanded View and Appendix/Appendix Fig S7/S7A/FLAG western ALBA2 HT.pdf]
