# Supplementary material for: ALBA proteins facilitate cytoplasmic YTHDF-mediated reading of m6A in Arabidopsis
Source: EMBO J. 2024 Nov 29;43(24):6626–55. doi: 10.1038/s44318-024-00312-0 (PMC11649824; doi:10.1038/s44318-024-00312-0)

Source data for Appendix Fig S5D

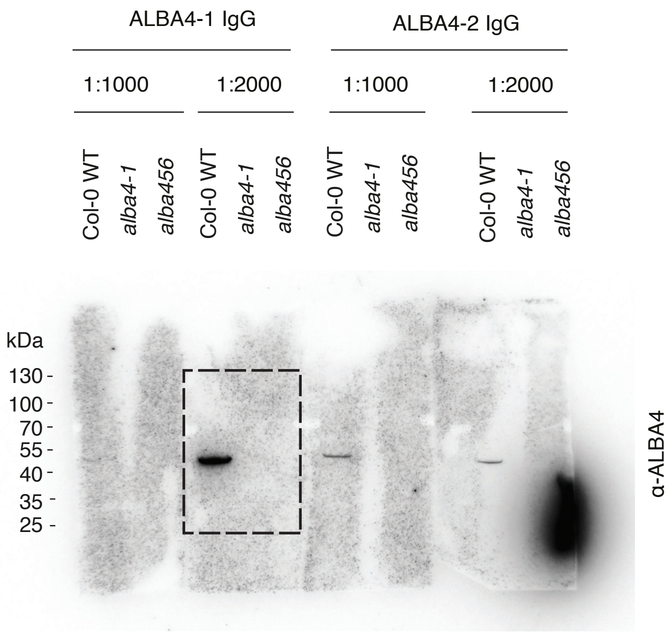

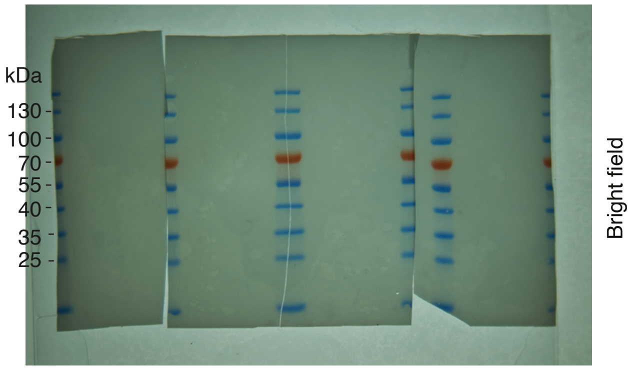

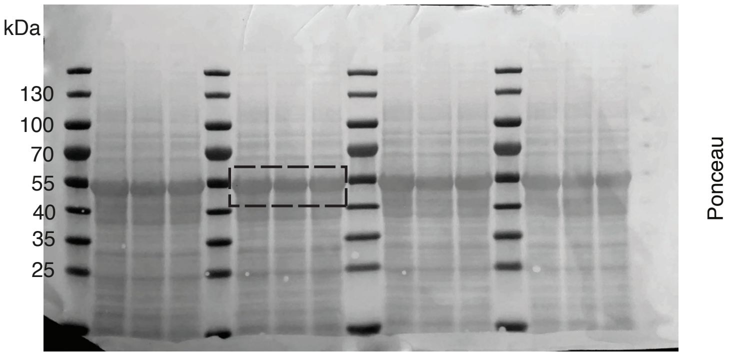

Supplement: Supplementary file 15 — EV and Appendix Figure Source Data [file 44318_2024_312_MOESM15_ESM.zip › Source data for Expanded View and Appendix/Appendix Fig S5/S5D/Western anti-ALBA4.pdf]
