# Supplementary material for: ALBA proteins facilitate cytoplasmic YTHDF-mediated reading of m6A in Arabidopsis
Source: EMBO J. 2024 Nov 29;43(24):6626–55. doi: 10.1038/s44318-024-00312-0 (PMC11649824; doi:10.1038/s44318-024-00312-0)
Supplement: Supplementary file 16 — Expanded View Figures [file 44318_2024_312_MOESM16_ESM.pdf]

# Expanded View Figures

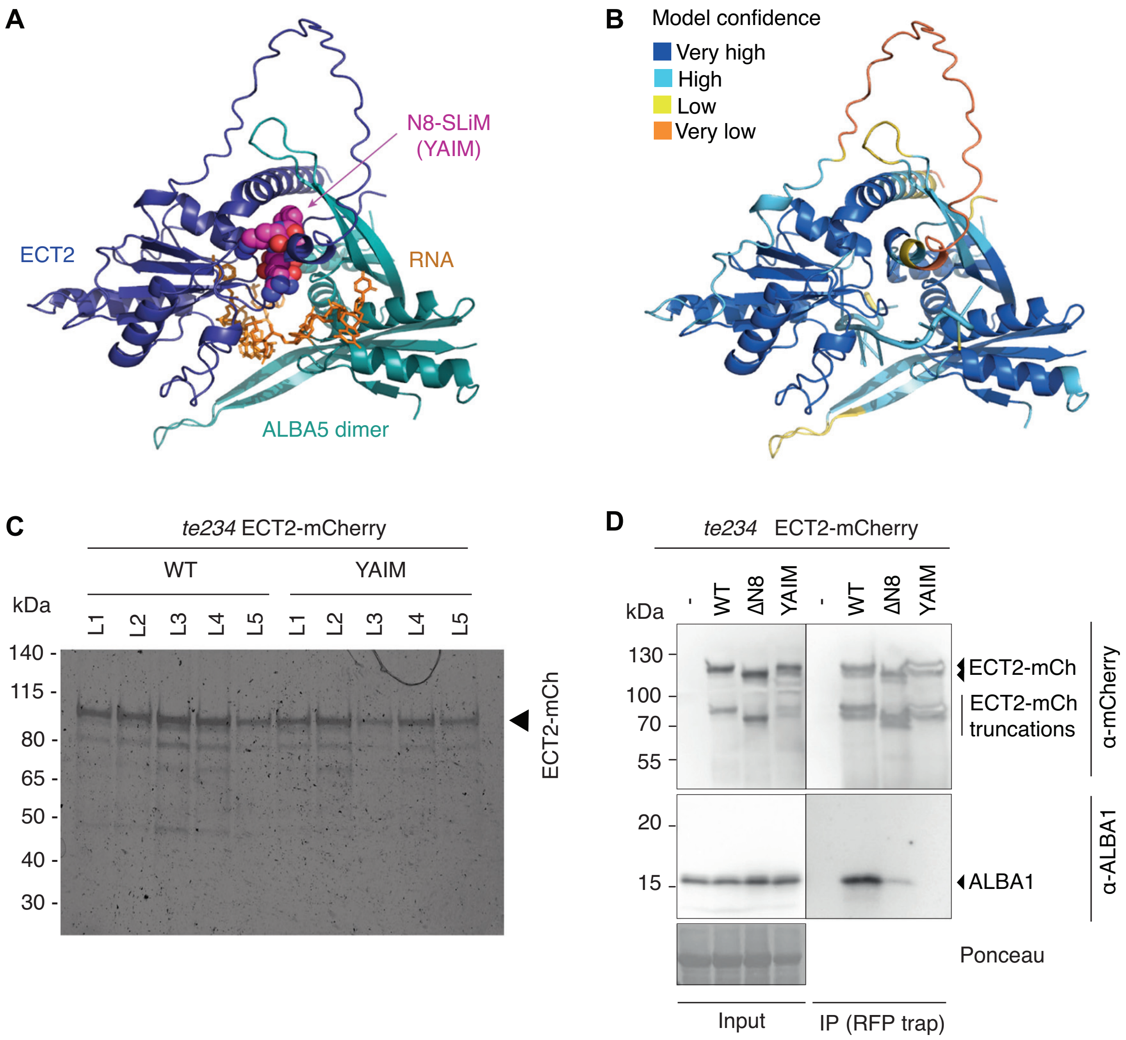


**Figure EV1. The YTH-ALBA Interacting Motif (YAIM) is central for ECT2-ALBA interaction (supporting data).**

(**A**) Alternative view of the AlphaFold3 model shown in Fig. 2B of the complex between ECT2 (YTH domain plus a YAIM-containing fragment of the N-terminal IDR), two ALBA5 subunits (ALBA domains only), and the 10-nt RNA [5′-AAA($m^6$A)CUUCUG-3′]. The YAIM is accentuated in space-fill mode (Magenta, C; Blue, N; Red, O), all other protein elements in cartoon mode, and the RNA in stick mode (supports Fig. 2B). (**B**) Same view of the model as in panel (**A**), but colored according to the predicted local distance difference test (pLDDT) score calculated by Alphafold3 to indicate model confidence on a local per-residue basis (Abramson et al, 2024) (supports Fig. 2C). (**C**) Silver staining of aliquots of immunopurified fractions used for LS-MS/MS analysis of differential enrichment in ECT2-mCherry and ECT2$^{YAIM}$-mCherry purifications (supports Fig. 2I). (**D**) Co-immunoprecipitation analysis of the ECT2-ALBA1 interaction. Nine-day-old seedlings from three independent transgenic lines expressing each ECT2-mCherry variant were pooled prior to mCherry immunoprecipitation and analysis by western blot. Source data are available online for this figure.


© The Author(s)

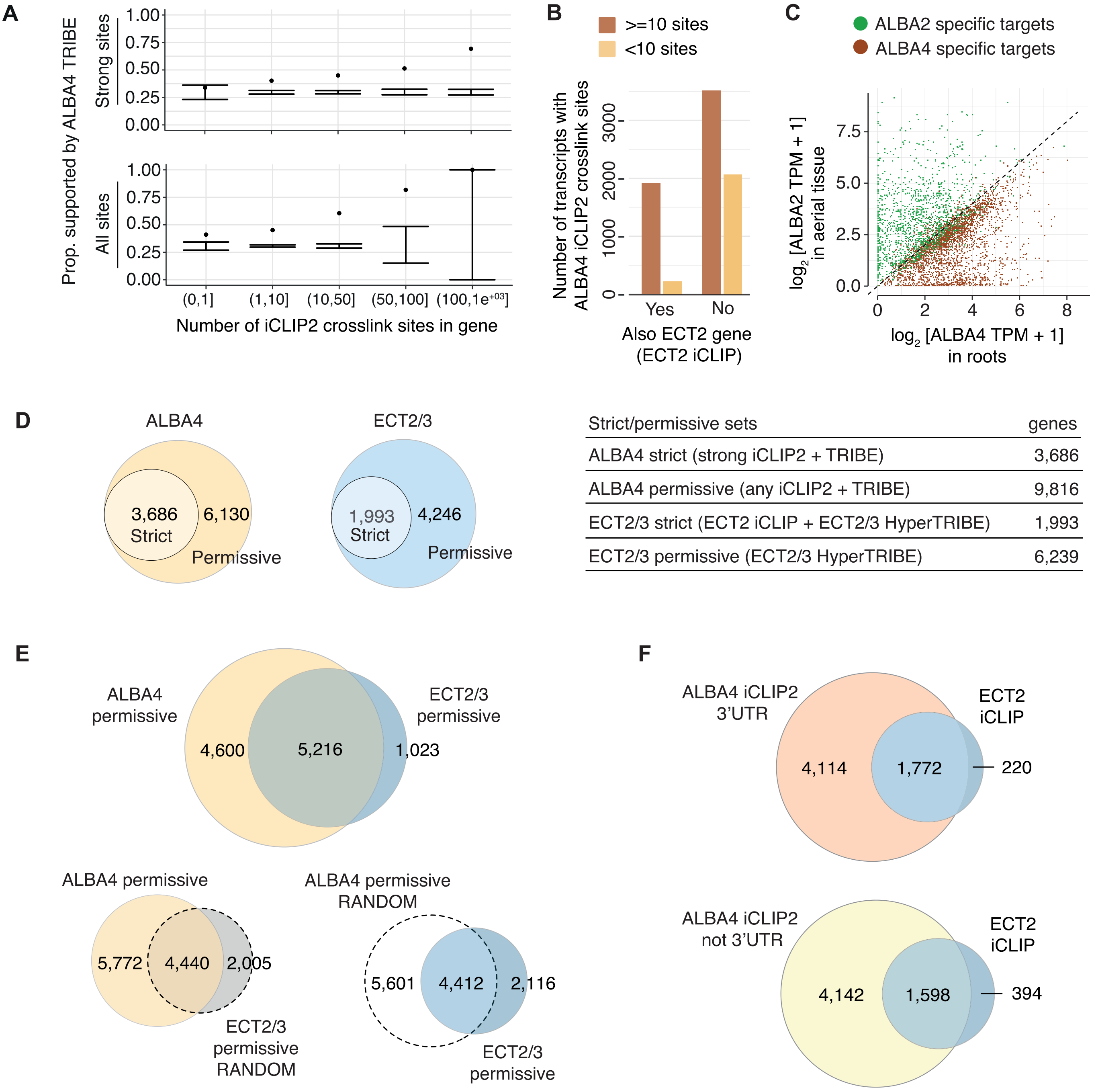


**Figure EV2. Analysis of ALBA2 and ALBA4 target sets (supporting data).**

(**A**) Proportion of genes supported by ALBA4-TRIBE according to the number of iCLIP2 crosslink sites in the gene. Points represent true proportions, and intervals represent background distribution based on sampling genes of similar expression levels to the target genes from ALBA4 iCLIP2. Error bars are based on 2.5 and 97.5% quantiles and $n = 177$, 2282, 2855, 1299, 1132 is the number of iCLIP2 crosslink sites considered in each group (from left to right). The top panel represents the strong set of replicated ALBA4 iCLIP2 peaks and bottom panel represents the full set of replicated ALBA4 iCLIP2 peaks. (**B**) Support of ALBA4 iCLIP2 crosslink site-containing genes according to whether the gene is also supported by ECT2 iCLIP crosslink site-containing genes. (**C**) Scatter plot comparing expression for targets specific to either ALBA4-TRIBE (roots) or ALBA2 HyperTRIBE (aerial tissues). Values represent the averaged $\log_2(\text{TPM} + 1)$ values across the Col-0 WT lines in each experiment. (**D**) Table showing defined strict and permissive gene sets for ECT2/3 and ALBA4. Venn diagrams provide visual representations of the ECT2/3 and ALBA4 strict and permissive gene sets. (**E**) Venn diagram for overlap between ALBA4 permissive and ECT2/3 permissive genes. Smaller Venn diagrams indicate overlap if either the ALBA4 or ECT2/3 permissive target sets were a randomly selected set of genes with a similar expression distribution to the true sets. (**F**) Venn diagrams for overlap between ECT2 iCLIP-derived target genes (strict) and ALBA4 iCLIP2 genes, where the ALBA4 set either contains or does not contain crosslink sites in its 3′-UTR.

© The Author(s)

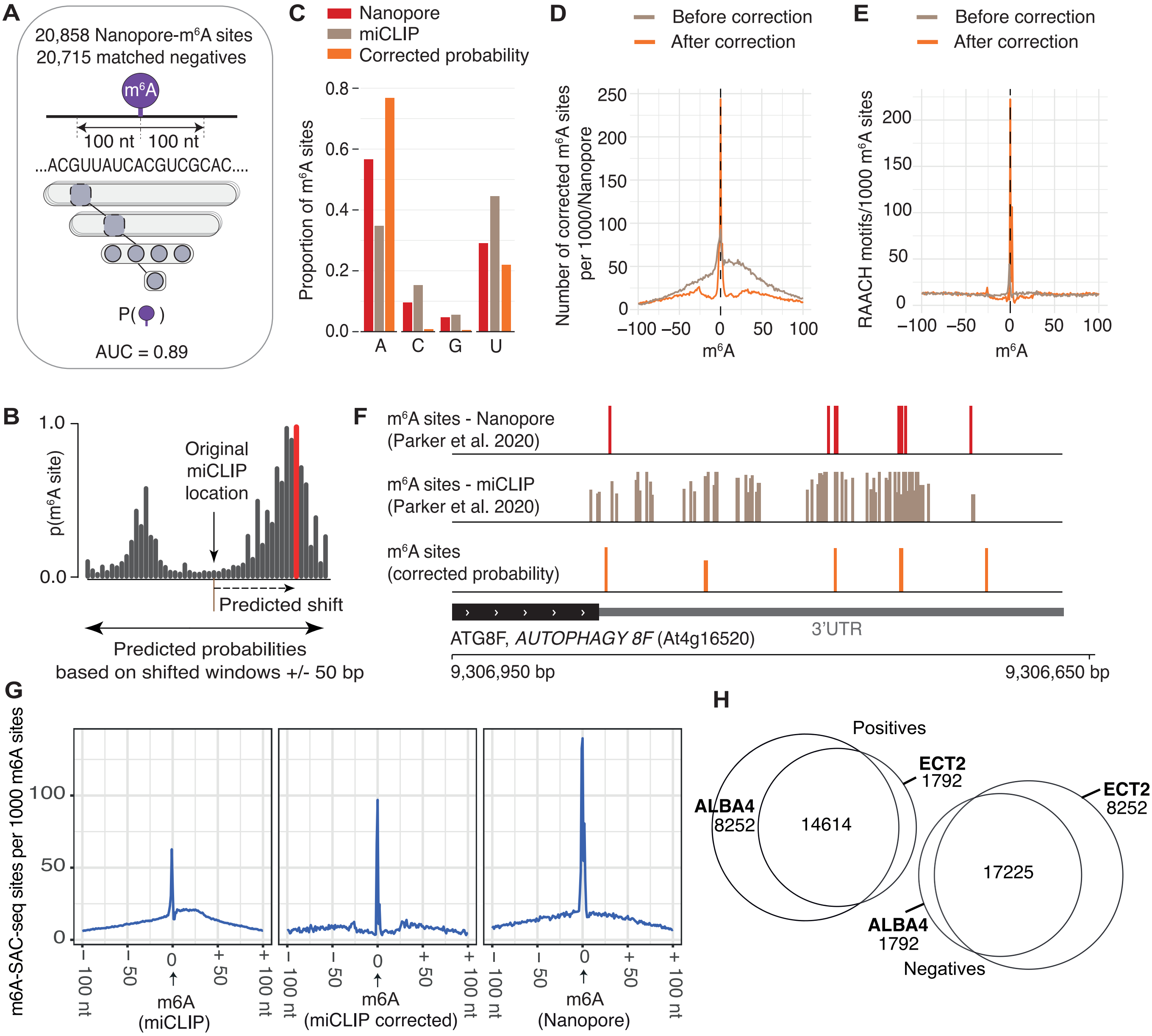


**Figure EV3. A deep learning model to derive an augmented set of single-nucleotide-resolution m⁶A sites by integration of miCLIP and Nanopore data.**

(**A**) Strategy for the m⁶A deep learning model. Each Nanopore m⁶A site was paired with a location-matched negative (see Methods for definition of location-matching), and sequences ±100 bp from the site were extracted and used as input to a convolutional neural network tasked with predicting the presence or absence of m⁶A in the center of the input sequence. AUC area under the curve. (**B**) Strategy for correcting the exact position of m⁶A sites based on the deep learning model. For each site and all individual positions within a ±50 bp window, a ±100 bp sequence was extracted and used as input to the m⁶A-network to predict the presence of m⁶A. The position with the strongest prediction was chosen as the corrected position for that site. (**C**) Reference base distribution of m⁶A sites as determined by Nanopore, miCLIP, or the corrected positions. (**D**) Enrichment of nanopore-determined m⁶A sites around the augmented set of m⁶A positions, before and after correction. (**E**) Enrichment of RRACH motifs per 1000 augmented m⁶A sites, before and after correction. (**F**) IGV view of a representative transcript, *AUTOPHAGY 8F* (At4g16520), showing the positions of m⁶A sites experimentally determined by either nanopore or miCLIP, and the positions resulting from the correction. (**G**) Enrichment of m⁶A sites identified by m⁶A-SAC-seq (Wang et al, 2024) around miCLIP sites before and after correction, and around nanopore-derived sites. (**H**) Annotation of m⁶A sites according to ECT2 and ALBA4 crosslink sites within 100 nt. Venn diagrams show overlap between the two proteins for both positives (bound) and negatives (non-bound) separately.

 © The Author(s)

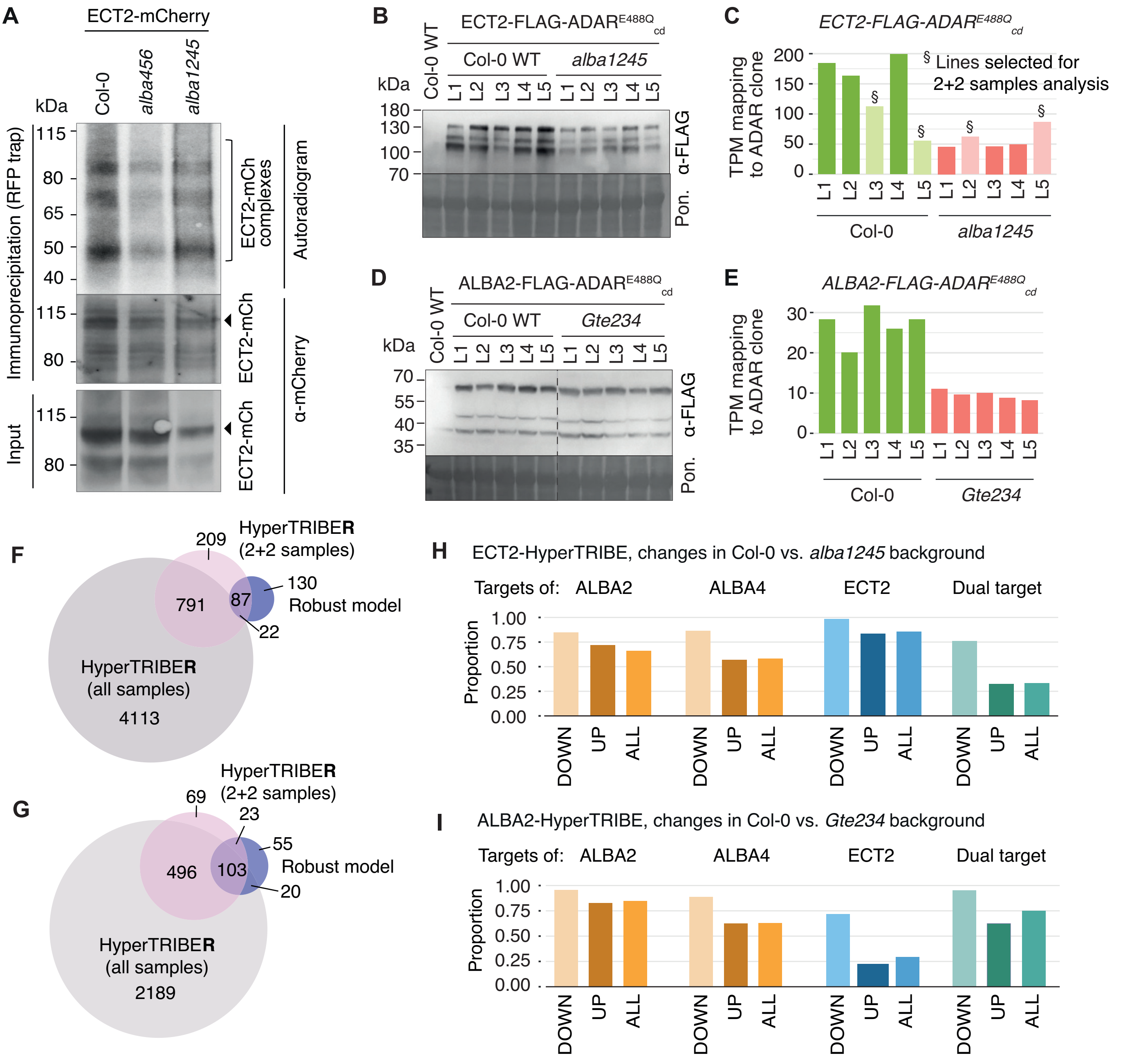


**Figure EV4. Mutual ECT-ALBA dependence for target mRNA binding.**

(**A**) Autoradiogram (top) of RNA-protein complexes from ECT2-mCherry expressed in Col-0, *alba1245*, or *alba456* after UV crosslinking and immunoprecipitation with RFP-trap beads. Marker positions and the location of the ECT2-mCherry-RNA adducts are indicated. Immunoblots against mCherry show the precipitated protein in the IP (middle) and input (bottom). Samples were pools of three independent lines for each genotype. (**B**) Western blot of the independent lines selected for HyperTRIBE analysis of ECT2 in Col-0 and *alba1245*. Ponceau staining was used as a loading control. (**C**) Detected number of transcripts per million of ECT2-FLAG-ADAR in lines used for HyperTRIBE. Light bars indicate lines selected for the two-sample analysis. (**D**) Western blot of the independent lines selected for HyperTRIBE analysis of ALBA2 in Col-0 and *gte234*. Ponceau staining was used as a loading control. (**E**) Detected number of transcripts per million of ALBA2-FLAG-ADAR in lines used for HyperTRIBE. (**F**) Overlap of individual sites detected as significant in ECT2-HT in Col-0 vs. *alba1245* according to three different approaches—HyperTRIBER pipeline (no correction), HyperTRIBER pipeline (using lines indicated in **C**) and developed a robust model. (**G**) As in (**F**) but counts depict containing genes for significant sites. (**H–I**) The proportion of sites significantly differentially edited by ECT2- (**H**) or ALBA2- (**I**) -FLAG-ADAR$^{E488Q}{}_{cd}$ fusions in Col-0 vs. the indicated mutant background (*alba1245* in **H**; *Gte234* in **I**), which are targets of ALBA2, ALBA4, ECT2, or both ALBA4 and ECT2 (dual-targets). UP/DOWN categories are defined according to whether the $\log_2$ fold-change was negative (lower in the mutant background, DOWN) or positive (higher in the mutant background, UP). Source data are available online for this figure.

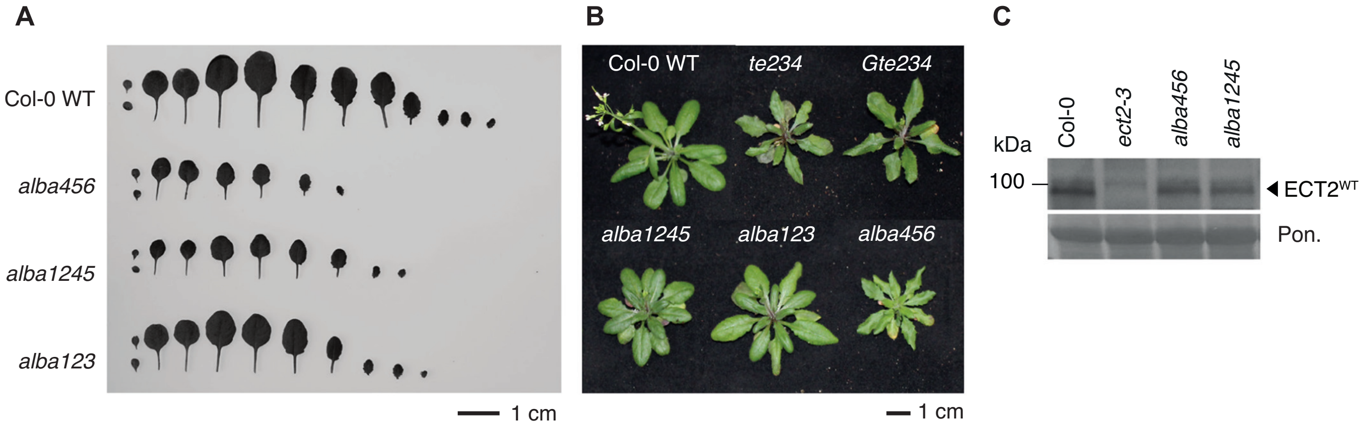


**Figure EV5. Phenotypic analysis of *alba1245* and *alba456* mutants.**

(**A**) Leaf profiles of *alba* mutants at day 17 after germination. (**B**) Delayed flowering phenotype of higher-order *alba* and *ect* mutants (5-week-old, germinated directly on soil). (**C**) Protein blot of total lysates prepared from 10-day-old seedlings of the indicated genotypes, probed with ECT2-specific antisera (Arribas-Hernández et al, 2018). The arrow indicates the position of the ECT2$^{WT}$ protein. Ponceau staining serves as the loading control. Source data are available online for this figure.

 © The Author(s)
